# Design, Synthesis, and Antibacterial Screening of Some Novel Heteroaryl-Based Ciprofloxacin Derivatives as DNA Gyrase and Topoisomerase IV Inhibitors

**DOI:** 10.3390/ph14050399

**Published:** 2021-04-22

**Authors:** Lamya H. Al-Wahaibi, Amer A. Amer, Adel A. Marzouk, Hesham A. M. Gomaa, Bahaa G. M. Youssif, Antar A. Abdelhamid

**Affiliations:** 1 Department of Chemistry, College of Sciences, Princess Nourah Bint Abdulrahman University, Riyadh P.O. Box 84428, Saudi Arabia; lhalwahaibi@pnu.edu.sa; 2 Department of Chemistry, Faculty of Science, Sohag University, Sohag 82524, Egypt; amer_chem@yahoo.com (A.A.A.); drantar25@yahoo.com (A.A.A.); 3 Department of Pharmaceutical Chemistry, Faculty of Pharmacy, Al-Azhar University, Assiut 71524, Egypt; adelmarzouk@azhar.edu.eg; 4 Pharmacology Department, College of Pharmacy, Jouf University, Aljouf 72341, Saudi Arabia; hasoliman@ju.edu.sa; 5 Pharmaceutical Organic Chemistry Department, Faculty of Pharmacy, Assiut University, Assiut 71526, Egypt; 6 Chemistry Department, Faculty of Science, Albaha University, Albaha P.O. Box 1988, Saudi Arabia

**Keywords:** ciprofloxacin, heteroaryl, antibacterial, gyrase, topoisomerase IV

## Abstract

A novel series of ciprofloxacin hybrids comprising various heterocycle derivatives has been synthesized and structurally elucidated using ^1^H NMR, ^13^C NMR, and elementary analyses. Using ciprofloxacin as a reference, compounds **1–21** were screened in vitro against Gram-positive bacterial strains such as *Staphylococcus aureus* and *Bacillus subtilis* and Gram-negative strains such as *Escherichia coli* and *Pseudomonas aeruginosa*. As a result, many of the compounds examined had antibacterial activity equivalent to ciprofloxacin against test bacteria. Compounds **2–6**, oxadiazole derivatives, were found to have antibacterial activity that was 88 to 120% that of ciprofloxacin against Gram-positive and Gram-negative bacteria. The findings showed that none of the compounds tested had antifungal activity against *Aspergillus flavus*, but did have poor activity against *Candida albicans*, ranging from 23% to 33% of fluconazole, with compound **3** being the most active (33% of fluconazole). The most potent compounds, **3**, **4**, **5**, and **6**, displayed an IC_50_ of 86, 42, 92, and 180 nM against *E. coli* DNA gyrase, respectively (novobiocin, IC_50_ = 170 nM). Compounds **4**, **5**, and **6** showed IC_50_ values (1.47, 6.80, and 8.92 µM, respectively) against *E. coli* topo IV in comparison to novobiocin (IC_50_ = 11 µM).

## 1. Introduction

Bacterial infection remains a significant threat to human life due to its increasing resistance to conventional antibiotics, which is a growing public health concern. As a result, there is a critical need to create new antimicrobial agents with potent anti-drug-resistant microorganism activity [1]. That is why antimicrobial agent investigations are so critical and should always be up to date.

**Figure 1:**
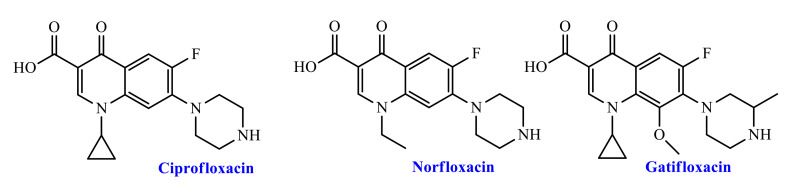
Chemical structures of some FDA approved marketed fluoroquinolone-based antibiotic drugs.

Due to their excellent efficacy, bioavailability, and relatively low toxic and adverse effects, fluoroquinolone (FQ) antibiotics have been one of the most commonly used groups of antibiotics in recent years, (Figure 1). Fluoroquinolones (FQs) are antibiotics that have the ability to treat a range of bacterial infections [2,3]. FQs are inhibitors of *S. aureus* multidrug efflux pumps [4], lower and upper respiratory infections [5], prostatitis, and urinary tract infections (UTI) [6]. Fluoroquinolone antibiotics co-alter the bacterial DNA gyrase and topoisomerase IV enzymes in a hybrid enzyme–DNA complex [7]. Such a change in bacterial enzyme performance inhibits the desired bacterial growth DNA synthesis. FQ drugs have long been known as a favored structural framework for the development of new commercially available drugs [8]. Changes in the elementary structure of FQs are thought to enhance drug interaction with the target enzyme, which may improve pharmacokinetic properties. This type of structural functioning of the FQs may provide stronger drugs for future generations. Substitution at position 7 of the FQ core has a significant impact on solubility, bioavailability, and antimicrobial activities [9]. With the introduction of various C-7 moieties, researchers have focused on improving their biological range. As a result, different moieties were used to examine its biological profile with a piperazinyl ring, substituted piperazine moiety, heterocyclic ring (especially five or six members). Ciprofloxacin is one of the most potent second-generation fluoroquinolones (FQs). It offers potential anti-infective therapy for a wide range of bacteria, Gram-positive, and Gram-negative (Figure 1) [10,11]. For 12 medical treatments, including veterinary uses, the Food and Drug Administration (FDA) approved ciprofloxacin (CP, see Figure 1) which exhibits antibacterial activity with minimal side effects and good pharmacokinetic properties. In addition, ciprofloxacin has a wide range of biological profiles and has been used to examine its antimalarial, anti-fungal, anti-tumor, and antibacterial properties in different areas of medical research [12,13].

Ciprofloxacin is currently used to treat a number of Gram-positive and Gram-negative bacterial infections in clinical practice. However, because of the emergency and widespread of drug-resistant bacteria, ciprofloxacin is becoming increasingly ineffective. As a result, novel antibacterial agents are urgently needed [14].

To combat resistance, the production of novel ciprofloxacin derivatives that are effective against both drug-susceptible and drug-resistant pathogens is crucial. Many ciprofloxacin derivatives have been designed and synthesized with excellent in vitro and in vivo potency against both drug-sensitive and drug-resistant species, including fluoroquinolone-resistant, multidrug-resistant pathogens [15,16,17,18,19].

**Figure 2:**
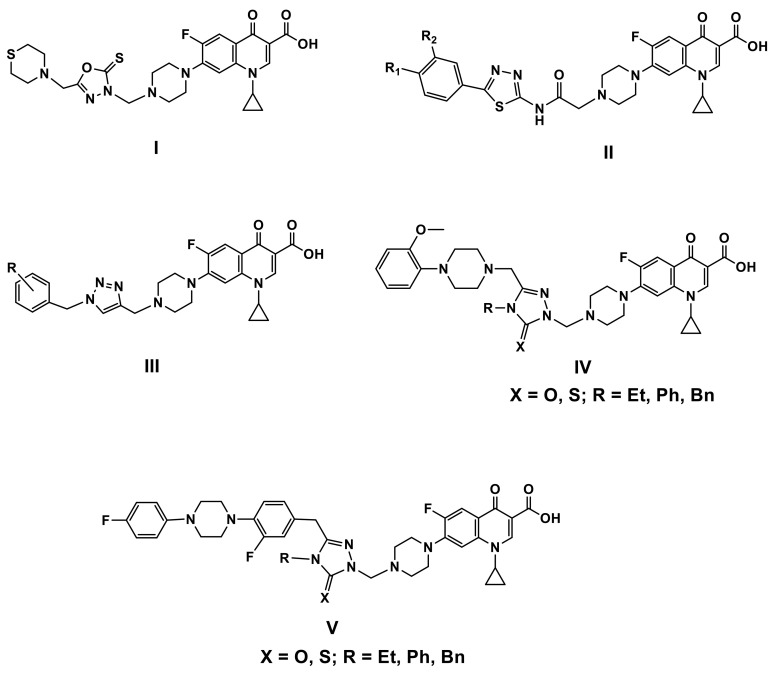
Chemical structures of some ciprofloxacin-azole hybrids **I–V**.

With MICs ranging from 0.28 to 15.8 µM/mL, the 1,3,4-oxadiazole ciprofloxacin hybrid **I** (Figure 2) was >41 times more potent than ampicillin (MIC: 10–128 mg/mL) against most of the pathogens tested [15].

The antibacterial and antifungal activities of the thiadiazole ciprofloxacin hybrids **II** (Figure 2) were only mild to moderate [16].

Agarwal et al. tested a sequence of bis-1,2,3-triazole-ciprofloxacin hybrids **III** (Figure 2) in vitro against a panel of clinically important bacteria [17]. A significant portion of the hybrids showed increased activity against both Gram-positive and Gram-negative bacteria relative to ciprofloxacin, and antibacterial activity appears to be related to the nature and position of substituents, as well as their isomeric effects on phenyl rings. Furthermore, the compounds’ low toxicity profile suggests that they may be useful antibiotics in the future [17].

Demirbas et al. found that the 1,2,4-triazole-5(4*H*)-one/thione ciprofloxacin hybrids **IV** and **V** (Figure 2) with substituted piperazine at the C-3 position of the triazole moiety had promising in vitro activity against both Gram-positive and Gram-negative pathogens with MIC 0.24 mg/mL, which was far more potent than Ampicillin (MIC: 3.9–250 mg/mL) [18,19].

Continuing our quest to find a compound with improved antimicrobial properties [20,21,22,23,24,25,26,27], the current study describes the synthesis and structure elucidation of Fluoroquinolone(ciprofloxacin)-based hybrids containing various heterocycle derivatives, as well as antimicrobial activity evaluation using various strains of Gram-positive (*S. aureus* and *B. subtilis*), Gram-negative (*E. coli* and *P. aeruginosa*), and fungi (*A. flavus* and *C. albicans*). In addition, the inhibitory activity of the most active compounds against *E. coli* DNA gyrase and topoisomerase IV has been identified as a potential molecular target.

## 2. Results and Discussion

### 2.1. Chemistry

**Scheme 1:**
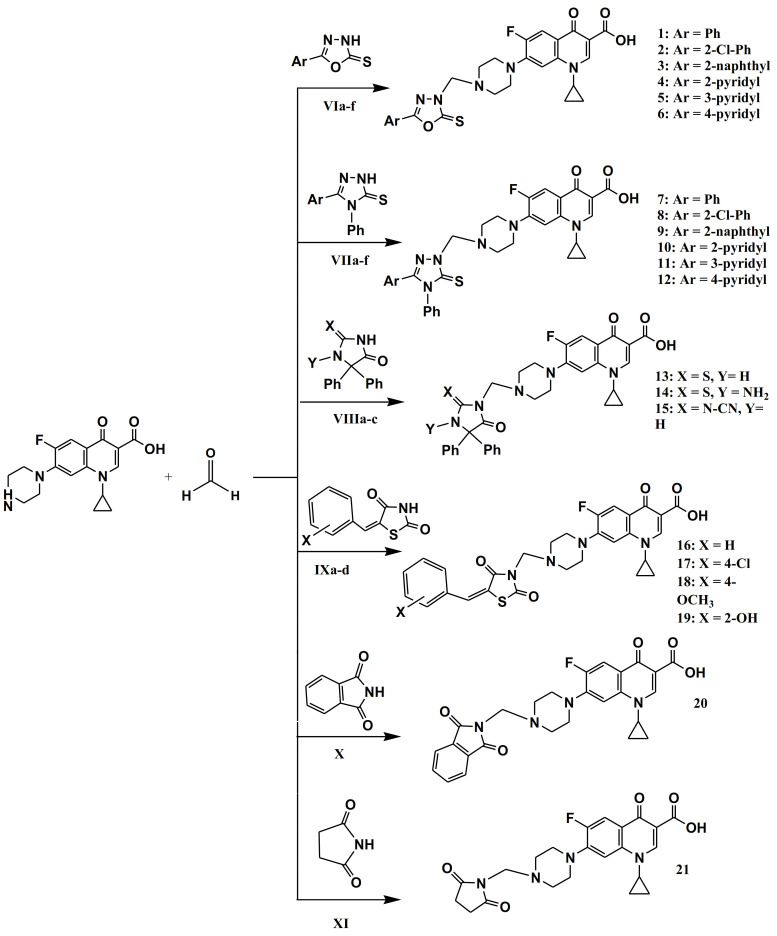
Synthesis of target compounds **1**–**21**. Reagents and conditions: Appropriate heterocyclic compound, EtOH, reflux, 0.5 h, 79–97%.

The synthetic strategy towards the synthesis of target compounds **1–21** is outlined in Scheme 1. The intermediates **VIa–f [28]**, **VIIa–f [29]**, **VIIIa–c [30]**, and **IXa–d [31]** were synthesized, as stated previously, and their structures were confirmed by comparing their physical constants and spectral data to those previously recorded. As shown in Scheme 1, ciprofloxacin undergoes a Mannich reaction with heterocycles **I**–**VI** and formaldehyde in refluxing ethanol to yield the target compounds **1**–**21** in yields ranging from 79% to 97%. The IR spectra of compounds **1**–**21** showed a stretching band at 3381–3310 cm^−1^ related to (OH), a medium stretching band at 3062–3004 of CH aromatic and strong stretching band at 1731–1701 cm^−1^ related to (C=O), which are consistent with the proposed structure. The ^1^H NMR spectra of **1**–**21** revealed the appearance of a methylene signal at 5.19–5.16 (s, 2H, N-CH_2_-N), two sets of triplets at 3.39–3.27 and 3.07–3.03 ppm, which is indicative of piperazinyl protons, three signals at 3.84–3.80 (m, 1H), 1.35–1.30 (q, 2H), and 1.18–1.14 (q, 2H) ppm attributed to cyclopropyl protons, and broad singlet at 15.13–15.05 ppm of the COOH group. Moreover, the ^1^H NMR spectra of **16–19** revealed the appearance of singlet signal at 8.64 ppm of olefinic CH. The ^13^C NMR spectra of **1–21** showed the characteristic methyl carbon (N-C-N) at 70 ppm and the (C=O) at 166–160 and 173–178 ppm. At their predicted chemical shifts were the olefin and aromatic carbons (Supplementary File, Figures S1–S21). The purity of **1–21** has been confirmed using elementary analysis, and the results fit the products’ molecular formula.

### 2.2. Biology

#### 2.2.1. Antimicrobial Sensitivity Test

An updated Kirby-Bauer disc diffusion method was used to assess the antimicrobial activity of the tested samples [32,33,34,35]. Table 1 presents the results of the preliminary antimicrobial testing of final compounds. Using ciprofloxacin as a reference drug, synthetic compounds **1–21** were tested in vitro for antibacterial activity against *S. aureus* and *B. subtilis* as Gram-positive strains and *E. coli* and *P. aeruginosa* as Gram-negative strains. As a result, the majority of newly synthesized compounds demonstrated promising antibacterial activity comparable to ciprofloxacin against test species (Table 1). The oxadiazole derivatives, compounds **2–6** were found to exhibit pronounced antibacterial activity, which ranged from 88% to 120% that of ciprofloxacin against both Gram-positive and Gram-negative strains. It is worth mentioning that compound **6** showed superior activity (120%) against *S. aureus* to that of ciprofloxacin. Oxadiazoles **4** and **5** showed equipotent activity to ciprofloxacin against *S. aureus* and *E. coli*. The thiazolidine derivative **16** had a ciprofloxacin-like activity of 93% against *B. subtilis*, *E. coli*, and *P. aeruginosa*, but only 85% activity against *S. aureus*, Table 1. Compounds **1**, **7–10**, and **17–21** showed moderate antibacterial activity which ranged from 70% to 83% of ciprofloxacin against both Gram-positive and Gram-negative strains.

According to the previous findings, the inclusion of oxadiazole and thiadiazole moiety in a compound confers the highest efficacy.

Furthermore, using fluconazole as a reference drug, **1**–**21** were tested in vitro for antifungal activity against *A. flavus* and *C. albicans* (Table 1). The findings showed that the tested compounds had no antifungal activity against *A. flavus* but had weak antifungal activity against *C. albicans*, ranging from 23% to 33% of fluconazole, with compound **3** being the most active (33% of fluconazole).

#### 2.2.2. Minimum Inhibitory Concentration Test

A two-fold serial dilution method was used to assess the antimicrobial activity of the most active components, oxadiazole-based hybrids, **2–6 [36]**. Table 2, using the reference drug ciprofloxacin, was presented with the MIC_s_ (minimal inhibitory concentrations) of these compounds against tested bacteria. Some new compounds have demonstrated good antimicrobial inhibitory activities for Gram-positive and Gram-negative strains. As shown in Table 2, compounds **4**, **5**, and **6** were the most active and effective against three bacterial strains, in which compound **4** with MIC values of 0.035, 0.062, 0.062 µg/mL against *S. aureus*, *E. coli* and *P. aureoginosa*, compound **5** with MIC values of 0.035, 0.062, 0.125 µg/mL against *S. aureus*, *E. coli* and *P. aureoginosa*, and compound **6** with MIC values of 0.031, 0.125, 0.125 µg/mL against *S. aureus*, *E. coli* and *P. aureoginosa*, respectively. In comparison to ciprofloxacin (MIC of 0.030 g/mL), compound **2** showed the next best activity against *S. aureus* strains with a MIC value of 0.062 g/mL. Interestingly, all the compounds examined only had a minor inhibitory effect on *B. subtilis*, a Gram-positive organism. The nature of aromatic substitution in the oxadiazole moiety tends to be correlated with higher antibacterial effects and the activity increased with (Ar) in the order of 2-pyridyl ≥ 3-pyridyl > 4-pyridyl > 2-Cl-Ph > 2-naphthyl.

#### 2.2.3. Inhibitory Activity Against *E. coli* DNA Gyrase and Topoisomerase IV 

*E. coli* DNA gyrase assay [37] was performed to evaluate the inhibitory potency of oxadiazole-based derivatives **2–6** against *E. coli* DNA gyrase and the results are included in Table 3. Results are presented as residual activities (RAs) of the enzyme at 1 µM of compounds or as IC_50_ values for compounds with RA <50% (Table 3). The results of the antimicrobial activity study are complemented by the results from this assay. Investigated compounds **3**–**6** exhibited inhibition of *E. coli* DNA with IC_50_ ranging from 42 to 180 nM relative to reference novobiocin (IC_50_ = 170 nM). Based on the data provided, compounds **3**, **4**, and **5** were found to be the most active and their inhibitory activities of *E. coli* DNA gyrase assay (IC_50_ = 86 ± 9, 42 ± 7, and 92 ± 9, respectively) were superior to positive control novobiocin.

Compounds **2–6** were further evaluated against *E. coli* topoisomerase IV [37], as shown in Table 3. Compounds **4**, **5,** and **6**, which were among the most potent inhibitors of *E. coli* gyrase also displayed promising results on topoisomerase IV (Table 3). Compounds **4**, **5**, and **6** had IC_50_ values = 1.47, 6.80, and 8.92 µM, respectively, which are much lower (more potent) than that for novobiocin (IC_50_ = 11 µM). From these findings, both **4** and **5**, after optimization, appear to be promising dual target inhibitors against DNA gyrase and topoisomerase IV.

#### 2.2.4. Cell Viability Assay

A human mammary gland epithelial cell line (MCF-10A) was used to conduct a cell viability assay [38]. Compounds **2–6** were incubated with MCF-10A cells for four days, and the viability of the cells was determined using the 3-(4,5-dimethylthiazol-2-yl)-2,5-diphenyltetrazolium bromide (MTT) assay [39]. All compounds had no cytotoxic effects, and the viability of the cells was greater than 85% for most of the compounds examined at 50 M, as shown in Table 4.

### 2.3. Drug Likeness Profile

Absorption, delivery, metabolism, and excretion (ADME) testing became popular early in drug development programs, with computer models serving as feasible alternatives to experiments. The Swiss ADME website was used to predict the drug likeness profile of the studied compounds 1–21 [40,41]. Tables S1–S3 (Supplementary File) display the results of the drug likeness profile of these compounds. Many compounds, such as **1**, **13**, **14**, **16**, **17**, **20**, and **21**, were expected to have high oral absorption. However, due to the high molecular weight (522) and molar refractivity (143), the others predicted poor oral absorption. Some of the tested compounds as 20 and 21 showed no violation to Lipinski (Pfizer) filters, except for one violation for compounds 1–8 and 11–19. In addition two violation for compounds 9 and 10 (molecular weight >500 [42]) and compound 21 showed no violations to Ghose in addition to two violations for 1–6, three violations for 7, 8, 11, 12, 13, 14 and four violations for 9 and 10 due to high WLOGP and molecular weight [43], no violation to Veber (GSK) for all compounds except one violation for 4, 5, 6, 14, 15, 19 [44], no violation to Egan (Pharmacia) for all compounds except one violation for 4, 5, 6, 14, 15, 18, 19 [45] and no violation to Muegge (Bayer) except one violation for compounds 1, 8, 13, 14, and two violations for 9, 10 [46] filters. The compounds were free from alerts for Pan Assay Interfering substances (PAINS) [47]. Total polar surface area (TPSA) values for most compounds are 103.16–148.69, as shown in Table 1, Table 2 and Table 3. This consists of “good GIT absorption”. There is a correlation between the molecular weight of compounds and their activity. In addition to low rigidity, this pattern highlighted low molecular weight is favorable. Lipophilicity, together with the molecular weight and the number of hydrogen bond donors and the number of hydrogen acceptors shown by these compounds, plays the role of five (see Table S1, Supplementary File).

## 3. Materials and Methods

### 3.1. Chemistry

General Details: See Appendix A

#### 3.1.1. General Procedure for the Synthesis of Compounds **1**–**21**

To a mixture of ciprofloxacin HCl (1.28 g, 0.003 mol), formaldehyde (0.2 g, 0.007 mol) and different heterocyclic compounds (0.003 mol) were dissolved in 10 mL of ethanol. The reaction mixture was stirred with reflux for 30 min. The reaction mixture was allowed to cool at room temperature, the separated solid was filtered off, washed with water, and crystallized from methanol.

##### 1-Cyclopropyl-6-fluoro-4-oxo-7-[4-(5-phenyl-2-thioxo[1,3,4]oxadiazol-3-yl-methyl)-piperazin-1-yl]-1,4-dihydro-quinoline-3-carboxylic acid (**1**)

Yield (94%); m.p. 228 °C; IR: 3350 (OH), 3049 (CH aromatic), 2987, 2946 (CH-aliphatic), 1710 (C=O), 1615 (C=N); ^1^H NMR: δ 15.13 (brs. OH, COOH), 8.65–7.58 (m, 7H, aromatic), 5.16 (s, 2H, N-CH_2_-N), 3.83–3.80 (m, J = 2.44 Hz, 1H, CH_2_-CH-CH_2_ cyclopropyl-H), 3.38 (t, 4H, piperazinyl-H), 3.04 (t, 4H, piperazinyl-H), 1.34–1.32 (q, J = 3.54 Hz, 2H, CH_2_-CH-CH_2_ cyclopropyl-H), 1.18–1.15 (q, J = 4.08 Hz, 2H, CH_2_-CH-CH_2_cyclopropyl-H); ^13^C NMR: δ 178.82., 166.40, 148.28. 145.45, 139.64, 129.88, 111.52, 107.27, 106.96, 70.24, 51.05 49.82, 49.70, 36.27, 8.02. C_26_H_24_FN_5_O_5_S: C, 59.87; H, 4.64; N, 13.43, Found: C, 59.81; H, 4.62; N, 13.34.

##### 7-{4-[5-(2-Chloro-phenyl)-2-thioxo-[1,3,4]oxadiazol-3-ylmethyl]-piperazin-1-yl}-1-cyclopropyl-6-fluoro-4-oxo-1,4-dihydro-quinoline-3-carboxylic acid (*2*)

Yield (85%); m.p. 226 °C; IR: 3354 (OH), 3062 (CH aromatic), 2999, 2914 (CH-aliphatic), 1719 (C=O), 1614 (C=N); ^1^H NMR: δ 15.11 (brs. OH, COOH), 8.65–7.65 (m, 7H, aromatic), 5.19 (s, 2H, N-CH_2_-N), 3.84–3.80 (m, J = 3.52 Hz, 1H, CH_2_-CH-CH_2_ cyclopropyl-H), 3.39 (t, 4H, piperazinyl-H), 3.03 (t, 4H, piperazinyl-H), 1.35–1.30 (q, J = 5.44 Hz, 2H, CH_2_-CH-CH_2_ cyclopropyl-H), 1.18–1.15 (q, J = 5.64 Hz, 2H, CH_2_-CH-CH_2_cyclopropyl-H); ^13^C NMR: δ 173.59., 168.75, 166.45, 131.73, 128.38, 111.55, 111.30, 110.09, 109.37, 108.30, 107.17, 70.26, 49.84, 49.62, 36.33, 8.03. C_26_H_23_ClFN_5_O_5_S: C, 56.16; H, 4.17; N, 12.60, Found: C, 56.08; H, 4.02; N, 12.34.

##### 1-Cyclopropyl-6-fluoro-7-[4-(5-naphthalen-2-yl-2-thioxo-[1,3,4]oxadiazol-3-yl methyl)-piperazin-1-yl]-4-oxo-1,4-dihydro-quinoline-3-carboxylic acid (**3**)

Yield (96%); m.p. 244 °C; IR: 3367 (OH), 3019 (CH aromatic), 2961, 2957 (CH-aliphatic), 1708 (C=O), 1609 (C=N); ^1^H NMR: δ 15.11 (brs. OH, COOH), 8.66–7.55 (m, 10H, aromatic), 5.19 (s, 2H, N-CH2-N), 3.83–3.79 (m, J = 5.51 Hz, 1H, CH_2_-CH-CH_2_ cyclopropyl-H), 3.39 (t, 4H, piperazinyl-H), 3.07 (t, 4H, piperazinyl-H), 1.35–1.30 (q, 4H, J = 5.69 Hz, CH_2_-CH-CH_2_ cyclopropyl-H); ^13^C NMR: δ 176.74, 166.27, 148.44, 139.39 122.25, 111.72, 107.27, 49.91, 49.79, 36.29, 8.03. C_30_H_26_FN_5_O_5_S: C, 63.03; H, 4.58; N, 12.25, Found: C, 62.88; H, 4.62; N, 12.01.

##### 1-Cyclopropyl-6-fluoro-4-oxo-7-[4-(5-pyridin-2-yl-2-thioxo-[1,3,4]oxadiazol-3-yl-methyl)-piperazin-1-yl]-1,4-dihydro-quinoline-3-carboxylic acid (**4**)

Yield (89%); m.p. 218 °C; IR: 3369 (OH), 3049 (CH aromatic), 2967, 2924 (CH-aliphatic), 1721 (C=O), 1619 (C=N); ^1^H NMR: δ 15.10 (brs. OH, COOH), 9.07–7.55 (m, 7H, aromatic), 5.17 (s, 2H, N-CH_2_-N), 3.80 (m, J = 2.8 Hz, 1H, CH_2_-CH-CH_2_ cyclopropyl-H), 3.27 (t, 4H, piperazinyl-H), 3.04 (t, 4H, piperazinyl-H), 1.30–1.35 (q, J = 5.20 Hz, 2H, CH_2_-CH-CH_2_ cyclopropyl-H), 1.14–1.18 (q, J = 6.92 Hz, 2H, CH_2_-CH-CH_2_cyclopropyl-H); ^13^C NMR: δ 176.80., 166.34, 148.40. 142.31, 139.62, 124.25, 119.74, 111.25, 107.25, 107.00, 70.32, 49.84, 49.69, 36.28, 18.95 8.02. C_25_H_24_FN_6_O_5_S: C, 57.46; H, 4.44; N, 16.08, Found: C, 57.29; H, 4.31; N, 16.01.

##### 1-Cyclopropyl-6-fluoro-4-oxo-7-[4-(5-pyridin-3-yl-2-thioxo-[1,3,4]oxadiazol-3-yl-methyl)-piperazin-1-yl]-1,4-dihydro-quinoline-3-carboxylic acid (**5**)

Yield (92%); m.p. 230 °C; IR: 3310 (OH), 3019 (CH aromatic), 2984, 2902 (CH-aliphatic), 1707 (C=O), 1609 (C=N); ^1^H NMR: δ 15.11 (brs. OH, COOH), 8.77–7.55 (m, 1H, aromatic), 5.18 (s, 2H, N-CH_2_-N), 3.82–3.80 (m, J = 1.80 Hz, 1H, CH_2_-CH-CH_2_ cyclopropyl-H), 3.27(t, 4H, piperazinyl-H), 3.04 (t, 4H, piperazinyl-H), 1.33–1.32 (q, J = 1.96 Hz, 2H, CH_2_-CH-CH_2_ cyclopropyl-H), 1.17–1.16 (q, J = 1.76 Hz, 2H, CH_2_-CH-CH_2_cyclopropyl-H); ^13^C NMR: δ 176.73, 166.41, 154.62, 152.14, 150.78, 148.37, 145.40, 139.55, 138.32, 127.27, 123.06, 118.96, 111.44, 111.22, 107.13, 106.96, 70.30, 49.79, 49.65, 43.21, 36.28, 18.98, 8.02. C_25_H_24_FN_6_O_5_S: C, 57.46; H, 4.44; N, 16.08, Found: C, 57.33; H, 4.40; N, 16.41.

##### 1-Cyclopropyl-6-fluoro-4-oxo-7-[4-(5-pyridin-4-yl-2-thioxo-[1,3,4]oxadiazol-3-yl-methyl)-piperazin-1-yl]-1,4-dihydro-quinoline-3-carboxylic acid (**6**)

Yield (93%); m.p. 208 °C; IR: 3351 (OH), 3004 (CH aromatic), 2995, 2913 (CH-aliphatic), 1731 (C=O), 1607 (C=N); ^1^H NMR: δ 15.05 (brs. OH, COOH), 8.81–7.57 (m, 7H aromatic), 5.17 (s, 2H, N-CH_2_-N), 3.82 (m, J = 3 Hz, 1H, CH_2_-CH-CH_2_ cyclopropyl-H), 3.22(t, 4H, piperazinyl-H), 3.05 (t, 4H, piperazinyl-H), 1.35–1.33 (q, J = 4.56 Hz, 2H, CH_2_-CH-CH_2_ cyclopropyl-H), 1.19–1.17 (q, J = 3.04 Hz, 2H, CH_2_-CH-CH_2_cyclopropyl-H); ^13^C NMR: δ 178.81., 166.31, 157.70, 154.65, 151.34, 140.37, 139.62, 130.27, 120.07, 119.08, 111.51, 111.28, 107.29, 106.99, 70.28, 49.84, 49.68, 36.27, 8.03. C_25_H_24_FN_6_O_5_S: C, 57.46; H, 4.44; N, 16.08, Found: C, 57.14; H, 4.39; N, 16.15.

##### 1-Cyclopropyl-7-[4-(3,4-diphenyl-5-thioxo-4,5-dihydro-[1,2,4]triazol-1-ylmethyl)-piperazin-1-yl]-6-fluoro-4-oxo-1,4-dihydro-quinoline-3-carboxylic acid (**7**)

Yield (93%); m.p. 288 °C; IR: 3381 (OH), 3014 (CH aromatic), 2961, 2943, 2906 (CH-aliphatic), 1707 (C=O), 1598 (C=N); ^1^H NMR: δ 15.12 (brs. OH, COOH), 8.66–7.36 (m, 13H, aromatic), 5.30 (s, 2H, N-CH_2_-N), 3.80–3.85 (m, J = 7.64 Hz, 1H, CH_2_-CH-CH_2_ cyclopropyl-H), 3.3.41 (t, 4H, piperazinyl-H), 3.09 (t, 4H, piperazinyl-H), 1.31–1.36 (q, J = 7.45 Hz, 2H, CH_2_-CH-CH_2_ cyclopropyl-H), 1.19–1.15 (q, J = 6.76 Hz, 2H, CH_2_-CH-CH_2_cyclopropyl-H); ^13^C NMR: δ 177.32, 173.40, 172.02, 170.78, 170.66, 150.17, 149.38, 148.54, 143.74, 136.56, 135.68, 135.68, 134.41, 129.07, 128.89, 58.17, 52.68, 50.08, 50.08, 10.64. C_32_H_29_FN_6_O_3_S: C, 64.41; H, 4.90; N, 14.08, Found: C, 64.22; H, 4.81; N, 14.02.

##### 7-{4-[3-(2-Chloro-phenyl)-4-phenyl-5-thioxo-4,5-dihydro-[1,2,4]triazol-1-ylmethyl]-piperazin-1-yl}-1-cyclopropyl-6-fluoro-4-oxo-1,4-dihydro-quinoline-3-carboxylic acid (**8**)

Yield (96%); m.p. 214 °C; IR: 3332 (OH), 3056 (CH aromatic), 2984, 2917 (CH-aliphatic), 1701 (C=O), 1614 (C=N); ^1^H NMR: δ 15.13 (brs. OH, COOH), 8.67–7.33 (m, 12H, aromatic), 5.33 (s, 2H, N-CH_2_-N), 3.84–3.83 (m, J = 3.00 Hz, 1H, CH_2_-CH-CH_2_ cyclopropyl-H), 3.42 (t, 4H, piperazinyl-H), 3.07 (t, 4H, piperazinyl-H), 1.34–1.19 (q, J = 1.40 Hz, 4H, CH_2_-CH-CH_2_ cyclopropyl-H); ^13^C NMR: δ 176.84, 169.33, 166.44, 148.51, 147.50, 145.60, 134.35, 133.45, 129.55, 129.45, 128.57, 127.04, 111.57, 107.19, 107.04, 69.24, 50.06, 49.90, 36.33, 8.08. C_32_H_28_ClFN_6_O_3_S: C, 60.90; H, 4.47; N, 13.32, Found: C, 60.59; H, 4.28; N, 13.39.

##### 1-Cyclopropyl-6-fluoro-7-[4-(3-naphthalen-2-yl-4-phenyl-5-thioxo-4,5-dihydro-[1,2,4]triazol-1-ylmethyl)-piperazin-1-yl]-4-oxo-1,4-dihydro-quinoline-3-carboxylic acid (**9**)

Yield (97 %); m.p. 262 °C; IR: 3365 (OH), 3041 (CH aromatic), 2964, 2921 (CH-aliphatic), 1714 (C=O), 1612 (C=N); ^1^H NMR: δ 15.14 (brs. OH, COOH), 8.65–7.43 (m, 15H, aromatic), 5.34 (s, 2H, N-CH_2_-N), 3.82 (m, CH_2_-CH-CH_2_ cyclopropyl-H), 3.42 (t, 4H, piperazinyl-H), 3.12 (t, 4H, piperazinyl-H), 1.33–1.31 (q, J = 0.74 Hz, 2H, CH_2_-CH-CH_2_ cyclopropyl-H), 1.17–1.16 (q, J = 1.52 Hz, 2H, CH_2_-CH-CH_2_cyclopropyl-H); ^13^C NMR: δ 176.81, 170.25, 166.34, 149.70, 148.41, 145.70, 139.62, 135.80, 133.74, 132.42, 130.04, 129.86, 129.28, 129.12, 128.63, 128.25, 127.56, 123.18, 111.53, 111.30, 107.25, 106.97, 69.41, 50.16, 49.96, 36.29, 8.04. C_36_H_31_FN_6_O_3_S: C, 66.86; H, 4.83; N, 12.99, Found: C, 66.49; H, 4.69; N, 12.58.

##### 1-Cyclopropyl-6-fluoro-4-oxo-7-[4-(4-phenyl-3-pyridin-2-yl-5-thioxo-4,5-dihydro-[1,2,4]triazol-1-ylmethyl)-piperazin-1-yl]-1,4-dihydro-quinoline-3-carboxylic acid (**10**)

Yield (96%); m.p. 270 °C; IR: 3371 (OH), 3095 (CH aromatic), 2996, 2915 (CH-aliphatic), 1704 (C=O), 1611 (C=N); ^1^H NMR: δ 15.12 (brs. OH, COOH), 8.63–7.34 (m, 12H, aromatic), 5.33 (s, 2H, N-CH_2_-N), 3.82–3.79 (m, J = 3.88 Hz, 1H, CH_2_-CH-CH_2_ cyclopropyl-H), 3.40(t, 4H, piperazinyl-H), 3.08 (t, 4H, piperazinyl-H), 1.35–1.30 (q, J = 6.49 Hz, 2H, CH_2_-CH-CH_2_ cyclopropyl-H), 1.18–1.15 (q, J = 6.44 Hz, 2H, CH_2_-CH-CH_2_cyclopropyl-H); ^13^C NMR: δ 176.80, 170.87, 166.33, 154.88, 152.20, 149.73, 148.67, 148.31, 145.60, 145.28, 139.82, 137.72, 136.05, 129.41, 129.24, 128.87, 125.41, 124.65, 119.08, 111.52, 111.25, 107.33, 106.89, 69.45, 50.16 49.93, 49.53, 36.26, 8.03. C_31_H_28_FN_7_O_3_S: C, 62.30; H, 4.72; N, 16.41, Found: C, 62.04; H, 4.61; N, 16.21.

##### 1-Cyclopropyl-6-fluoro-4-oxo-7-[4-(4-phenyl-3-pyridin-3-yl-5-thioxo-4,5-dihydro-[1,2,4]triazol-1-ylmethyl)-piperazin-1-yl]-1,4-dihydro-quinoline-3-carboxylic acid (**11**)

Yield (83%); m.p. 280 °C; IR: 3318 (OH), 3040 (CH aromatic), 2957, 2924 (CH-aliphatic), 1719 (C=O), 1608 (C=N); ^1^H NMR: δ 15.14 (brs. OH, COOH), 8.66–7.41 (m, 1H, aromatic), 5.32 (s, 2H, N-CH_2_-N), 3.83 (m, 1H, CH_2_-CH-CH_2_ cyclopropyl-H), 3.41–3.40 (t, J = 1.44 Hz, 4H, piperazinyl-H), 3.11–3.10 (t, J = 1.72 Hz, 4H, piperazinyl-H), 1.34–1.32 (q, J = 2.16 Hz, 2H, CH_2_-CH-CH_2_ cyclopropyl-H), 1.18 (q, J = 0.84 Hz, 2H, CH_2_-CH-CH_2_cyclopropyl-H); ^13^C NMR: δ 176.81, 170.23, 16.40, 166.40, 149.29, 148.46, 147.66, 145.57, 139.62, 136.53, 135.13, 130.23, 123.92, 122.40, 119.15, 111.52, 107.20, 107.02, 69.39, 50.04, 49.92, 36.32, 19.02, 8.05. C_31_H_28_FN_7_O_3_S: C, 62.30; H, 4.72; N, 16.41, Found: C, 62.14; H, 4.15; N, 16.20.

##### 1-Cyclopropyl-6-fluoro-4-oxo-7-[4-(4-phenyl-3-pyridin-4-yl-5-thioxo-4,5-dihydro-[1,2,4]triazol-1-ylmethyl)-piperazin-1-yl]-1,4-dihydro-quinoline-3-carboxylic acid (**12**)

Yield (91%); m.p. 227 °C; IR: 3321 (OH), 3021(CH aromatic), 2968, 2904 (CH-aliphatic), 1717 (C=O), 1607 (C=N);^1^H NMR: δ 15.06 (brs. OH, COOH), 8.65–7.29 (m, 12H, aromatic), 5.32 (s, 2H, N-CH_2_-N), 3.83–3.81 (m, J = 3.12 Hz, 1H, CH_2_-CH-CH_2_ cyclopropyl-H), 3.41(t, 4H, piperazinyl-H), 3.10 (t, 4H, piperazinyl-H), 1.34–1.32 (q, J = 1.48 Hz, 2H, CH_2_-CH-CH_2_ cyclopropyl-H), 1.18–1.16 (q, J = 2.52 Hz, 2H, CH_2_-CH-CH_2_cyclopropyl-H); ^13^C NMR: δ 178.82, 170.70, 166.35, 150.57, 148.43, 147.57, 145.84, 135.84, 135.13, 133.34, 130.37, 130.00, 125.14, 122.57, 111.53, 107.28, 106.99, 67.53, 50.08, 49.94, 36.30, 19.00, 8.04. C_31_H_28_FN_7_O_3_S: C, 62.30; H, 4.72; N, 16.41, Found: C, 62.16; H, 4.53; N, 16.34.

##### 1-Cyclopropyl-6-fluoro-4-oxo-7-[4-(5-oxo-4,4-diphenyl-2-thioxo-imidazolidin-1-ylmethyl)-piperazin-1-yl]-1,4-dihydro-quinoline-3-carboxylic acid (**13**)

Yield (84%); m.p. 217 °C; IR: 3412 (NH), 3315 (OH), 3031 (CH aromatic), 2981, 2914 (CH-aliphatic), 1721 (C=O), 1601 (C=N); ^1^H NMR: δ 15.07 (brs. OH, COOH), 11.65 (brs. NH, NH imidazolyl), 8.61–7.35 (m, 13H, aromatic), 4.85 (s, 2H, N-CH_2_-N), 3.79–3.77 (m, 1H, CH_2_-CH-CH_2_ cyclopropyl-H), 3.49 (t, 4H, piperazinyl-H), 2.83 (t, 4H, piperazinyl-H), 1.31–1.06 (m, J = 4.48 Hz, 4H, CH_2_-CH-CH_2_ cyclopropyl-H); ^13^C NMR: δ 182.95, 175.24, 166.35, 166.36, 139.51, 138.61, 129.28, 128.91, 121.88, 118.91, 111.54, 107.29, 106.12, 71.74, 62.54, 65.53, 49.84, 36.22, 18.93, 8.82. C_33_H_30_FN_5_O_4_S: C, 64.80; H, 4.94; N, 11.45, Found: C, 64.64; H, 4.77; N, 11.41.

##### 7-[4-(3-Amino-5-oxo-4,4-diphenyl-2-thioxo-imidazolidin-1-ylmethyl)-piperazin-1-yl]-1-cyclopropyl-6-fluoro-4-oxo-1,4-dihydro-quinoline-3-carboxylic acid (**14**)

Yield (79%); m.p. 234 °C; IR: 3391, 3300 (NH_2_), 3259 (OH), 3098 (CH aromatic), 2984, 2912, 2847 (CH-aliphatic), 1708 (C=O), 1601 (C=N); ^1^H NMR: δ 11.81 (brs. OH, COOH), 9.80–9.66 (brs. 2H, NH_2_), 8.41–7.01 (m, 13H, aromatic), 5.14 (s, 2H, N-CH_2_-N), 3.14 (m, 1H, CH_2_-CH-CH_2_ cyclopropyl-H), 2.95–2.74 (t, 4H, piperazinyl-H), 2.26 (t, 4H, piperazinyl-H), 1.04–0.86 (q, 4H, CH_2_-CH-CH_2_ cyclopropyl-H); ^13^C NMR: δ 176.48, 171.24, 166.32, 154.68, 152.31, 151.54, 141.48, 133.11, 131.43, 131.14, 129.39, 129.11, 122.34, 118.41, 117.42, 116.92, 116.69, 67.24, 61.59, 59.34, 54.43, 51.33, 31.31, 23.39, 22.44, 9.11, 8.91; C_33_H_31_FN_6_O_4_S: C, 63.24; H, 4.99; N, 13.41, Found: C, 63.12; H, 4.86; N, 13.37.

##### 7-[4-(2-Cyanoimino-5-oxo-4,4-diphenyl-imidazolidin-1-ylmethyl)-piperazin-1-yl]-1-cyclopropyl-6-fluoro-4-oxo-1,4-dihydro-quinoline-3-carboxylic acid (**15**)

Yield (95%); m.p. 230 °C; IR:3385 (NH), 3330 (OH), 3013 (CH aromatic), 2984, 2901 (CH-aliphatic), 2231 (CN), 1714 (C=O), 1602 (C=N); ^1^H NMR: δ 15.06 (brs. OH, COOH), 11.20 (brs. NH, NH-CN), 8.63–7.40 (m, 13H, aromatic), 4.61 (s, 2H, N-CH_2_-N), 3.37 (m, 1H, CH_2_-CH-CH_2_ cyclopropyl-H), 3.33 (t, 4H, piperazinyl-H), 2.76 (t, 4H, piperazinyl-H), 1.31–1.11 (q, 2H, CH_2_-CH-CH_2_ cyclopropyl-H); ^13^C NMR: δ 194.38, 176.78, 166.31, 148.31, 145.35, 138.65, 129.32, 121.36, 111.55, 106.16, 62.81, 58.11, 49.16, 36.38, 8.85: C_34_H_30_FN_7_O_4_: C, 65.90; H, 4.88; N, 15.82; Found: C, 65.71; H, 4.78; N, 15.77.

##### 7-[4-(5-Benzylidene-2,4-dioxo-thiazolidin-3-ylmethyl)-piperazin-1-yl]-1-cyclo-propyl-6-fluoro-4-oxo-1,4-dihydro-quinoline-3-carboxylic acid (**16**)

Yield (97%); m.p. 237 °C; IR: 3314 (OH), 3017 (CH aromatic), 2984, 2946 (CH-aliphatic), 1711 (C=O), 1603 (C=N); ^1^H NMR: δ 15.10 (brs. OH, COOH), 8.64 (s, 1H, CH=C, CH olefinic) 8.61–7.49 (m, 8H, aromatic), 4.49 (s, 2H, N-CH_2_-N), 3.81–3.77 (m, J = 8.00 Hz, 1H, CH_2_-CH-CH_2_ cyclopropyl-H), 3.46–3.36 (t, 4H, piperazinyl-H), 3.81–2.73 (t, 4H, piperazinyl-H), 1.35–1.32 (q, J = 1.42 Hz, 2H, CH_2_-CH-CH_2_ cyclopropyl-H), 1.19–1.07 (q, J = 2.50 Hz, 2H, CH_2_-CH-CH_2_ cyclopropyl-H); ^13^C NMR: δ 176.34, 168.96, 166.31, 154.63, 152.15, 148.28, 145.58, 139.55, 133.46, 131.81, 130.51, 129.28, 118.91, 111.45, 111.22, 107.21, 106.16, 63.51, 56.53, 50.11, 49.43, 36.25, 18.95, 8.86. C_28_H_25_FN_4_O_5_S: C, 61.30; H, 4.59; N, 10.21, found: C, 61.25; H, 4.52; N, 10.10.

##### 7-{4-[5-(4-Chloro-benzylidene)-2,4-dioxo-thiazolidin-3-ylmethyl]-piperazin-1-yl}-1-cyclopropyl-6-fluoro-4-oxo-1,4-dihydro-quinoline-3-carboxylic acid (**17**)

Yield (96%); m.p. 244 °C; IR: 3290 (OH), 3008 (CH aromatic), 2994, 2944 (CH-aliphatic), 1711 (C=O), 1611 (C=N); δ 15.12 (brs. OH, COOH), 8.64 (s, 1H, CH=C, CH olefinic) 7.91–7.54 (m, 7H, aromatic), 4.70 (s, 2H, N-CH_2_-N), 3.82–3.80 (m, J = 4.00 Hz, 1H, CH_2_-CH-CH_2_ cyclopropyl-H), 3.45–3.34 (t, 4H, piperazinyl-H), 2.82–2.75 (t, 4H, piperazinyl-H), 1.34–1.31 (q, J = 4.00 Hz, 2H, CH_2_-CH-CH_2_ cyclopropyl-H), 1.20–1.07 (q, J = 8.00 Hz, 2H, CH_2_-CH-CH_2_ cyclopropyl-H); ^13^C NMR: δ 176.19, 167.16,166.30, 148.31, 145.53, 139.61, 132.13, 129.88, 119.15, 111.51, 111.21, 107.25, 106.96, 63.62, 59.15, 49.85, 36.28, 18.99, 8.82; C_28_H_24_ClFN_4_O_5_S: C, 57.68; H, 4.15; N, 9.61, found: C, 57.52; H, 4.04; N, 9.53.

##### 1-Cyclopropyl-6-fluoro-7-{4-[5-(4-methoxy-benzylidene)-2,4-dioxo-thiazolidin-3-ylmethyl]-piperazin-1-yl}-4-oxo-1,4-dihydro-quinoline-3-carboxylic acid (**18**)

Yield (92%); m.p. 251 °C; IR: 3349 (OH), 3014 (CH aromatic), 2988, 2908 (CH-aliphatic), 1720 (C=O), 1607 (C=N); ^1^H NMR: δ 15.10 (brs. OH, COOH), 8.64 (s, 1H, CH=C, CH olefinic) 8.62–7.09 (m, 7H, aromatic), 4.68 (s, 2H, N-CH_2_-N), 3.83(s, 3H, OCH_3_), 3.47 (m, 1H, CH_2_-CH-CH_2_ cyclopropyl-H), 3.33–3.09 (t,4H, piperazinyl-H), 3.81–2.72 (t, 4H, piperazinyl-H), 1.35–1.32 (q, 2H, CH_2_-CH-CH_2_ cyclopropyl-H), 1.33–1.05 (q, 2H, CH_2_-CH-CH_2_ cyclopropyl-H); ^13^C NMR: δ 176.78, 169.81, 167.35, 166.33, 166.01 152.16, 148.39, 145.49, 139.54, 133.52, 132.69, 125.94, 119.19, 118.62, 115.45, 111.49, 111.36, 107.36, 106.91, 63.37, 56.54, 55.91, 54.21, 49.84, 36.26, 19.88, 8.82. C_29_H_27_FN_4_O_6_S: C, 60.20; H, 4.70; N, 9.68, found: C, 60.13; H, 4.64; N, 9.53.

##### 1-Cyclopropyl-6-fluoro-7-{4-[5-(2-hydroxy-benzylidene)-2,4-dioxo-thiazolidin-3-ylmethyl]-piperazin-1-yl}-4-oxo-1,4-dihydro-quinoline-3-carboxylic acid (**19**)

Yield (94%); m.p. 244 °C; IR: 3410, 3312 (2OH), 3005 (CH aromatic), 2951, 2910 (CH-aliphatic), 1723 (C=O), 1603 (C=N); ^1^H NMR: δ 15.10 (brs. OH, COOH), 10.51 (brs. OH, PhOH), 8.63 (s, 1H, CH=C, CH olefinic) 8.61–6.92 (m, 7H, aromatic), 4.67 (s, 2H, N-CH_2_-N), 3.81–3.79 (m, J = 4.00 Hz, 1H, CH_2_-CH-CH_2_ cyclopropyl-H), 3.81–3.77 (t, 4H, piperazinyl-H), 2.81–2.74 (t, 4H, piperazinyl-H), 1.33–1.09 (m, 4H, CH_2_-CH-CH_2_cyclopropyl-H); ^13^C NMR: δ 176.73, 169.24, 167.45, 166.32, 157.88, 154.61, 152.13, 148.26, 145.49, 145.39, 139.54, 132.94, 128.41, 128.11, 119.85, 116.67, 111.45, 111.22, 107.22, 106.84, 63.53, 56.51, 50.21,49.81, 46.15, 36.24, 8.88. C_28_H_25_FN_4_O_6_S: C, 59.57; H, 4.46; N, 9.92; Found: C, 59.51; H, 4.40; N, 9.78.

##### 1-Cyclopropyl-7-[4-(1,3-dioxo-1,3-dihydro-isoindol-2-ylmethyl)-piperazin-1-yl]-6-fluoro-4-oxo-1,4-dihydro-quinoline-3-carboxylic acid (**20**)

Yield (89%); m.p. 241 °C; IR: 3341 (OH), 3023 (CH aromatic), 2998, 2812 (CH-aliphatic), 1753, 1721 (2 C=O), 1611 (C=N); ^1^H NMR: δ 15.11 (brs. OH, COOH), 8.64–7.50 (m, 7H, aromatic), 4.56 (s, 2H, N-CH_2_-N), 3.81–3.77 (m, J = 3.12 Hz, 1H, CH_2_-CH-CH_2_ cyclopropyl-H), 3.42 (t, 4H, piperazinyl-H), 2.91 (t, 4H, piperazinyl-H), 1.34–1.29 (q, J = 4.21 Hz, 2H, CH_2_-CH-CH_2_ cyclopropyl-H), 1.19–1.13 (q, J = 4.12 Hz, 2H, CH_2_-CH-CH_2_cyclopropyl-H); ^13^C NMR: δ 176.77, 169.23, 166.38, 154.63, 152.16, 148.25, 145.53, 139.63, 135.83, 134.13,, 131.99, 123.66, 123.33, 118.99, 111.47, 111.29, 107.32, 106.19, 59.39, 54.22, 49.46, 36.23, 7.94. C_26_H_23_FN_4_O_5_: C, 63.67; H, 4.73; N, 11.42, Found: C, 63.51; H, 4.64; N, 11.33.

##### 1-Cyclopropyl-7-[4-(2,5-dioxo-pyrrolidin-1-ylmethyl)-piperazin-1-yl]-6-fluoro-4-oxo-1,4-dihydro-quinoline-3-carboxylic acid (**21**)

Yield (88%); m.p. 214 °C; IR: 3412 (OH), 3007 (CH aromatic), 2998, 2987, 2841, 2828, 2810 (CH-aliphatic),1752, 1701 (2 C=O), 1614 (C=N); ^1^H NMR: δ 15.12 (brs. OH, COOH), 8.63–7.51 (m, 3H, aromatic), 4.34 (s, 2H, N-CH_2_-N), 3.83–3.80 (m, J = 2.02 Hz, 1H, CH_2_-CH-CH_2_ cyclopropyl-H), 3.79–3.77 (t, 2H, CO-CH_2_-CH_2_-C=O), 3.49–3.42 (m, 6H, 4H of piperazinyl-H + CO-CH_2_-CH_2_-C=O), 2.72–2.58 (t, 4H, piperazinyl-H), 1.34–1.32 (q, J = 1.48 Hz, 2H, CH_2_-CH-CH_2_ cyclopropyl-H), 1.17–1.08 (q, J = 2.58 Hz, 2H, CH_2_-CH-CH_2_cyclopropyl-H); ^13^C NMR: δ 179.15, 176.88, 166.32, 154.67, 152.28, 148.28, 145.62, 145.52, 139.64, 119.83, 111.49, 111.36, 107.34, 106.19, 59.52, 56.53, 54.25, 49.87, 36.24, 30.54, 18.93, 8.88.: C_22_H_23_FN_4_O_5_: C, 59.72; H, 5.24; N, 12.66, Found: C, 59.59; H, 5.08; N, 12.49.

### 3.2. Antimicrobial Activity

#### 3.2.1. Organisms and Culture Conditions

The cultures used were collected from the Cairo University’s Microanalytical Centre, Faculty of Science. An updated Kirby-Bauer disc diffusion method was applied for antimicrobial activities of the tests **1–21** compounds [32,33,34,35]. See Appendix A.

#### 3.2.2. Minimum Inhibitory Concentration Assay

On 96-well microtiter plates and 50 mL of fresh bacterial culture of a single McFarland unit overnight, a double serial dilution of each compound (100 mL) in sterile standard saline was prepared for every single source well. Ciprofloxacin antibiotic (5 mg/mL^−1^) and normal saline were included as standard references in each assay [36] (see Appendix A).

#### 3.2.3. Inhibitory Activity Assays on *E. coli* DNA Gyrase and Topoisomerase IV

IC_50_ assay determination was carried out in accordance with the procedures previously stated [37] (see Appendix A).

#### 3.2.4. MTT Assay

MTT Assay was performed to investigate the effect of compounds **2–6** on the viability of mammary epithelial cells (MCF-10A) [38,39] (see Appendix A).

## 4. Conclusions

Several novel heteroaryl-based ciprofloxacin derivatives have been developed. Twenty-one target compounds were synthesized and tested for their in vitro antibacterial activity against bacterial strains of both Gram-positive and Gram-negative using ciprofloxacin as a reference. Most of the compounds examined had evident inhibitory antibacterial activity. Among those compounds, **2**–**6** were the most potent ones. The findings showed that the compounds tested displayed little or poor antifungal activity against *A. flavus* and *C. albicans*. Oxadiazole-based derivatives **4**, **5**, and **6** were found to be the most active and their inhibitory activity against *E. coli* DNA gyrase and Topoisomerase IV was superior to novobiocin with no cytotoxic effects. These compounds, after further optimization, form a new class of antibacterial molecules that target DNA gyrase and topoisomerase IV.

## Figures and Tables

**Table 1:** Inhibitory zone diameter (mm) of compounds **1–21**.

Compound	Inhibition Zone Diameter (mm/mg Sample)					
	Bacterial Species				Fungi	
	(G^+^)		(G^−^)			
	*B. subtilis*	*S. aureus*	*E. coli*	*P. aeruginosa*	*A. flavus*	*C. albicans*
**Ciprofloxacin**	**40**	**40**	**40**	**40**	--	--
**Fluconazole**	--	--	--	--	**40**	**40**
**1**	31	36	34	31	0.0	0.0
**2**	36	37	36	36	0.0	9
**3**	36	37	37	35	0.0	13
**4**	37	41	41	40	0.0	10
**5**	37	41	41	38	0.0	10
**6**	38	48	38	37	0.0	10
**7**	32	35	32	32	0.0	0.0
**8**	32	32	33	31	0.0	0.0
**9**	31	32	30	31	0.0	0.0
**10**	30	34	31	32	0.0	0.0
**11**	35	31	31	35	0.0	0.0
**12**	35	33	35	35	0.0	0.0
**13**	0.0	0.0	0.0	0.0	0.0	0.0
**16**	37	34	37	37	0.0	11
**17**	32	28	31	31	0.0	0.0
**18**	32	31	32	33	0.0	0.0
**19**	32	31	31	31	0.0	0.0
**20**	33	41	35	33	0.0	0.0
**21**	31	36	28	31	0.0	0.0

**Table 2:**
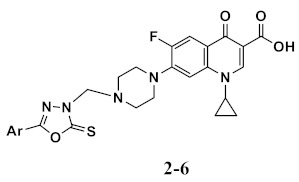
Antimicrobial activities of compounds **2–6**.

Minimum Inhibitory Concentration (MIC) in µg/mL					
**Compound**	Ar	Bacterial Species			
		(G^+^)		(G^−^)	
		*B. subtilis*	*S. aureus*	*E. coli*	*P. aeruginosa*
**2**	**2-Cl-Ph**	0.125	0.162	0.125	0.125
**3**	**2-Naphthyl**	0.125	0.125	0.125	0.05
**4**	**2-Pyridyl**	0.125	0.035	0.062	0.062
**5**	**3-Pyridyl**	0.125	0.035	0.062	0.125
**6**	**4-Pyridyl**	0.125	0.031	0.125	0.125
**Ciprofloxacin**	**--**	0.010	0.030	0.060	0.060

**Table 3:** *E. coli* DNA gyrase and topoisomerase IV inhibitory activities of compounds **2**–**6** and Novobiocin.

Compound	IC_50_ (nM) ^a^ or RA (%) ^b^	IC_50_ (µM) ^a^ or RA (%) ^b^
	*E. coli* DNA Gyrase	*E. coli* Topo IV
**2**	57%	89%
**3**	86 ± 9 nM	58%
**4**	42 ± 7 nM	1.47 ± 0.20 µM
**5**	92 ± 9 nM	6.80 ± 0.20 µM
**6**	180 ± 20 nM	8.92 ± 0.20 µM
**Novobiocin**	170 ± 20 nM	11 ± 2 µM

^a^ Concentration of compound that inhibits the enzyme activity by 50%; ^b^ residual activity of the enzyme at 1 µM of the compound.

**Table 4:** Cell viability assay results of compounds **2–6**.

Compound	Cell Viability %
2	87
3	85
4	89
5	91
6	87

## Data Availability

Not applicable.

## Supplementary Materials

The following are available online at https://www.mdpi.com/article/10.3390/ph14050399/s1, Figure S1–S21: ^1^H, ^13^C NMR and Dept 135 of compounds **1**–**20**, Table S1–S3: Molecular properties of compounds **1**–**21**.

## Appendix A

### Appendix A.1. General Details

All the commercially available reagent chemicals were obtained from Sigma-Aldrich Corporation (St. Louis, MO, USA) and were used without further purification. All reactions were monitored by thin layer chromatography (TLC) using precoated plates of silica gel G/UV-254 of 0.25 mm thickness (Merck 60F254-Billerica, MA, USA) using UV light (254 nm/365 nm) for visualization. Melting points were detected with a Koffler melting points apparatus and were uncorrected. Infrared spectra were recorded with a FT-IR-ALPHBROKER-Platinum-ATR spectrometer and are given as cm^−1^ using the attenuated total reflection (ATR) method. ^1^H NMR and ^13^C NMR spectra for all compounds were recorded in DMSO-*d_6_* on a Bruker Bio Spin AG spectrometer at 400 MHz and 100 MHz, respectively. For ^1^H NMR, chemical shifts (δ) were given in parts per million (ppm) with reference to Tetramethylsilane (TMS) as an internal standard (δ = 0); coupling constants (J) were given in hertz (Hz) and data are reported as follows: chemical shift, integration, multiplicity (s = singlet, d = doublet, t = triplet, q = quartet, m = multiplet, dd = doublet of doublets). For ^13^C NMR, TMS (δ = 0) or DMSO (δ = 39.51) was used as internal standard and spectra were obtained with complete proton decoupling. Elemental analyses were obtained on a Perkin-Elmer CHN-analyzer model. Microwave irradiations were carried out in a Kenstar OM9925E MW oven (2450 MHz, 800 W).

### Appendix A.2. Antimicrobial Activity

#### Appendix A.2.1. Organisms and Culture Conditions

The cultures used were collected from the Cairo University’s Microanalytical Centre, Faculty of Science. An updated Kirby-Bauer disc diffusion method was applied for the antimicrobial activities of the tests for compounds **1–21** [28]. Shortly after, 10 mL of fresh medium was grown to 100 μL bacteria/food until a count of 108 cells/mL or 105 cells/mL was achieved [29]. One hundred μL microbial suspension was spread over agar plates that suited the broth in which it was held. The selected colonies of each organism that may play a pathogenic function should be from the primary agar plates and tested by the disc diffusion method for susceptibility [30,31]. Plates inoculated with filamentous fungi as *Aspergillus flavus* were incubated at 25 °C for 48 h; Gram positive bacteria as *Staphylococcus aureus* (ATCC 12600, Microanalytical center, Cairo, Egypt), *Bacillus subtilis* (ATCC 6051); Gram negative bacteria as *Escherichia coli* (ATCC 11775), *Pseudomonas aeuroginosa* (ATCC 10145) were incubated at 35–37 °C for 24–48 h and yeast as *Candida albicans* (ATCC 7102) was incubated at 30 °C for 24–48 h and, then the diameters of the inhibition zones were measured in millimeters [28]. Standard discs of ciprofloxacin (antibacterial agent), fluconazole (antifungal agent) served as positive controls for antimicrobial activity, but filter discs impregnated with 10 µL of solvent (distilled water, chloroform, DMSO) were used as a negative control. Blank paper disks (Schleicher and Schuell, Cytiva, Spain) with a diameter of 8.0 mm were impregnated with 10 µL of tested concentration of the stock solutions. When a filter paper disc impregnated with a tested chemical is placed on agar, the chemical will diffuse from the disc into the agar. This diffusion will place the chemical in the agar only around the disc. The solubility of the chemical and its molecular size will determine the size of the area of chemical infiltration around the disc. If an organism is placed on the agar, it will not grow in the area around the disc if it is susceptible to the chemical. This area of no growth around the disc is known as a “Zone of inhibition” or" Clear zone". For the disc diffusion, the zone diameters were measured with slipping calipers of the National Committee for Clinical Laboratory Standards [30], and the results are given in Table 1. Agar-based methods such as E-test and disk diffusion can be good alternatives because they are simpler and faster than broth-based methods [30,31].

#### Appendix A.2.2. Minimum Inhibitory Concentration Assay

In 96-well microtiter plates and 50 mL of fresh bacterial culture of a single McFarland unit overnight, a double serial dilution of each compound (100 mL) in sterile standard saline was prepared for every single source well. Ciprofloxacin antibiotic (5 mg/mL^−1^) and normal saline were included as standard reference in each assay [32]. The plates were incubated at 37 °C overnight. As an indicator of bacterial growth, 40 mL of *p*-iodonitrotetrazolium violet (INT) was added to each well and incubated at 37 °C for 30 min. MIC values are recorded as the lowest concentration of the extract that completely inhibited bacterial growth that is clear well. The colorless tetrazolidium salt acts as an electron accepter and is reduced to a red colored formazan product by biological activity organisms. Where bacterial growth was inhibited, the solution in the well remained clear after incubation with INT. The observed MIC values are presented in Table 2.

#### Appendix A.2.3. Determination of Inhibitory Activities on *E. coli* and *S. aureus* DNA Gyrase

Inhibitory activities were determined in an assay from Inspiralis on streptavidin-coated 96-well microtiter plates from Thermo scientific Pierce. First, the plates were rehydrated with a buffer (20 mM TrisHCl with pH 7.6, 0.01% *w/v* BSA, 0.05% *v/v* Tween 20, 137 mM NaCl) and the biotinylated oligonucleotide was then immobilized. After washing off the unbound oligonucleotide, the enzyme test was performed. The reaction volume of 30 μL in buffer (35 mM Tris × HCl with pH 7.5, 4 mM MgCl_2_, 24 mM KCl, 2 mM DTT, 1.8 mM spermidine, 1 mM ATP, 6.5 % *w/v* glycerol, 0.1 mg/mL albumin) contained 1.5 U of DNA gyrase from *E. coli* or *S. aureus*, 0.75 μg of relaxed pNO1 plasmid, and 3 μL solution of the inhibitor in 10% DMSO and 0.008% Tween 20. Reaction solutions were incubated at 37 °C for 30 min. After that, the TF buffer (50 mM NaOAc with pH 5.0, 50 mM NaCl and 50 mM MgCl_2_) was added to terminate the enzymatic reaction. After additional incubation for 30 min at rt, during which biotin-oligonucleotide-plasmid triplex was 19 formed, the unbound plasmid was washed off using TF buffer and SybrGOLD in T10 buffer (10 mM Tris HCl with pH 8.0 and 1 mM EDTA) was added. The fluorescence was measured with a microplate reader (BioTek Synergy H4, excitation: 485 nm, emission: 535 nm). Initial screening was done at 100 or 10 μM concentration of inhibitors. For the most active inhibitors, IC50 was determined using seven concentrations of tested compounds. GraphPad Prism software was used to calculate the IC50 values. The result is given as the average value of three independent measurements. As the internal standard, novobiocin (IC_50_ = 0.168 µM for *E. coli* gyrase and IC_50_ = 0.041 µM for *S. aureus* gyrase) was used. Determination of inhibitory activities on *E. coli* and *S. aureus* Topoisomerase IV IC_50_ values were determined in an assay from Inspiralis on streptavidin-coated 96-well microtiter plates from Thermo scientific Pierce. First, the plates were rehydrated with buffer (20 µM Tris-HCl with pH 7.6, 0.01% *w/v* BSA, 0.05% *v/v* Tween 20, 137 mM NaCl) and biotinylated oligonucleotide was then immobilized. After washing off the unbound oligonucleotide, the enzyme test was performed. The reaction volume of 30 μL in buffer (40 mM HEPES KOH with pH 7.6, 100 mM potassium glutamate, 10 mM magnesium acetate, 10 mM DTT, 1 mM ATP, 0.05 mg/mL albumin) contained 1.5 U of topoisomerase IV from *E. coli* or *S. aureus*, 0.75 μg of pNO1 supercoiled plasmid, and 3 μL solution of the inhibitor in DMSO (10%) and Tween 20 (0.008%). Reaction mixtures were incubated at 37 °C for 30 min and after that, the TF buffer (50 mM NaOAc with pH 5.0, 50 mM NaCl and 50 mM MgCl_2_) was added to terminate the enzymatic reaction. After additional incubation for 30 min at rt, during which triplex (biotin-oligonucleotide-plasmid) was formed, the unbound plasmid was washed off using TF buffer and SybrGOLD in T10 buffer (10 mM Tris HCl with pH 8.0 and 1 mM EDTA) was added. The fluorescence was measured with a microplate reader (BioTek Synergy H4, excitation: 485 nm, emission: 535 nm). Initial screening was done at 100 or 10 μM concentration of inhibitors. For the most active inhibitors IC50 was determined using seven concentrations of tested compounds. GraphPad Prism software was used to calculate the IC50 values. The result is given as the average value of three independent measurements. As the internal standard, novobiocin (IC_50_ = 11.1 µM) for *E. coli* topoisomerase IV and IC_50_ = 26.7 µM for *S. aureus* topoisomerase IV) was used.
