# Supplementary material for: Design, Synthesis, and Antibacterial Screening of Some Novel Heteroaryl-Based Ciprofloxacin Derivatives as DNA Gyrase and Topoisomerase IV Inhibitors
Source: Pharmaceuticals (Basel). 2021 Apr 22;14(5):399. doi: 10.3390/ph14050399 (PMC8145110; doi:10.3390/ph14050399)
Supplement: Supplementary file 1 [file pharmaceuticals-14-00399-s001.zip › pharmaceuticals-1120557-supplementary.pdf]

Supporting Information

Figure S1- ^{1}H, ^{13}C NMR and dept-135 Spectra of 1:

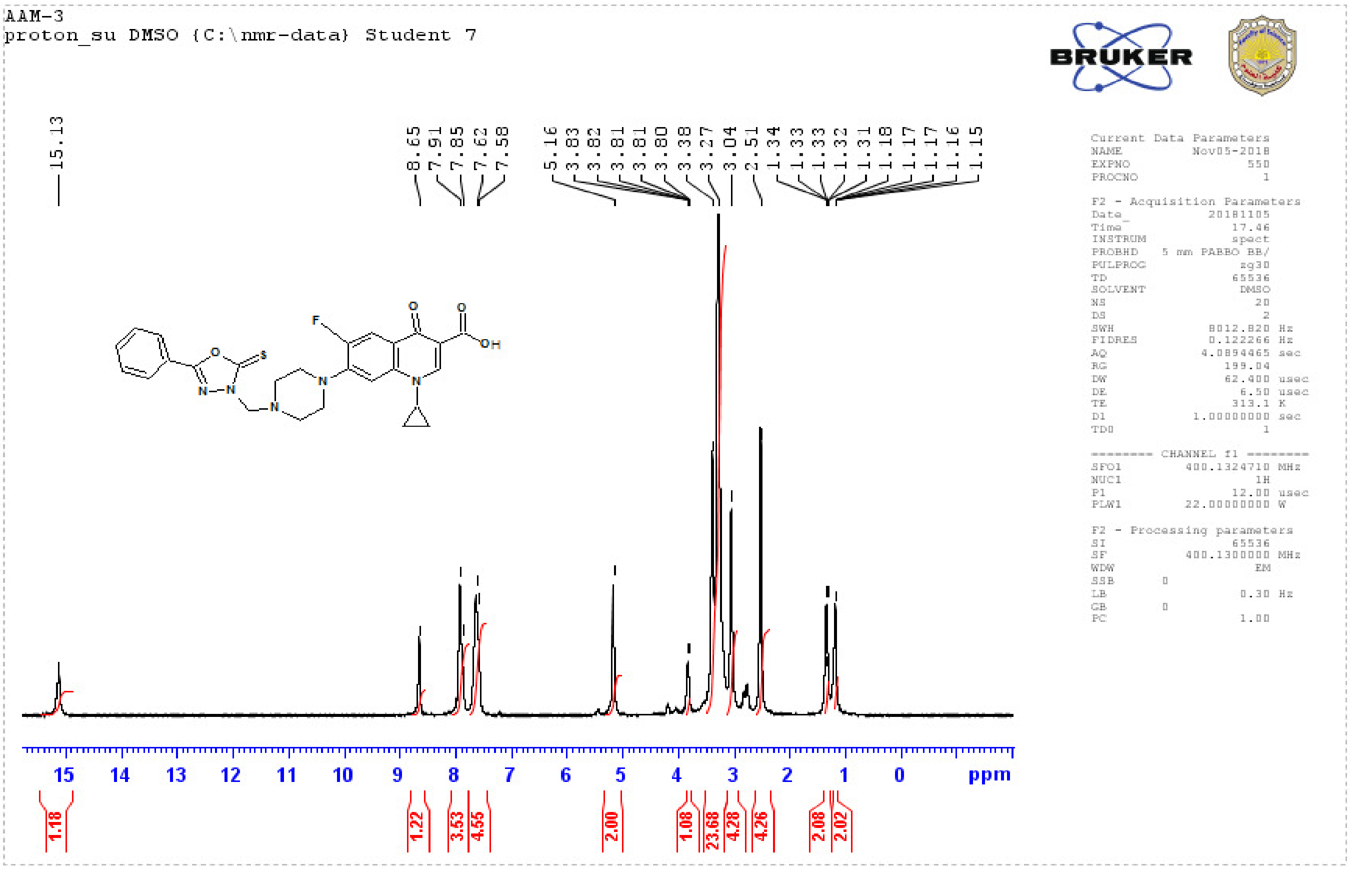

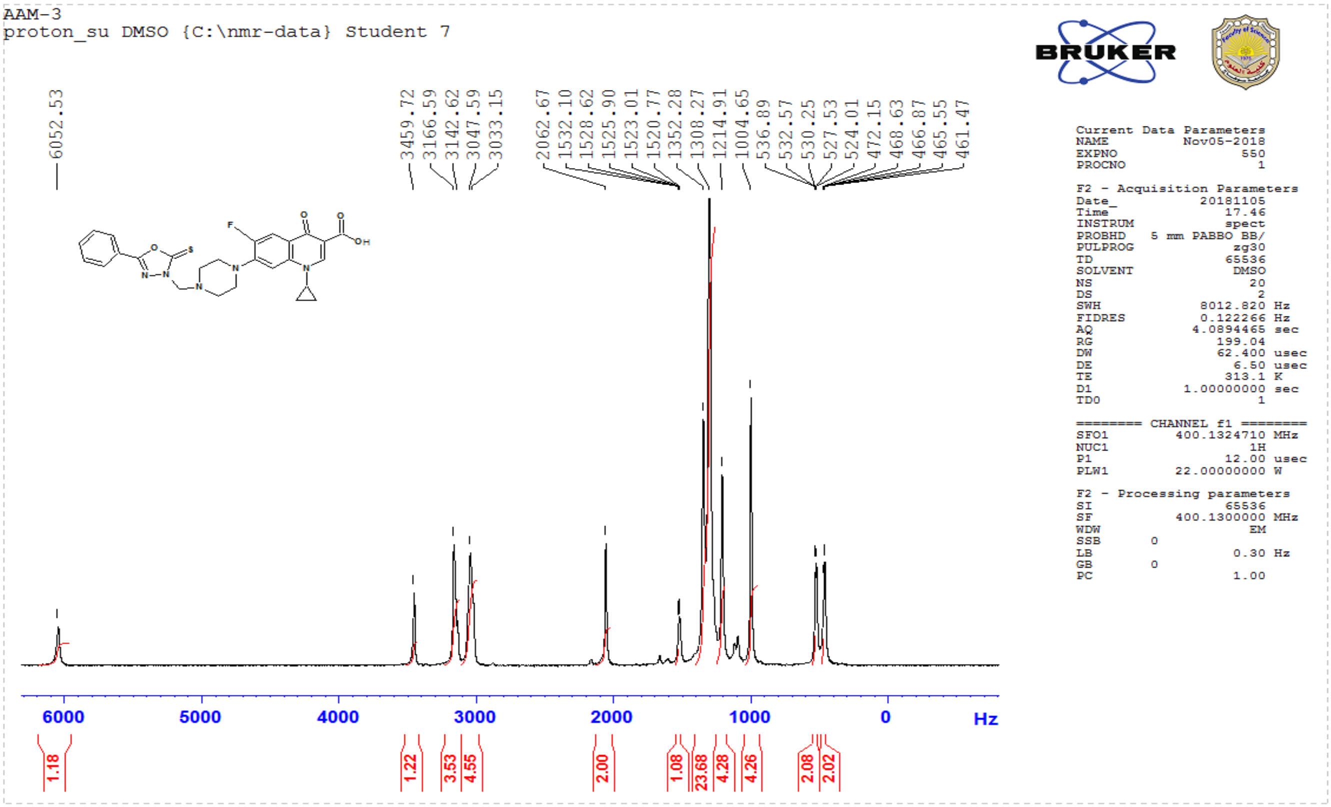
AAM-3
proton_su DMSO {C:\nmr-data} Student 7
6052.53
3459.72
3166.59
3142.62
3047.59
3033.15
2062.67
1532.10
1528.62
1525.90
1523.01
1520.77
1352.28
1308.27
1214.91
1004.65
536.89
532.57
530.25
527.53
524.01
472.15
468.63
466.87
465.55
461.47
F
O
OH
N
S
Current Data Parameters
NAME Nov05-2018
EXPNO 550
PROCNO 1
F2 - Acquisition Parameters
Date_ 20181105
Time 17.46
INSTRUM spect
PROBHD 5 mm PABBO BB/
PULPROG zg30
TD 65536
SOLVENT DMSO
NS 20
DS 2
SWH 8012.820 Hz
FIDRES 0.122266 Hz
AQ 4.0894465 sec
RG 199.04
DW 62.400 usec
DE 6.50 usec
TE 313.1 K
D1 1.00000000 sec
TD0 1
======== CHANNEL f1 ========
SFO1 400.1324710 MHz
NUC1 1H
P1 12.00 usec
PLW1 22.00000000 W
F2 - Processing parameters
SI 65536
SF 400.1300000 MHz
WDW EM
SSB 0
LB 0.30 Hz
GB 0
PC 1.00
6000
5000
4000
3000
2000
1000
0
Hz
1.18
1.22
3.53
4.55
2.00
1.08
23.68
4.28
4.26
2.08
2.02

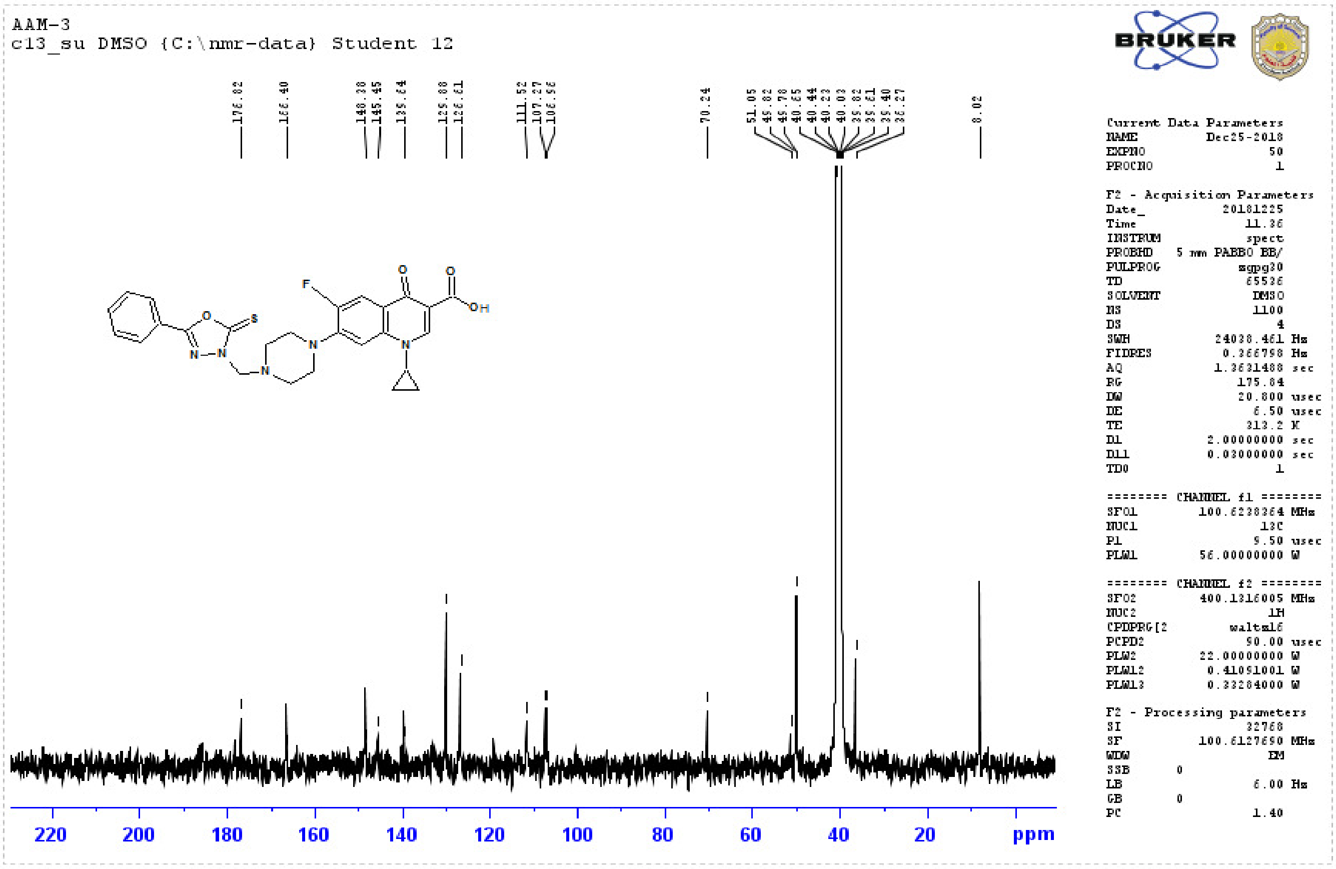
AAM-3
c13_su DMSO {C:\nmr-data} Student 12
176.82
166.40
148.38
145.45
139.64
129.88
126.61
111.52
107.27
106.56
70.24
51.05
49.82
49.78
40.65
40.44
40.23
40.03
39.82
39.61
39.40
39.27
8.02
F
O
OH
N
S
Current Data Parameters
NAME Dec25-2018
EXPNO 50
PROCNO 1
F2 - Acquisition Parameters
Date_ 20181225
Time 11.36
INSTRUM spect
PROBHD 5 mm PABBO BB/
PULPROG zgpg30
TD 65536
SOLVENT DMSO
NS 1100
DS 4
SWH 24038.461 Hz
FIDRES 0.366798 Hz
AQ 1.3631488 sec
RG 175.84
DW 20.800 usec
DE 6.50 usec
TE 313.2 K
D1 2.00000000 sec
D11 0.03000000 sec
TD0 1
======== CHANNEL f1 ========
SFO1 100.6238364 MHz
NUC1 13C
P1 9.50 usec
PLW1 56.00000000 W
======== CHANNEL f2 ========
SFO2 400.1316005 MHz
NUC2 1H
CPDPRG[2 waltz16
PCPD2 90.00 usec
PLW2 22.00000000 W
PLW12 0.41051001 W
PLW13 0.33284000 W
F2 - Processing parameters
SI 32768
SF 100.6127690 MHz
WDW EM
SSB 0
LB 6.00 Hz
GB 0
PC 1.40
220
200
180
160
140
120
100
80
60
40
20
ppm

S2- ^{1}H, ^{13}C NMR and dept-135 Spectra of 2

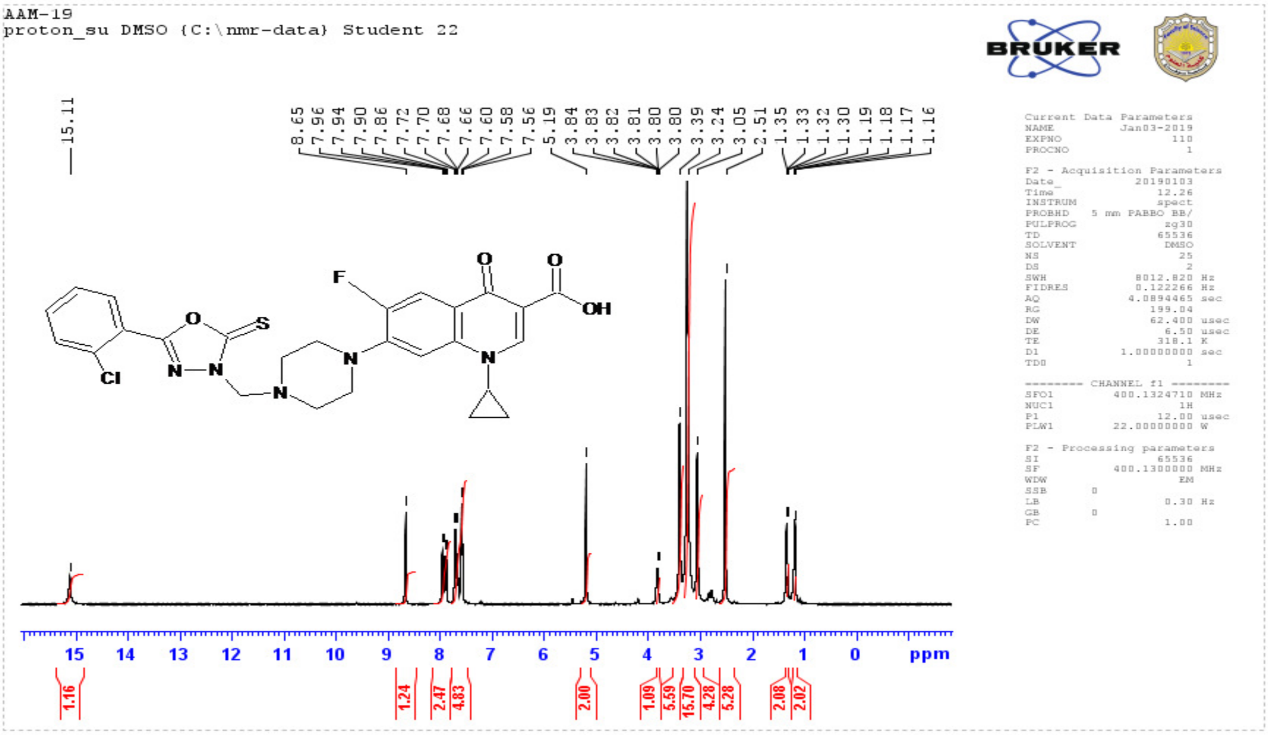

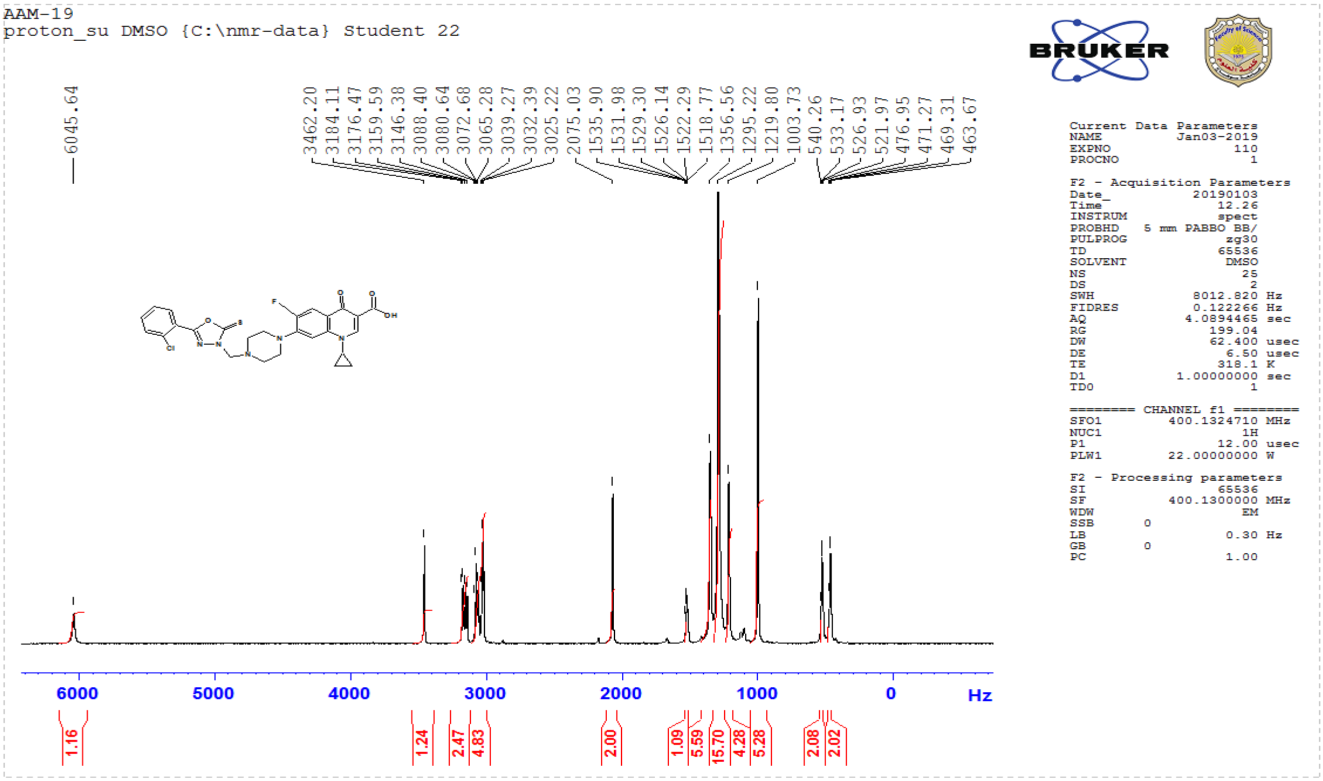

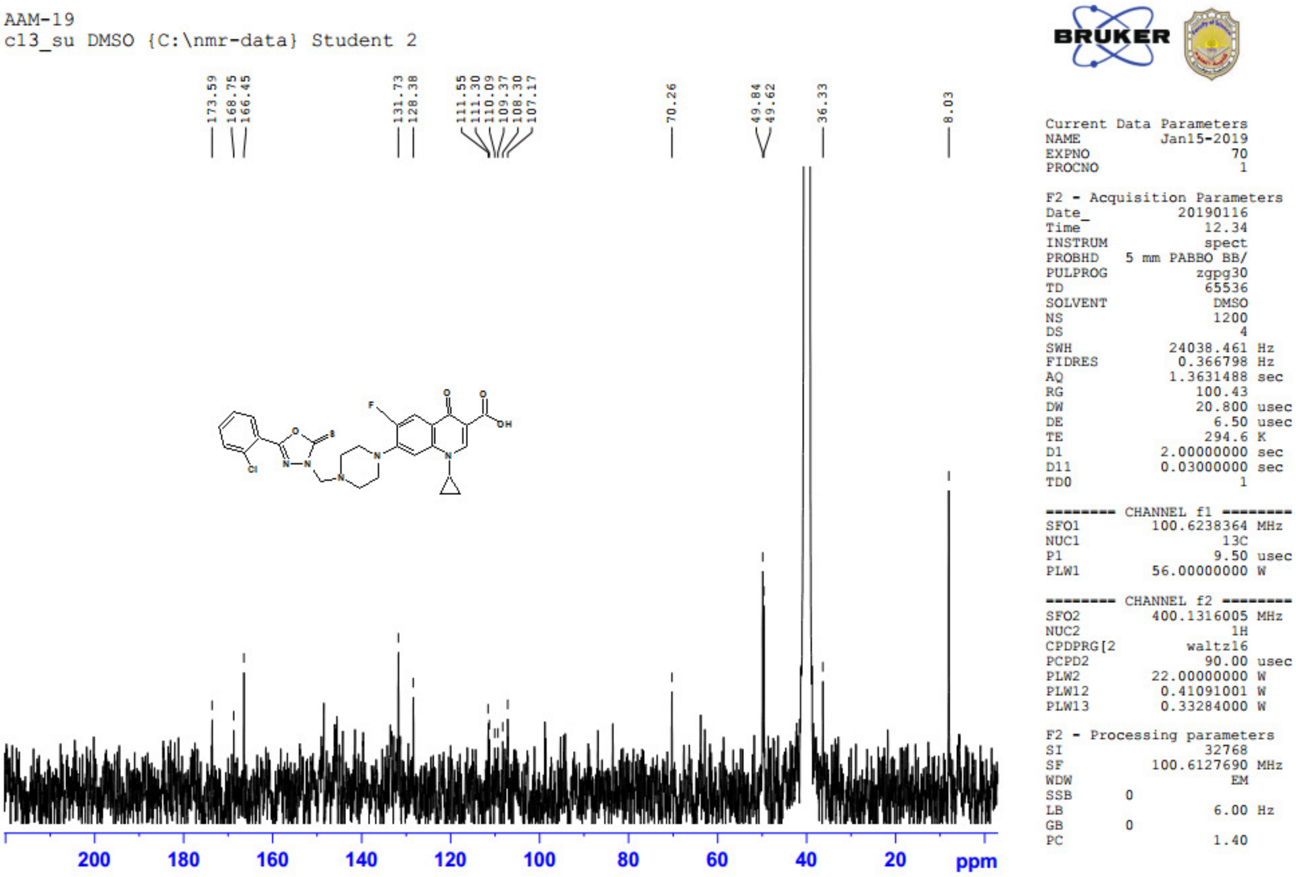

S3-^{1}H, ^{13}C NMR and dept-135 Spectra of 3

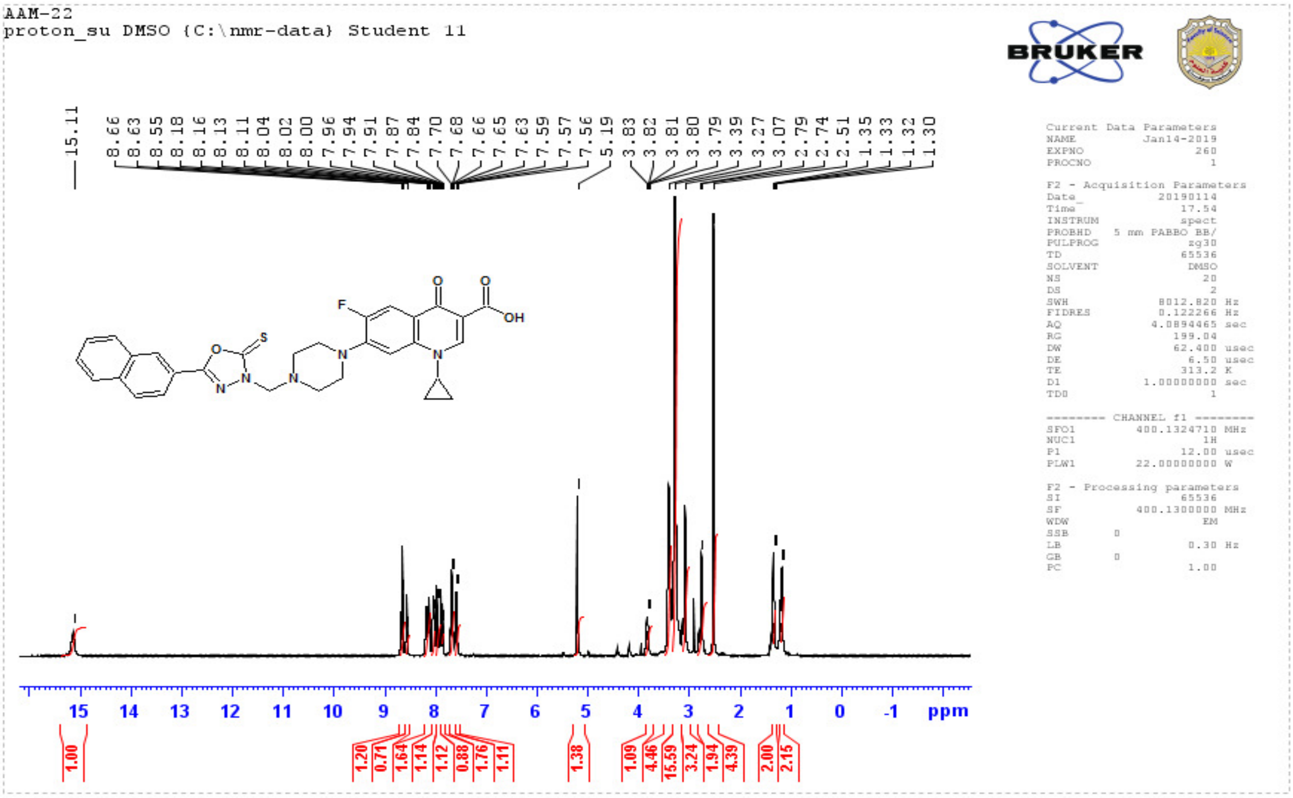

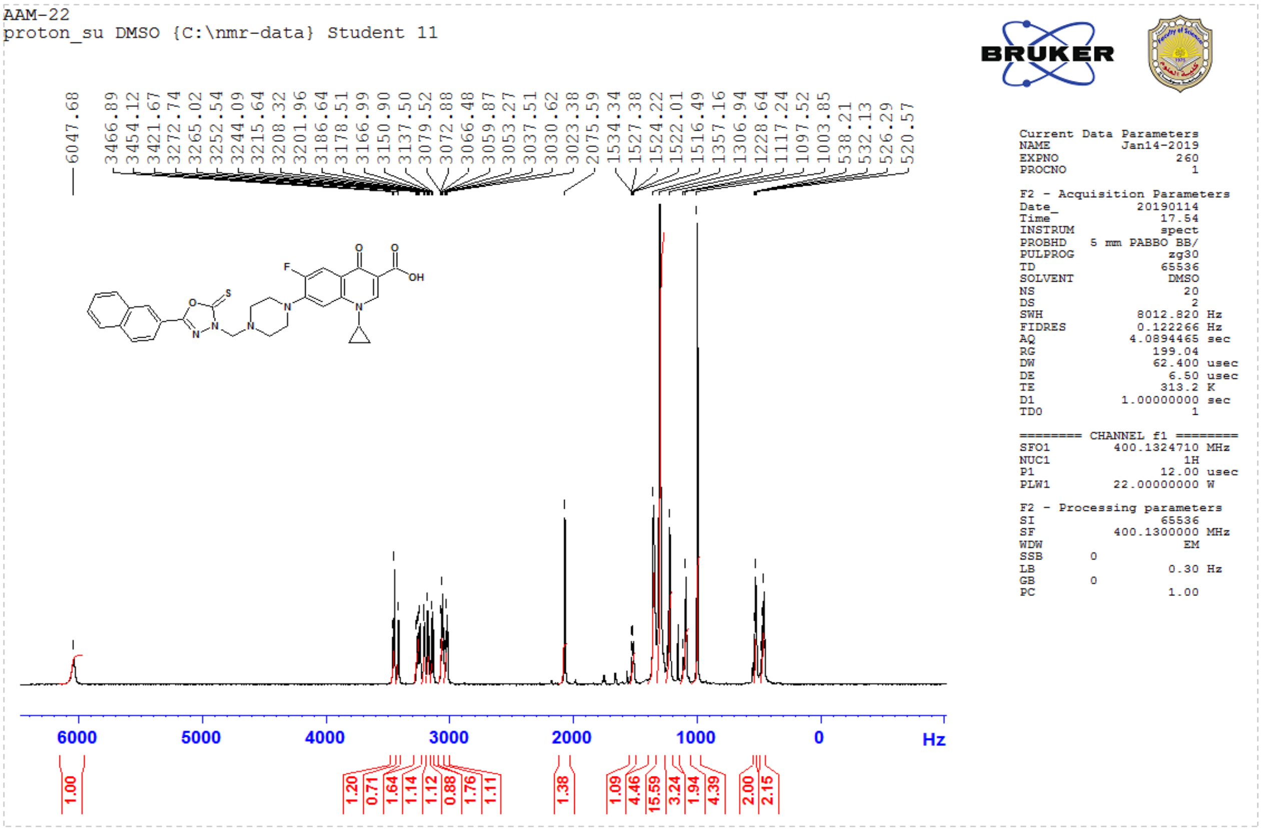
AAM-22
proton_su DMSO {C:\nmr-data} Student 11
BRUKER
Current Data Parameters
NAME Jan14-2019
EXPNO 260
PROCNO 1
F2 - Acquisition Parameters
Date_ 20190114
Time 17.54
INSTRUM spect
PROBHD 5 mm PABBO BB/
PULPROG zg30
TD 65536
SOLVENT DMSO
NS 20
DS 2
SWH 8012.820 Hz
FIDRES 0.122266 Hz
AQ 4.0894465 sec
RG 199.04
DW 62.400 usec
DE 6.50 usec
TE 313.2 K
D1 1.00000000 sec
TD0 1
======== CHANNEL f1 ========
SFO1 400.1324710 MHz
NUC1 1H
P1 12.00 usec
PLW1 22.00000000 W
F2 - Processing parameters
SI 65536
SF 400.1300000 MHz
WDW EM
SSB 0
LB 0.30 Hz
GB 0
PC 1.00
Hz
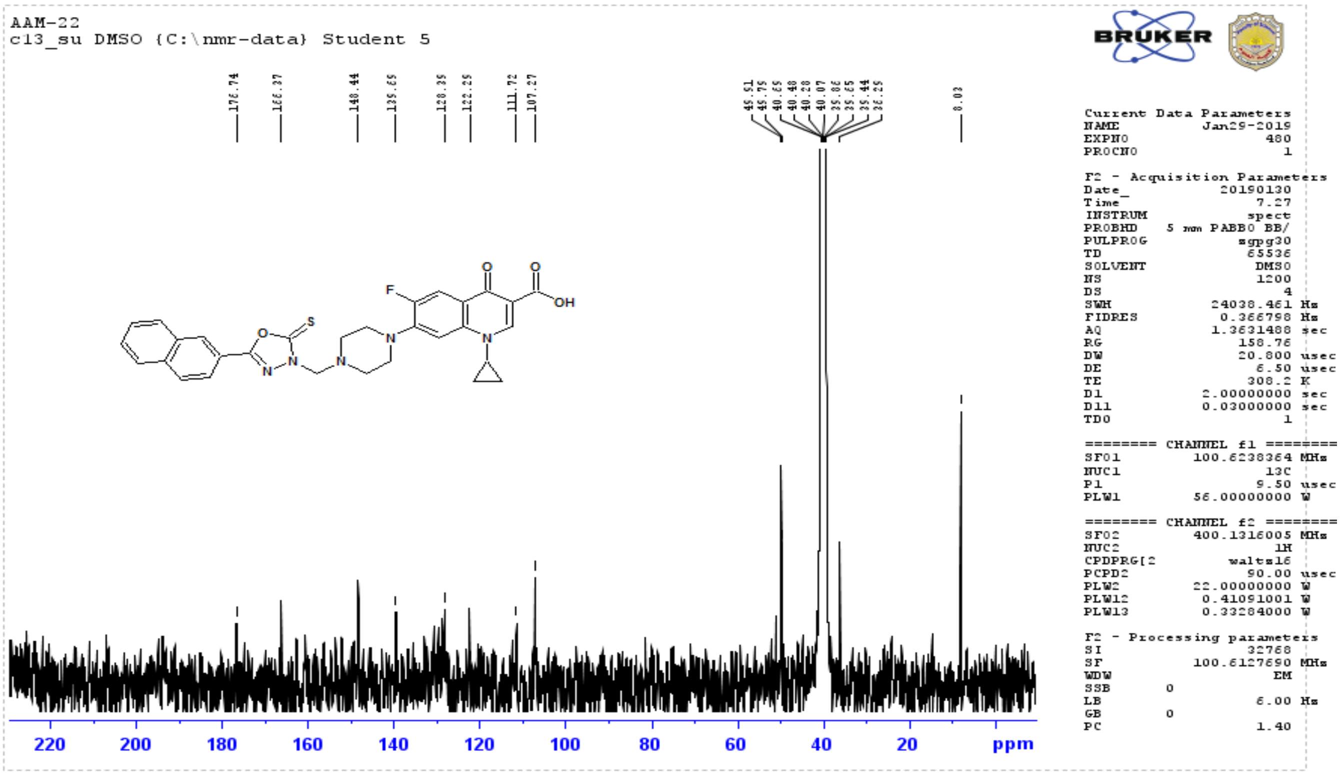
AAM-22
c13_su DMSO {C:\nmr-data} Student 5
BRUKER
Current Data Parameters
NAME Jan29-2019
EXPNO 480
PROCNO 1
F2 - Acquisition Parameters
Date_ 20190130
Time 7.27
INSTRUM spect
PROBHD 5 mm PABBO BB/
PULPROG zgpg30
TD 65536
SOLVENT DMSO
NS 1200
DS 4
SWH 24038.461 Hz
FIDRES 0.366798 Hz
AQ 1.3631488 sec
RG 158.76
DW 20.800 usec
DE 6.50 usec
TE 308.2 K
D1 2.00000000 sec
D11 0.03000000 sec
TD0 1
======== CHANNEL f1 ========
SFO1 100.6238364 MHz
NUC1 13C
P1 9.50 usec
PLW1 56.00000000 W
======== CHANNEL f2 ========
SFO2 400.1316005 MHz
NUC2 1H
CPDPRG[2 waltz16
PCPD2 90.00 usec
PLW2 22.00000000 W
PLW12 0.41091001 W
PLW13 0.33284000 W
F2 - Processing parameters
SI 32768
SF 100.6127690 MHz
WDW EM
SSB 0
LB 6.00 Hz
GB 0
PC 1.40
ppm

S4- ^{1}H , ^{13}C NMR and dept-135 Spectra of 4

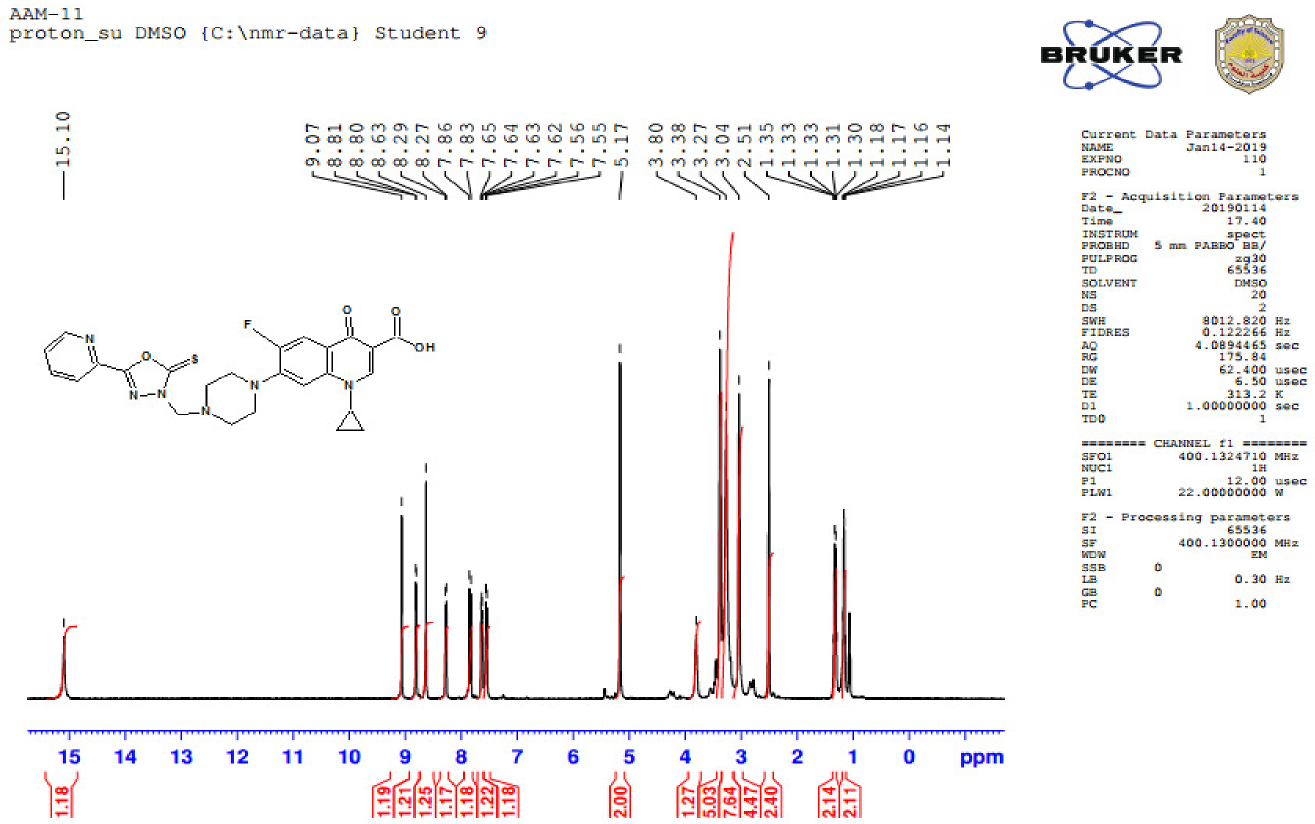

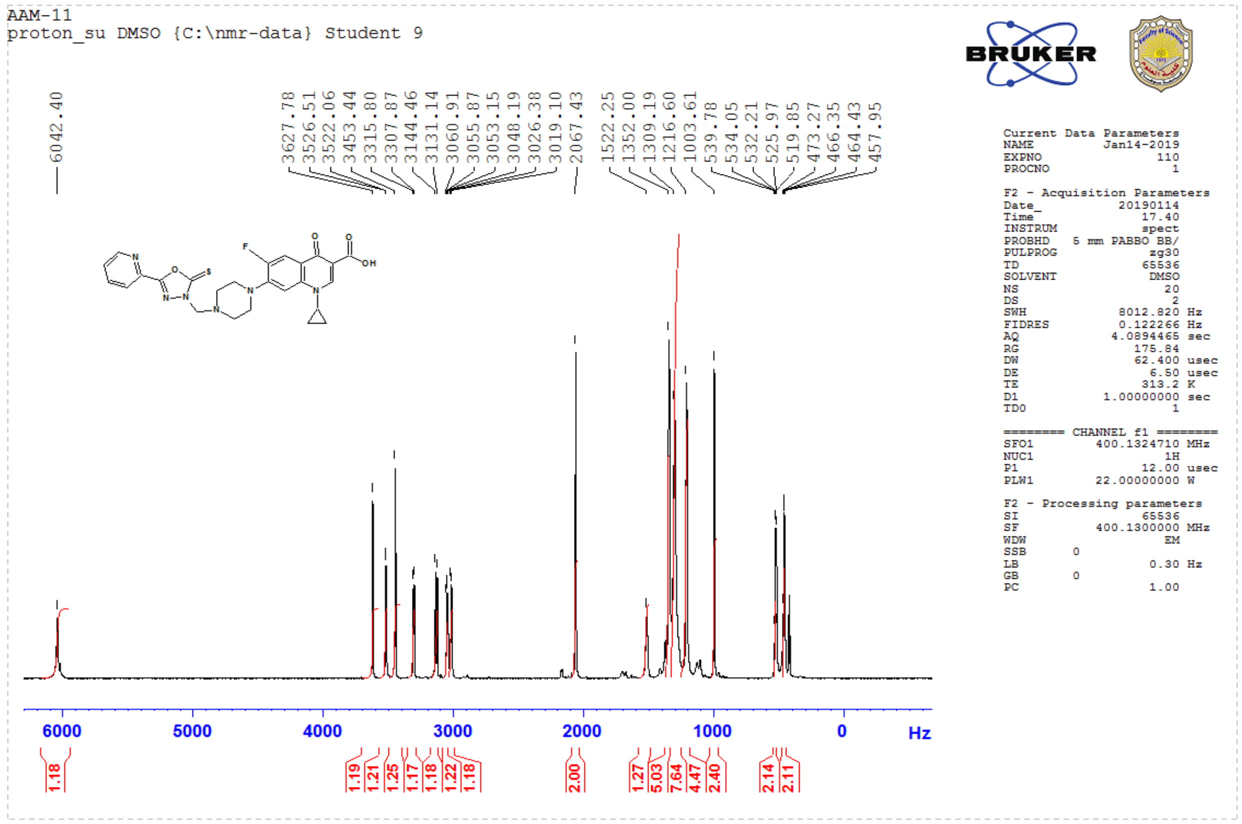

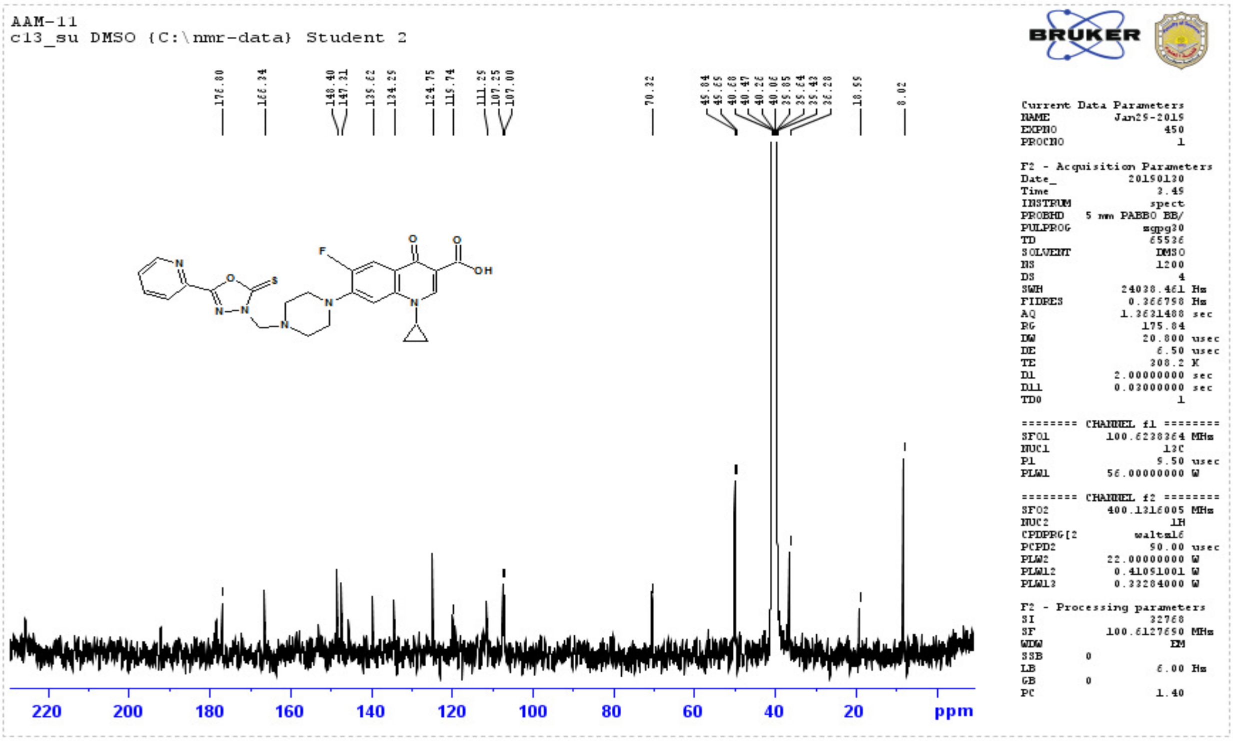

S5- ^{1}H, ^{13}C NMR and dept-135 Spectra of 5

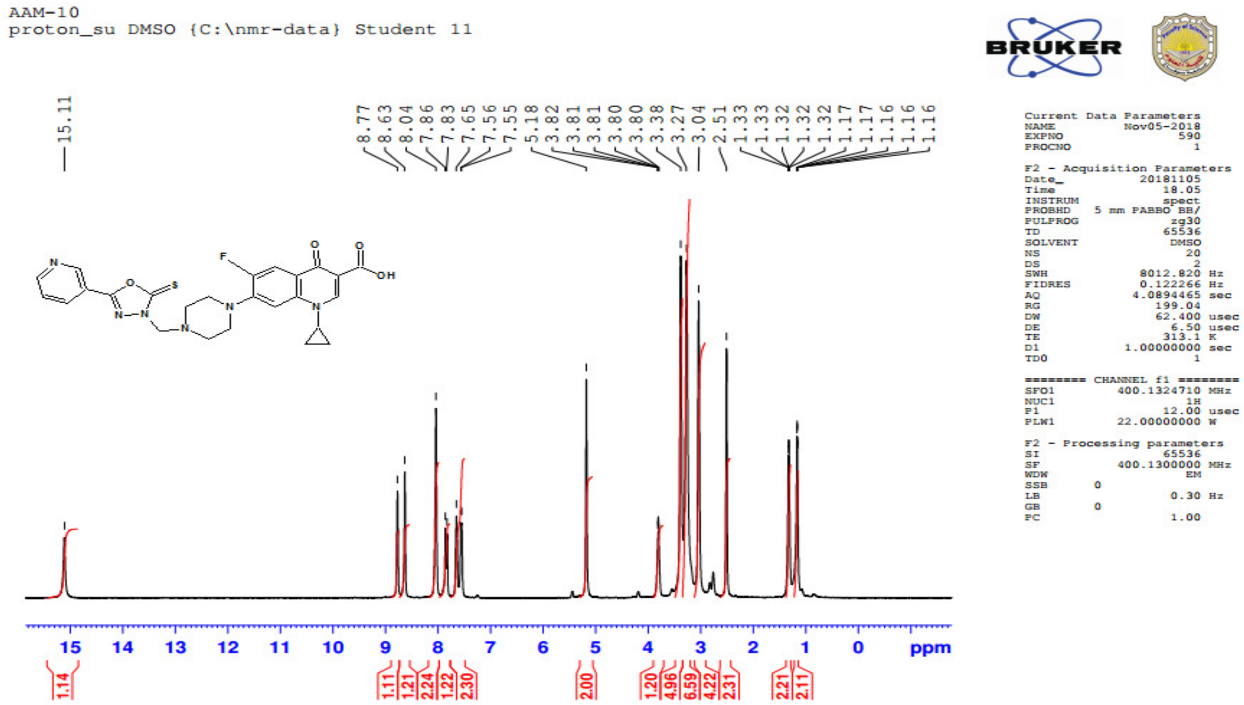

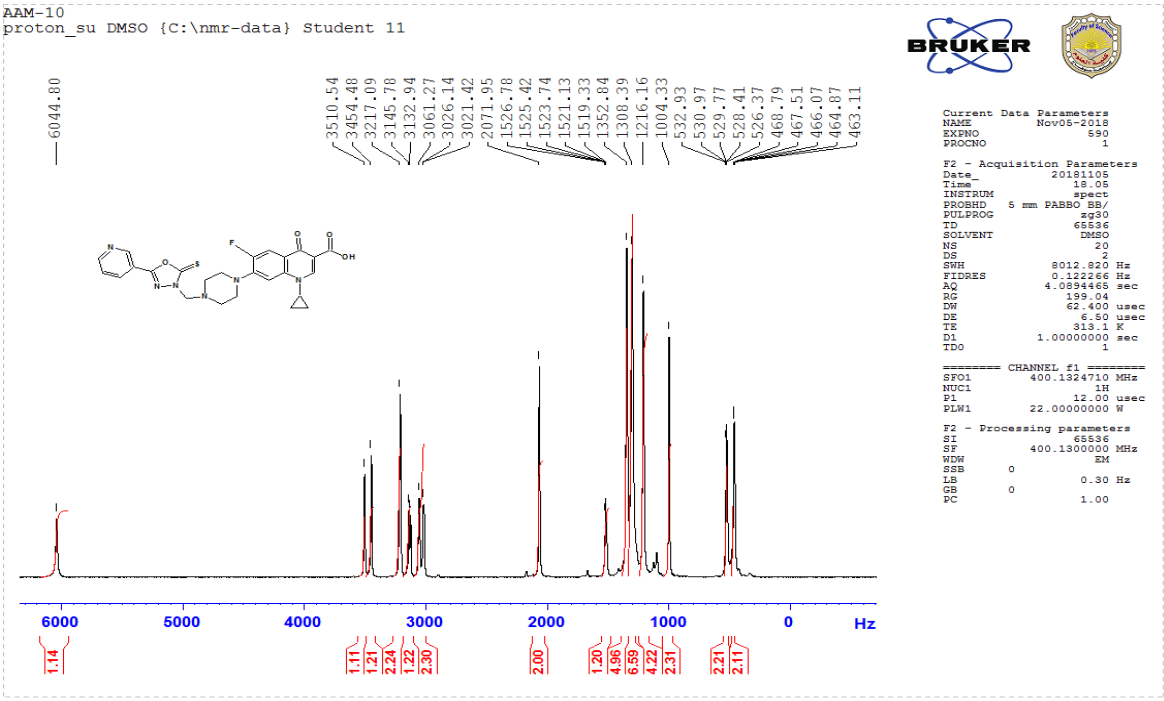
AAM-10
proton_su DMSO {C:\nmr-data} Student 11
BRUKER
6044.80
3510.54
3454.48
3217.09
3145.78
3132.94
3061.27
3026.14
3021.42
2071.95
1526.78
1525.42
1523.74
1521.13
1519.33
1352.84
1308.39
1216.16
1004.33
532.93
530.97
529.77
528.41
526.37
468.79
467.51
466.07
464.87
463.11
Current Data Parameters
NAME Nov05-2018
EXPNO 590
PROCNO 1
F2 - Acquisition Parameters
Date_ 20181105
Time 18.05
INSTRUM spect
PROBHD 5 mm PABBO BB/
PULPROG zg30
TD 65536
SOLVENT DMSO
NS 20
DS 2
SWH 8012.820 Hz
FIDRES 0.122266 Hz
AQ 4.0894465 sec
RG 199.04
DW 62.400 usec
DE 6.50 usec
TE 313.1 K
D1 1.00000000 sec
TD0 1
======== CHANNEL f1 ========
SFO1 400.1324710 MHz
NUC1 1H
P1 12.00 usec
PLW1 22.00000000 W
F2 - Processing parameters
SI 65536
SF 400.1300000 MHz
WDW EM
SSB 0
LB 0.30 Hz
GB 0
PC 1.00
6000
5000
4000
3000
2000
1000
0
Hz
1.14
1.11
1.21
2.24
1.22
2.30
2.00
1.20
4.96
6.59
4.22
2.31
2.21
2.11

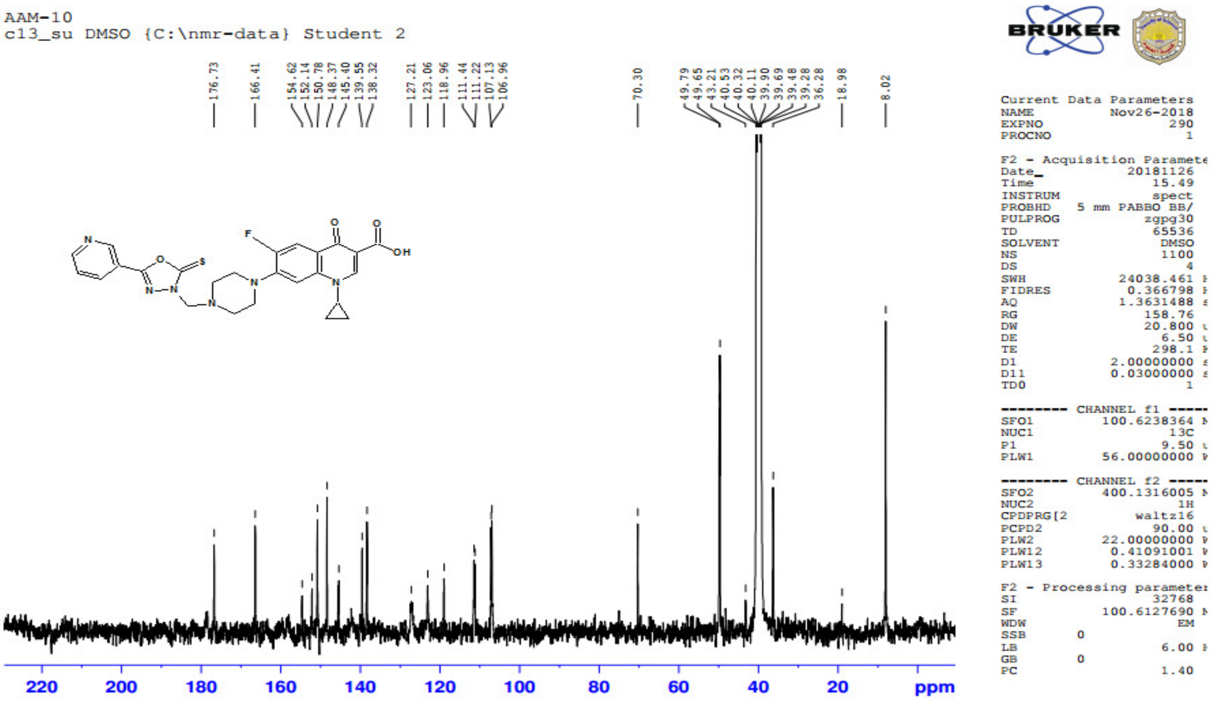
AAM-10
c13_su DMSO {C:\nmr-data} Student 2
BRUKER
176.73
166.41
154.62
152.14
150.78
148.37
145.40
139.55
138.32
127.21
123.06
118.96
111.44
111.22
107.13
106.96
70.30
49.79
49.65
43.21
40.53
40.32
40.11
39.90
39.69
39.48
39.28
36.28
18.98
8.02
Current Data Parameters
NAME Nov26-2018
EXPNO 290
PROCNO 1
F2 - Acquisition Paramete
Date_ 20181126
Time 15.49
INSTRUM spect
PROBHD 5 mm PABBO BB/
PULPROG zgpg30
TD 65536
SOLVENT DMSO
NS 1100
DS 4
SWH 24038.461
FIDRES 0.366798
AQ 1.3631488
RG 158.76
DW 20.800
DE 6.50
TE 298.1
D1 2.00000000
D11 0.03000000
TD0 1
-------- CHANNEL f1 -----
SFO1 100.6238364
NUC1 13C
P1 9.50
PLW1 56.00000000
-------- CHANNEL f2 -----
SFO2 400.1316005
NUC2 1H
CPDPRG[2 waltz16
PCPD2 90.00
PLW2 22.00000000
PLW12 0.41091001
PLW13 0.33284000
F2 - Processing parameter
SI 32768
SF 100.6127690
WDW EM
SSB 0
LB 6.00
GB 0
PC 1.40
220
200
180
160
140
120
100
80
60
40
20
ppm

S6- ^{1}H, ^{13}C NMR and dept-135 Spectra of 6

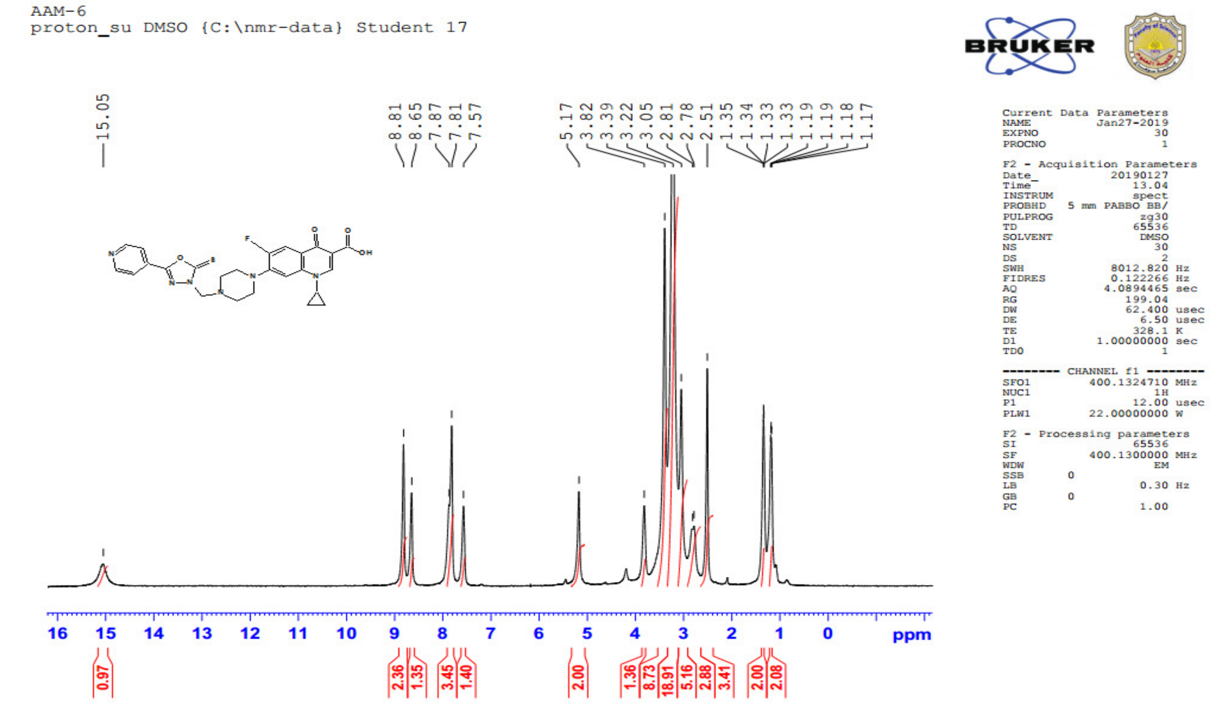

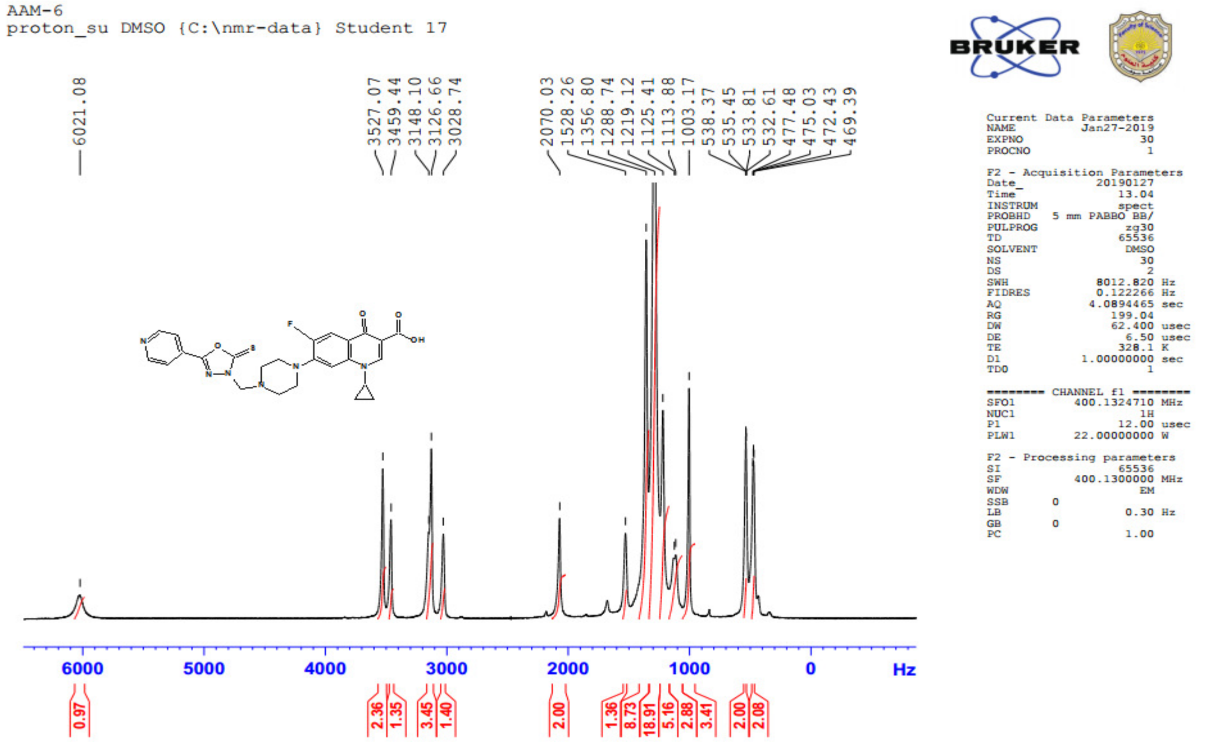

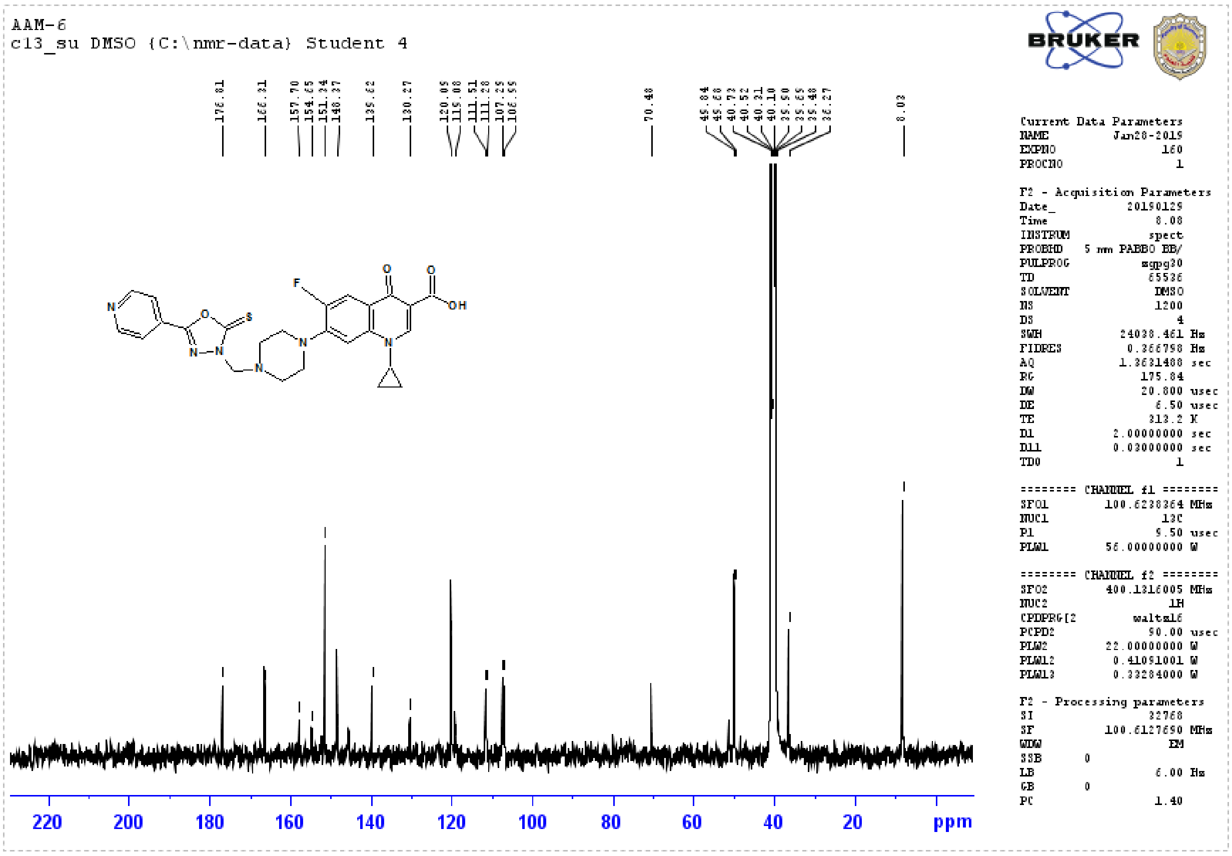

S7- ^{1}H, ^{13}C NMR and dept-135 Spectra of 7

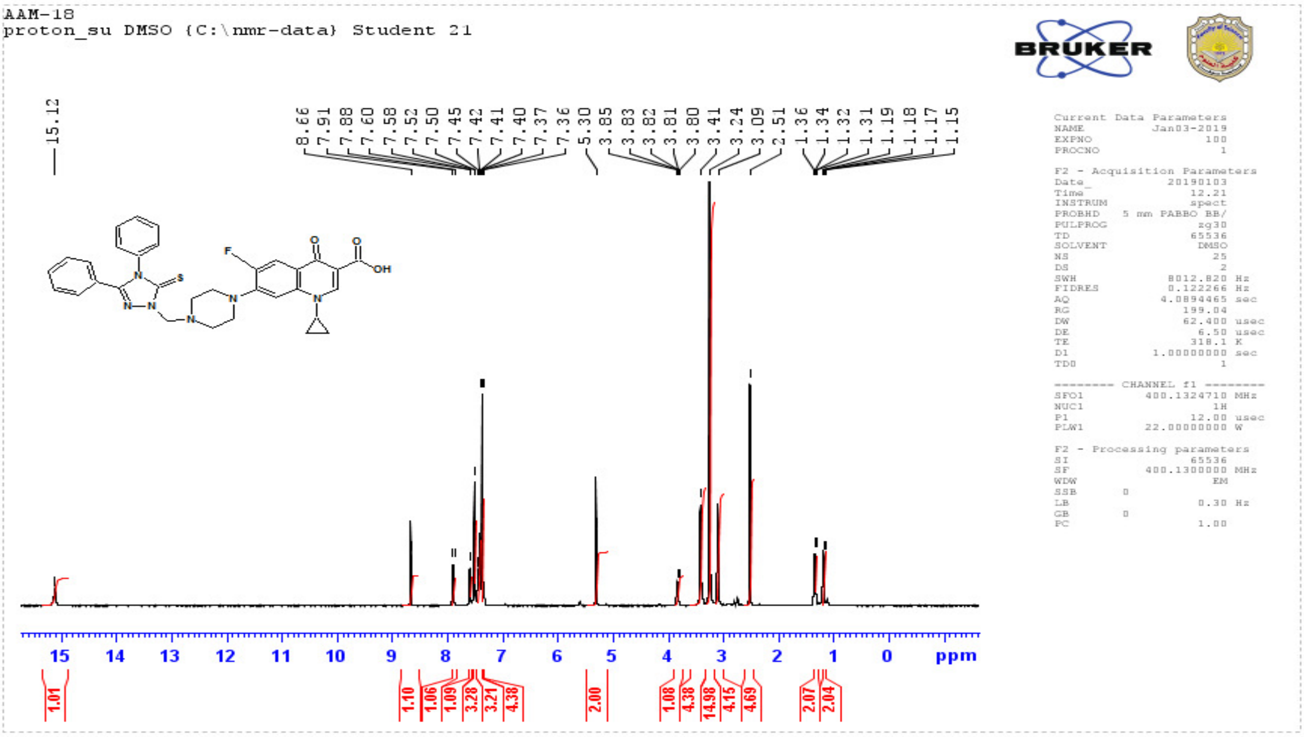

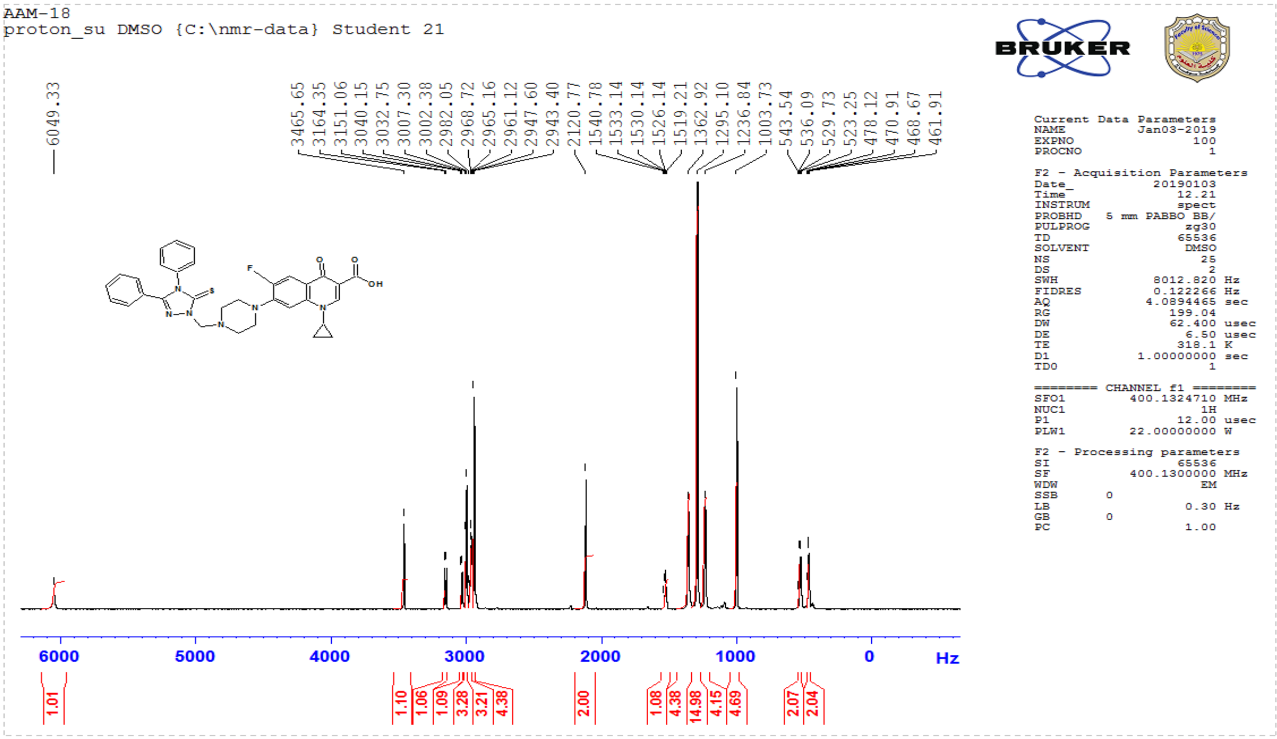
AAM-18
proton_su DMSO {C:\nmr-data} Student 21
6049.33
3465.65 3164.35 3151.06 3040.15 3032.75 3007.30 3002.38 2982.05 2968.72 2965.16 2961.12 2947.60 2943.40 2120.77 1540.78 1533.14 1530.14 1526.14 1519.21 1362.92 1295.10 1236.84 1003.73 543.54 536.09 529.73 523.25 478.12 470.91 468.67 461.91
BRUKER
Current Data Parameters
NAME Jan03-2019
EXPNO 100
PROCNO 1
F2 - Acquisition Parameters
Date_ 20190103
Time 12.21
INSTRUM spect
PROBHD 5 mm PABBO BB/
PULPROG zg30
TD 65536
SOLVENT DMSO
NS 25
DS 2
SWH 8012.820 Hz
FIDRES 0.122266 Hz
AQ 4.0894465 sec
RG 199.04
DW 62.400 usec
DE 6.50 usec
TE 318.1 K
D1 1.00000000 sec
TD0 1
======== CHANNEL f1 ========
SFO1 400.1324710 MHz
NUC1 1H
P1 12.00 usec
PLW1 22.00000000 W
F2 - Processing parameters
SI 65536
SF 400.1300000 MHz
WDW EM
SSB 0
LB 0.30 Hz
GB 0
PC 1.00
6000 5000 4000 3000 2000 1000 0 Hz
1.01 1.10 1.06 1.09 3.28 3.21 4.38 2.00 1.08 4.38 14.98 4.15 4.69 2.07 2.04

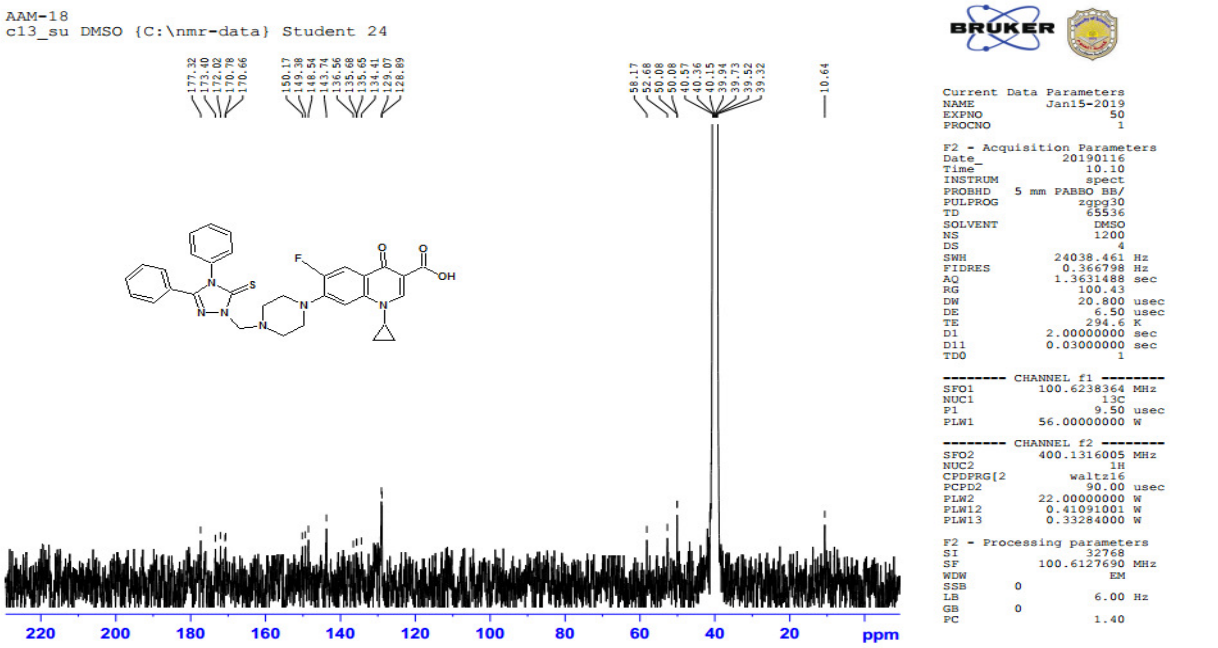
AAM-18
c13_su DMSO {C:\nmr-data} Student 24
177.32 173.40 172.02 170.78 170.66 150.17 149.38 148.54 143.74 136.56 135.88 135.65 134.41 129.07 128.89
58.17 52.68 50.08 50.08 40.57 40.36 40.15 39.94 39.73 39.52 39.32 10.64
BRUKER
Current Data Parameters
NAME Jan15-2019
EXPNO 50
PROCNO 1
F2 - Acquisition Parameters
Date_ 20190116
Time 10.10
INSTRUM spect
PROBHD 5 mm PABBO BB/
PULPROG zgpg30
TD 65536
SOLVENT DMSO
NS 1200
DS 4
SWH 24038.461 Hz
FIDRES 0.366798 Hz
AQ 1.3631488 sec
RG 100.43
DW 20.800 usec
DE 6.50 usec
TE 294.6 K
D1 2.00000000 sec
D11 0.03000000 sec
TD0 1
-------- CHANNEL f1 --------
SFO1 100.6238364 MHz
NUC1 13C
P1 9.50 usec
PLW1 56.00000000 W
-------- CHANNEL f2 --------
SFO2 400.1316005 MHz
NUC2 1H
CPDPRG[2 waltz16
PCPD2 90.00 usec
PLW2 22.00000000 W
PLW12 0.41091001 W
PLW13 0.33284000 W
F2 - Processing parameters
SI 32768
SF 100.6127690 MHz
WDW EM
SSB 0
LB 6.00 Hz
GB 0
PC 1.40
220 200 180 160 140 120 100 80 60 40 20 ppm

S8- ^{1}H, ^{13}C NMR and dept-135 Spectra of 8

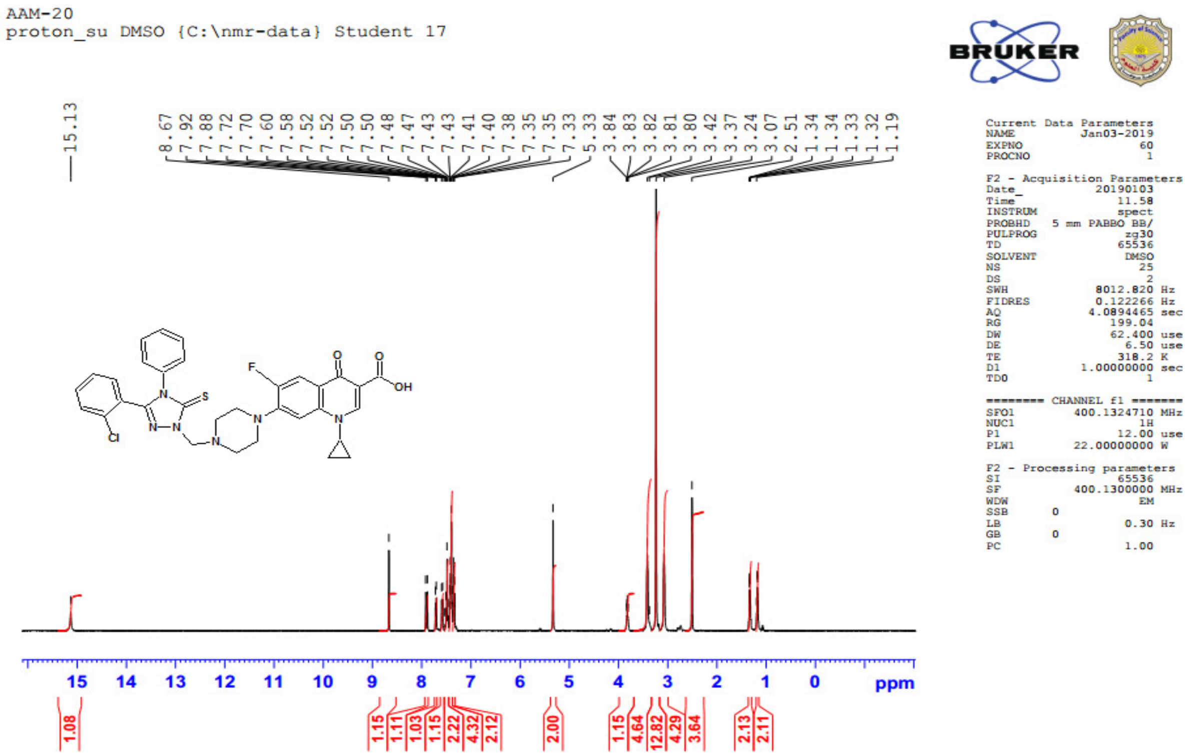

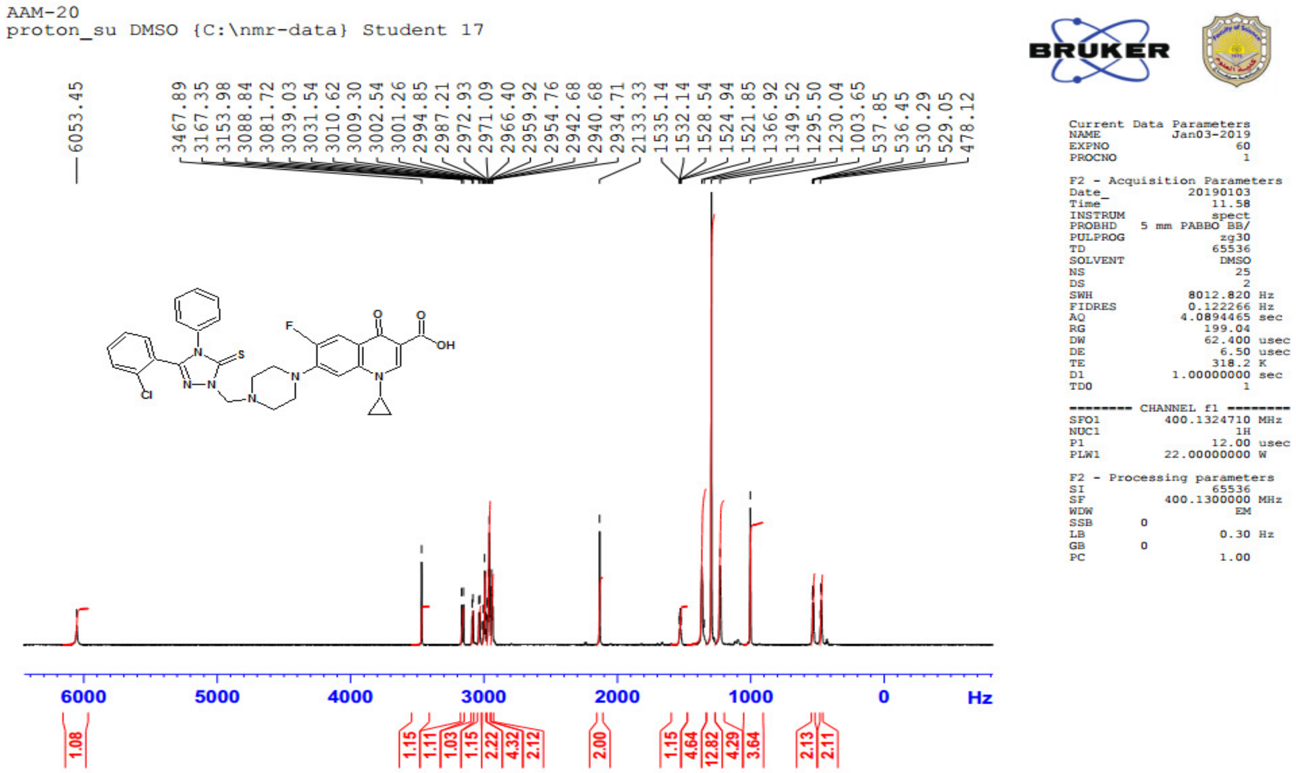

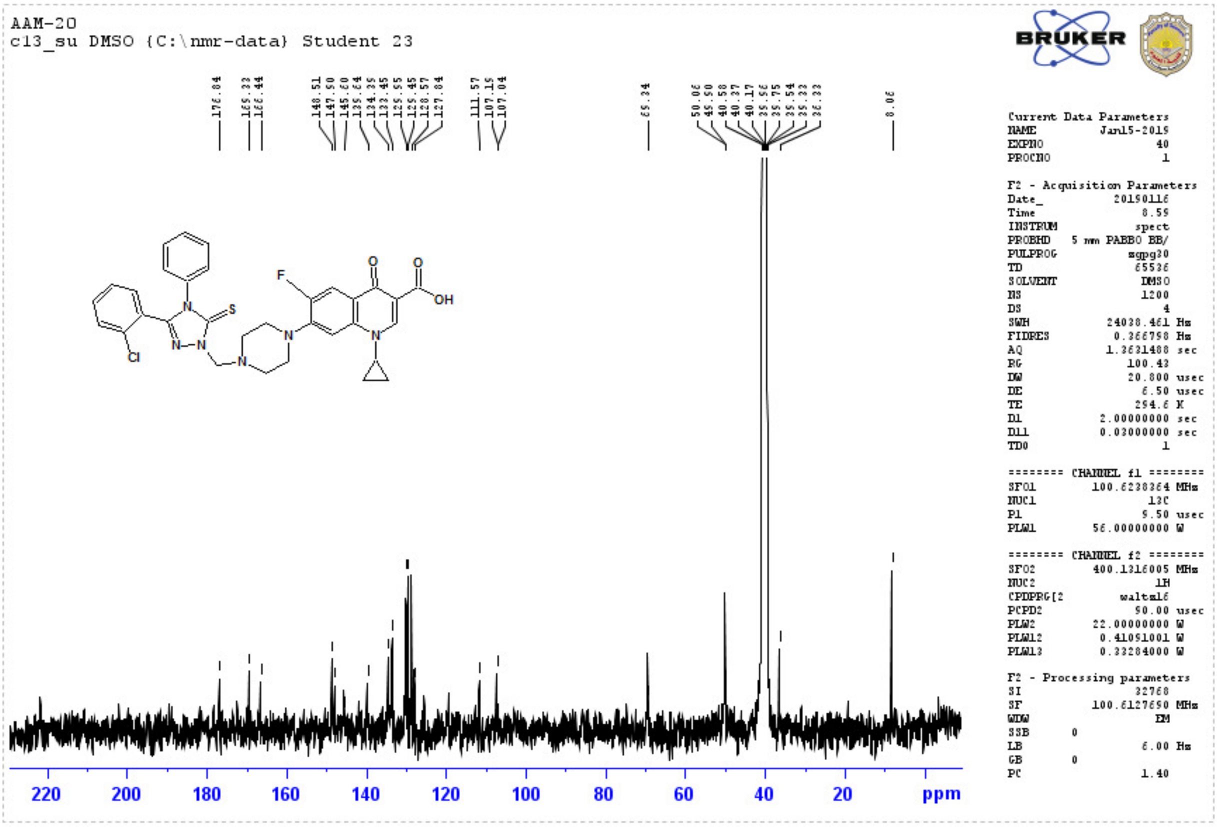

S9- ^{1}H, ^{13}C NMR and dept-135 Spectra of 9

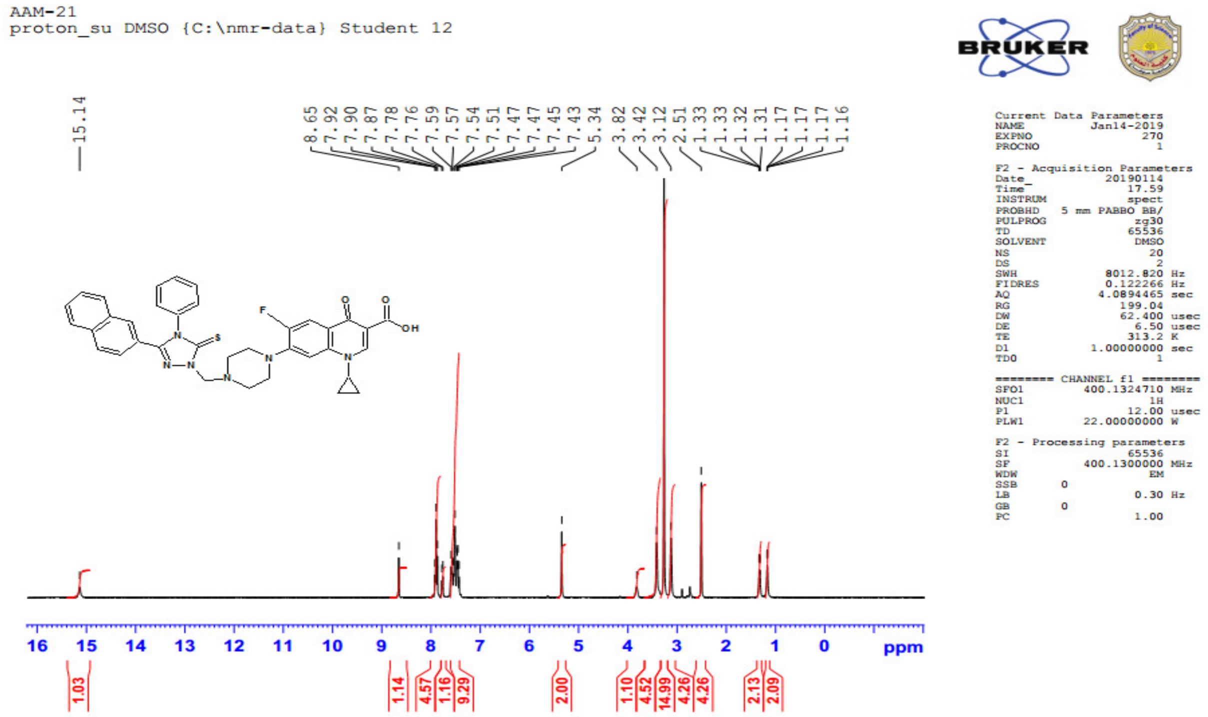

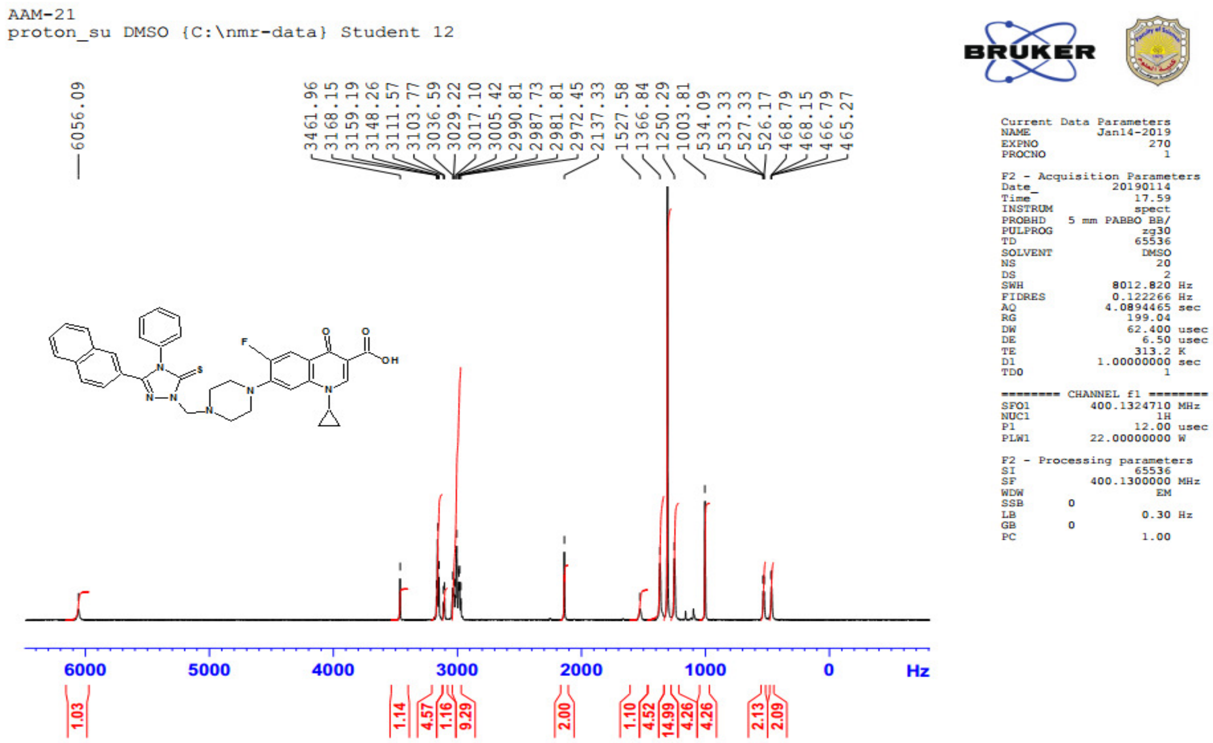
AAM-21
proton_su DMSO {C:\nmr-data} Student 12
6056.09
3461.96
3168.15
3159.19
3148.26
3111.57
3103.77
3036.59
3029.22
3017.10
3005.42
2990.81
2987.73
2981.81
2972.45
2137.33
1527.58
1366.84
1250.29
1003.81
534.09
533.33
527.33
526.17
468.79
468.15
466.79
465.27
BRUKER
Current Data Parameters
NAME Jan14-2019
EXPNO 270
PROCNO 1
F2 - Acquisition Parameters
Date_ 20190114
Time 17.59
INSTRUM spect
PROBHD 5 mm PABBO BB/
PULPROG zg30
TD 65536
SOLVENT DMSO
NS 20
DS 2
SWH 8012.820 Hz
FIDRES 0.122266 Hz
AQ 4.0894465 sec
RG 199.04
DW 62.400 usec
DE 6.50 usec
TE 313.2 K
D1 1.00000000 sec
TD0 1
======== CHANNEL f1 ========
SFO1 400.1324710 MHz
NUC1 1H
P1 12.00 usec
PLW1 22.00000000 W
F2 - Processing parameters
SI 65536
SF 400.1300000 MHz
WDW EM
SSB 0
LB 0.30 Hz
GB 0
PC 1.00
6000
5000
4000
3000
2000
1000
0
Hz
1.03
1.14
4.57
1.16
9.29
2.00
1.10
4.52
14.99
4.26
4.26
2.13
2.09

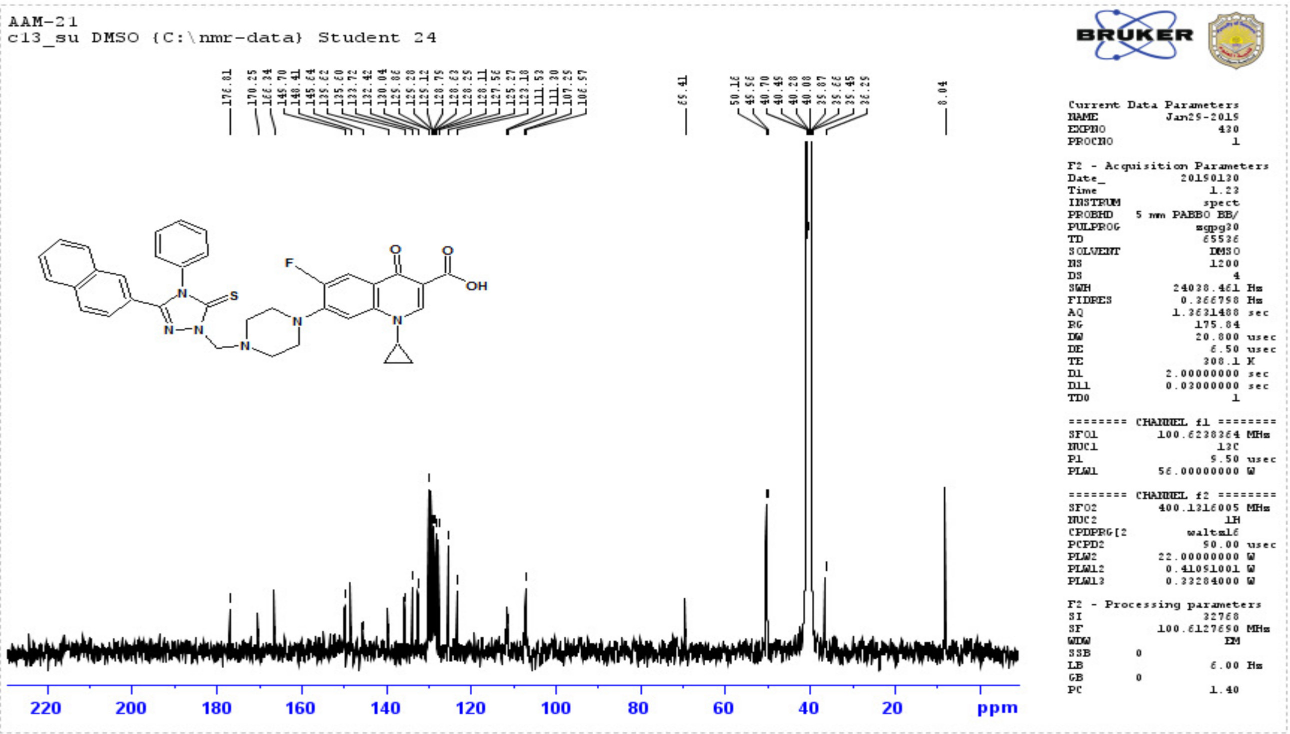
AAM-21
c13_su DMSO {C:\nmr-data} Student 24
BRUKER
Current Data Parameters
NAME Jan29-2019
EXPNO 430
PROCNO 1
F2 - Acquisition Parameters
INSTRUM spect
PROBHD 5 mm PABBO BB/
SOLVENT DMSO
NS 1200
DS 4
======== CHANNEL f1 ========
NUC1 13C
======== CHANNEL f2 ========
NUC2 1H
F2 - Processing parameters
SI 32768
WDW EM
LB 6.00 Hz
PC 1.40
220
200
180
160
140
120
100
80
60
40
20
ppm

S10- ^{1}H, ^{13}C NMR and dept-135 Spectra of 10

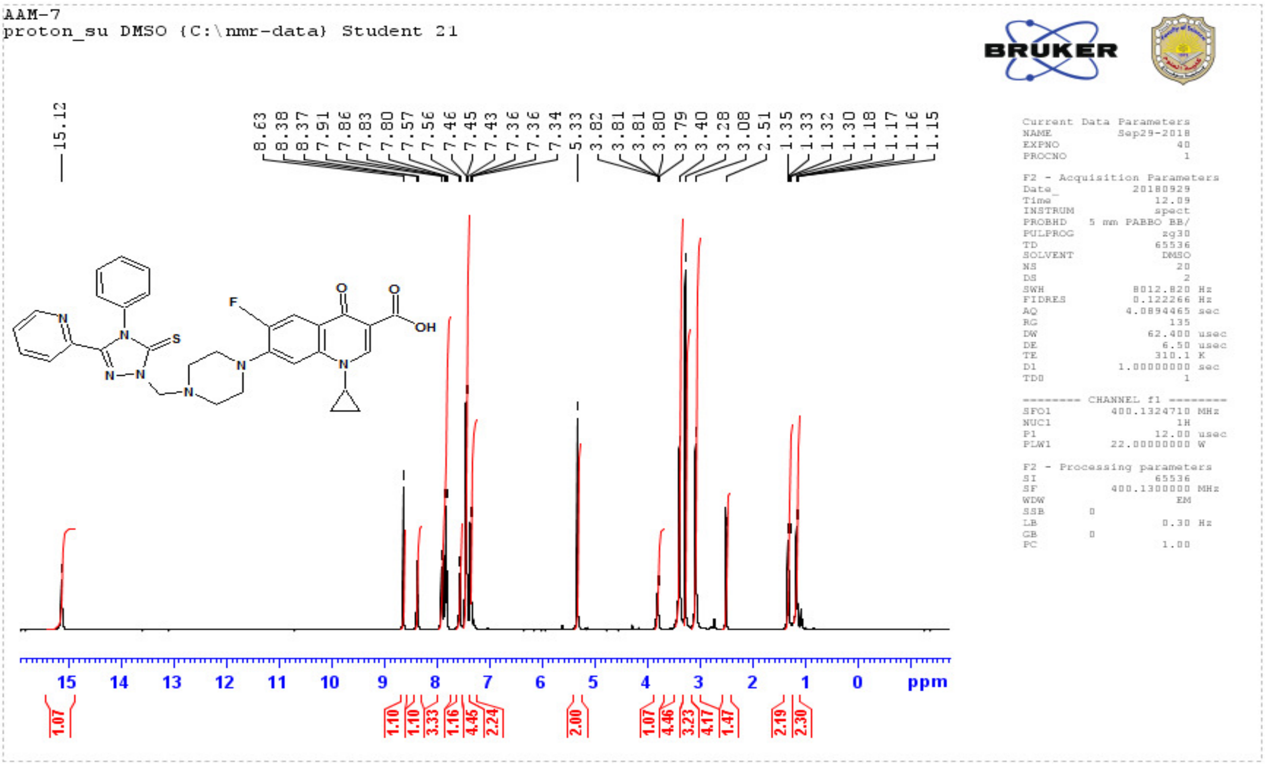

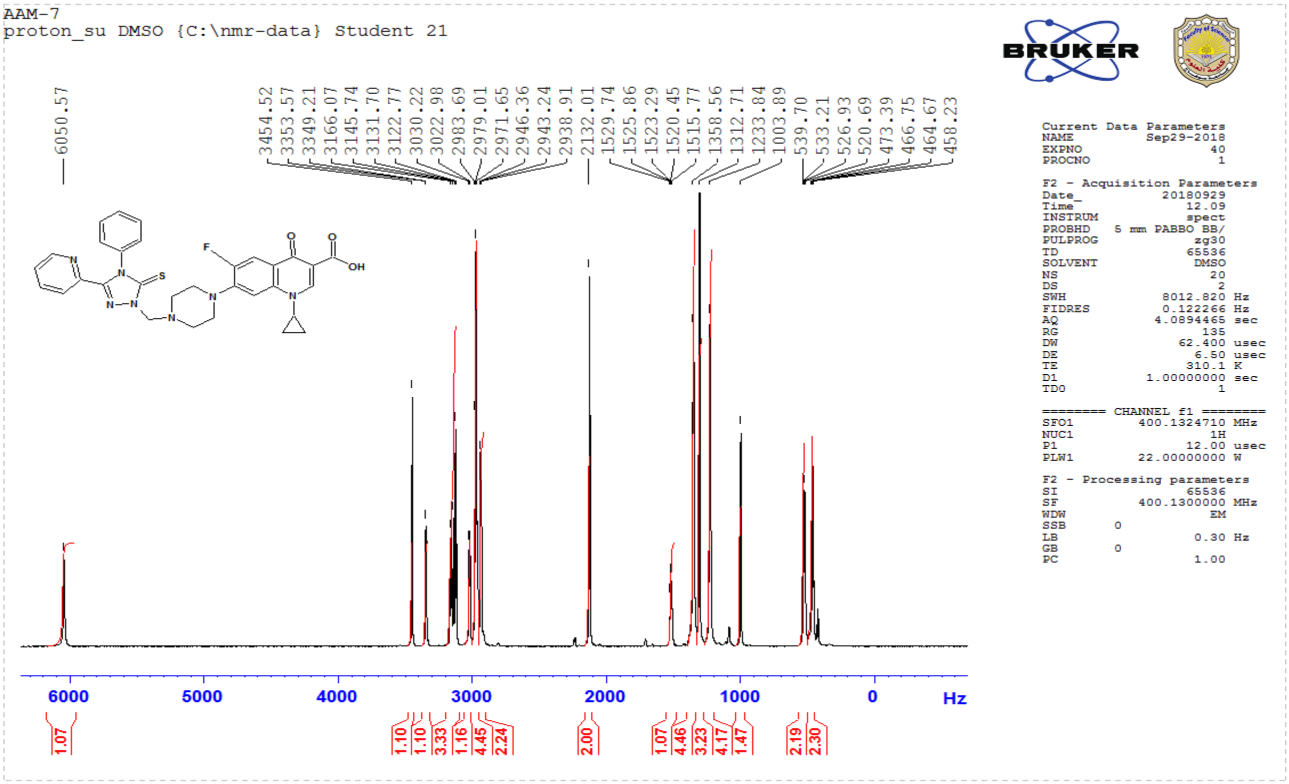

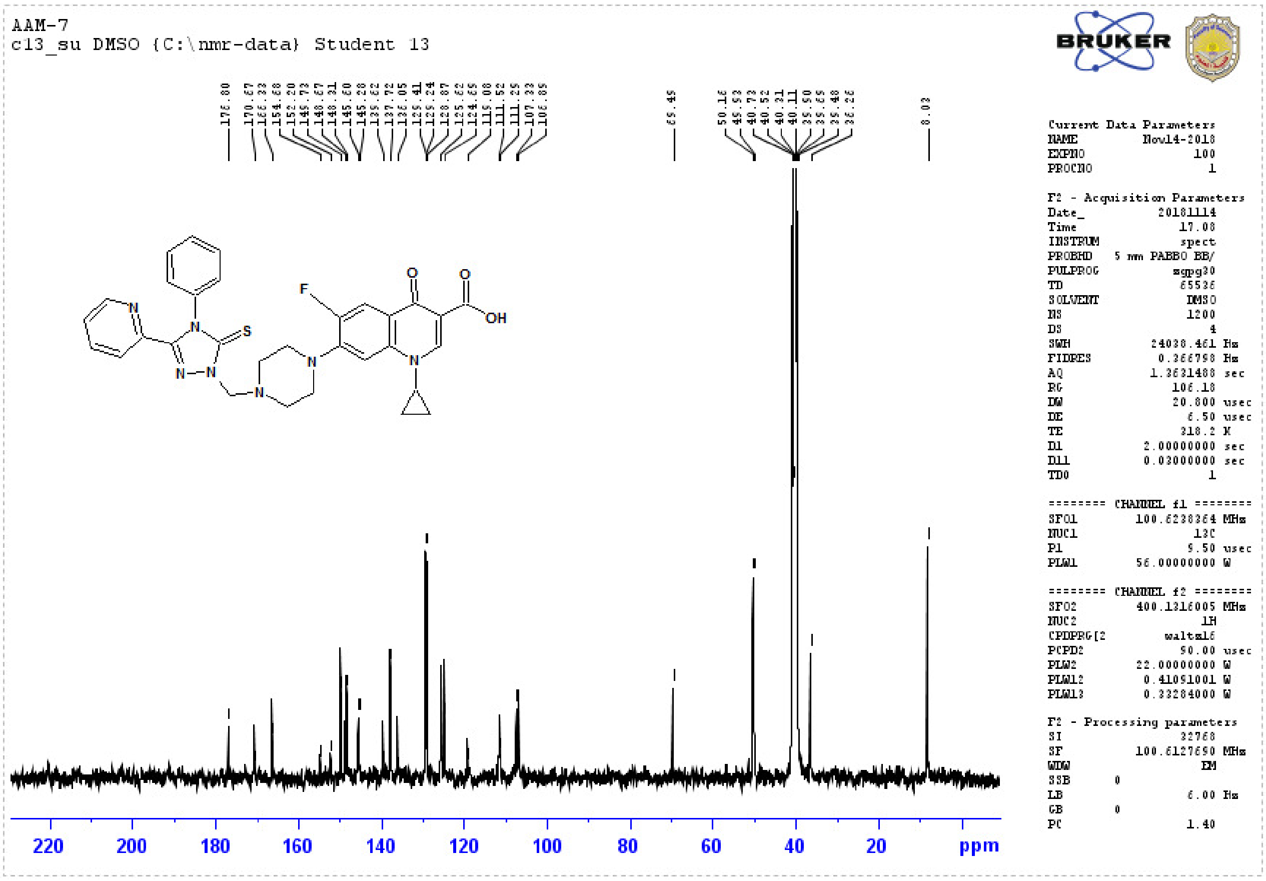

S11-[1]**H , **[13]**C NMR and dept-135 Spectra of 11**

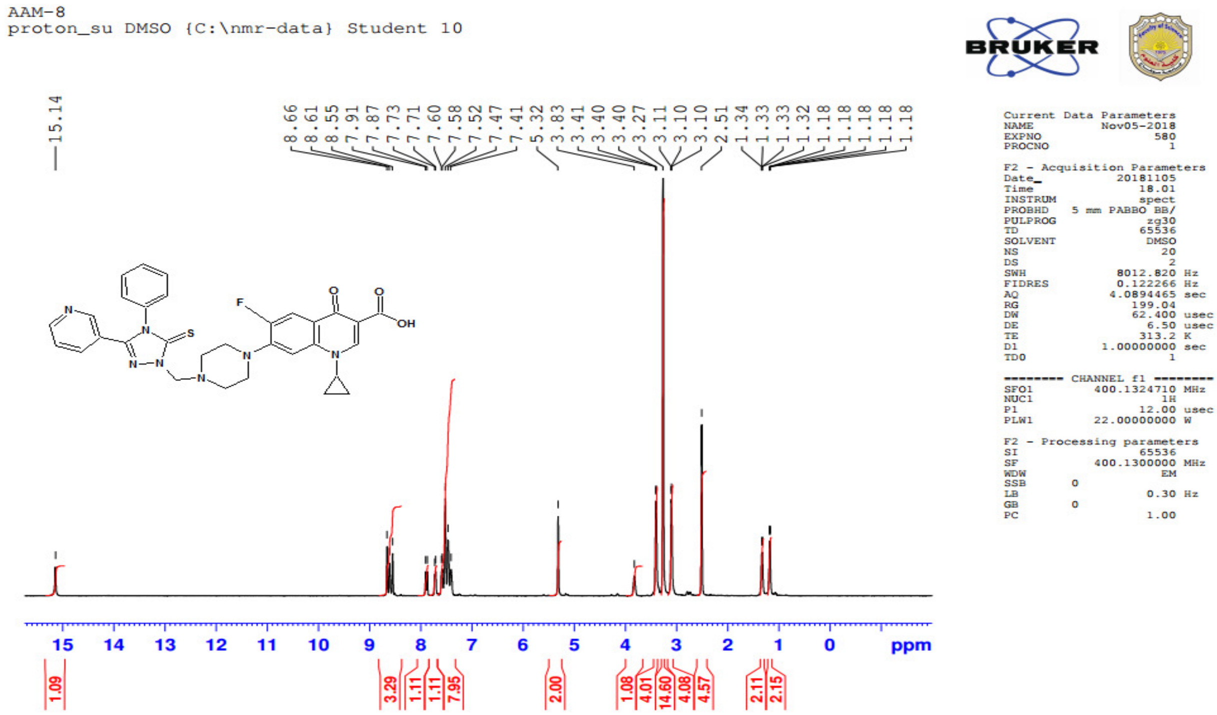

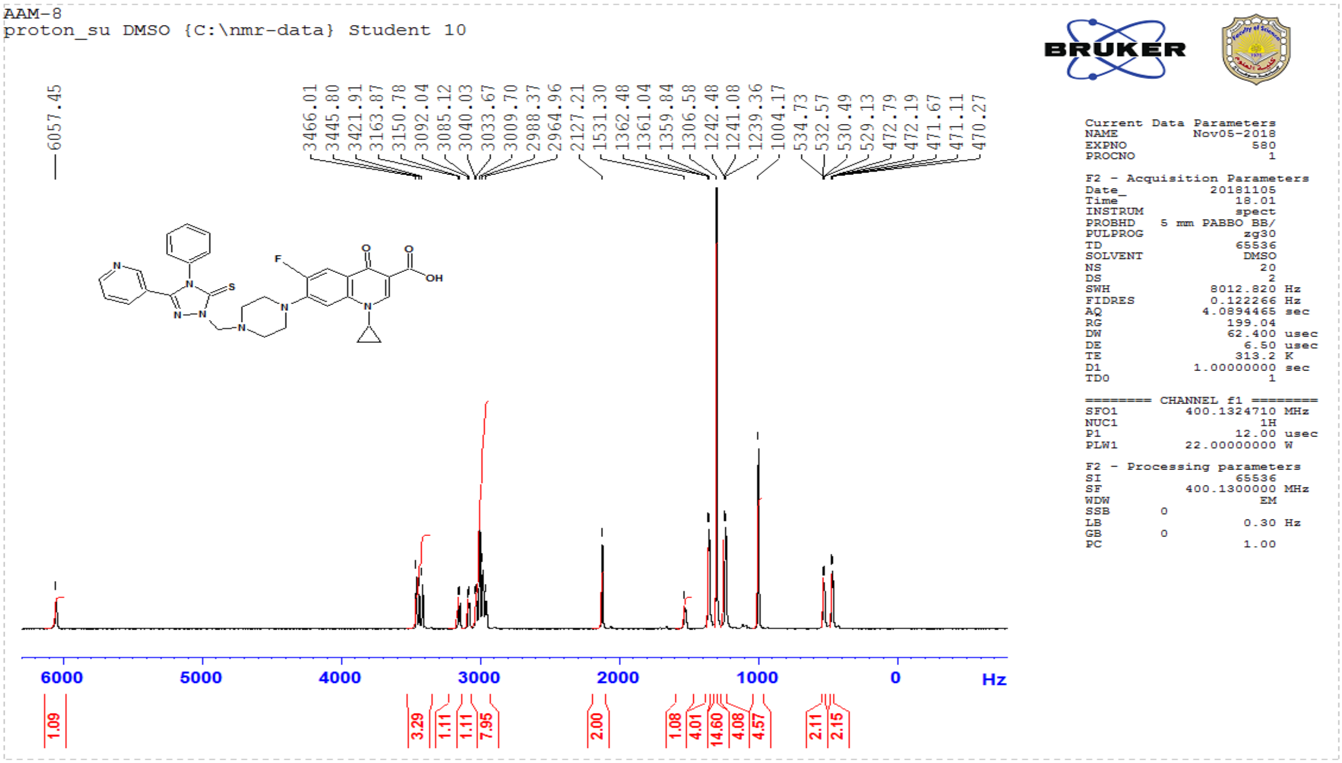
AAM-8
proton_su DMSO {C:\nmr-data} Student 10
Current Data Parameters
NAME Nov05-2018
EXPNO 580
PROCNO 1
F2 - Acquisition Parameters
Date_ 20181105
Time 18.01
INSTRUM spect
PROBHD 5 mm PABBO BB/
PULPROG zg30
TD 65536
SOLVENT DMSO
NS 20
DS 2
SWH 8012.820 Hz
FIDRES 0.122266 Hz
AQ 4.0894465 sec
RG 199.04
DW 62.400 usec
DE 6.50 usec
TE 313.2 K
D1 1.00000000 sec
TD0 1
======== CHANNEL f1 ========
SFO1 400.1324710 MHz
NUC1 1H
P1 12.00 usec
PLW1 22.00000000 W
F2 - Processing parameters
SI 65536
SF 400.1300000 MHz
WDW EM
SSB 0
LB 0.30 Hz
GB 0
PC 1.00
Hz

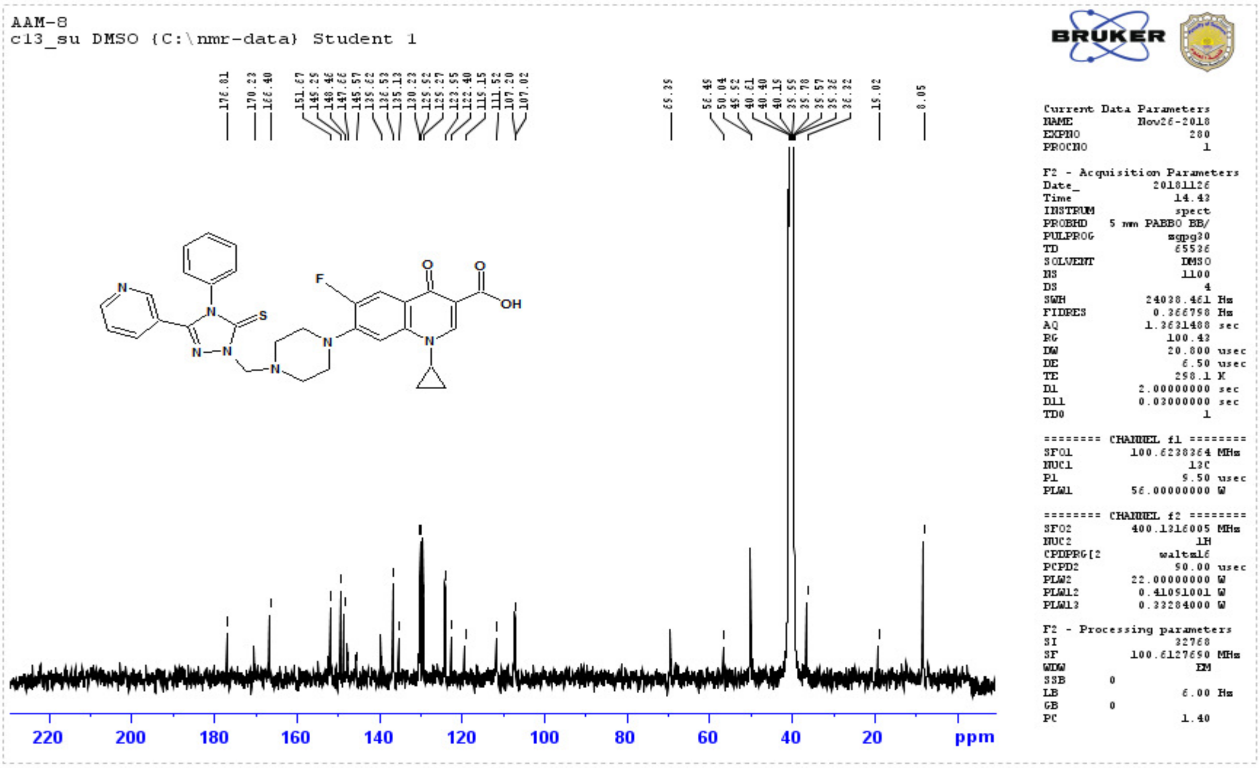
AAM-8
c13_su DMSO {C:\nmr-data} Student 1
Current Data Parameters
NAME Nov26-2018
EXPNO 280
PROCNO 1
F2 - Acquisition Parameters
ppm

S12- ^{1}H, ^{13}C NMR and dept-135 Spectra of 12

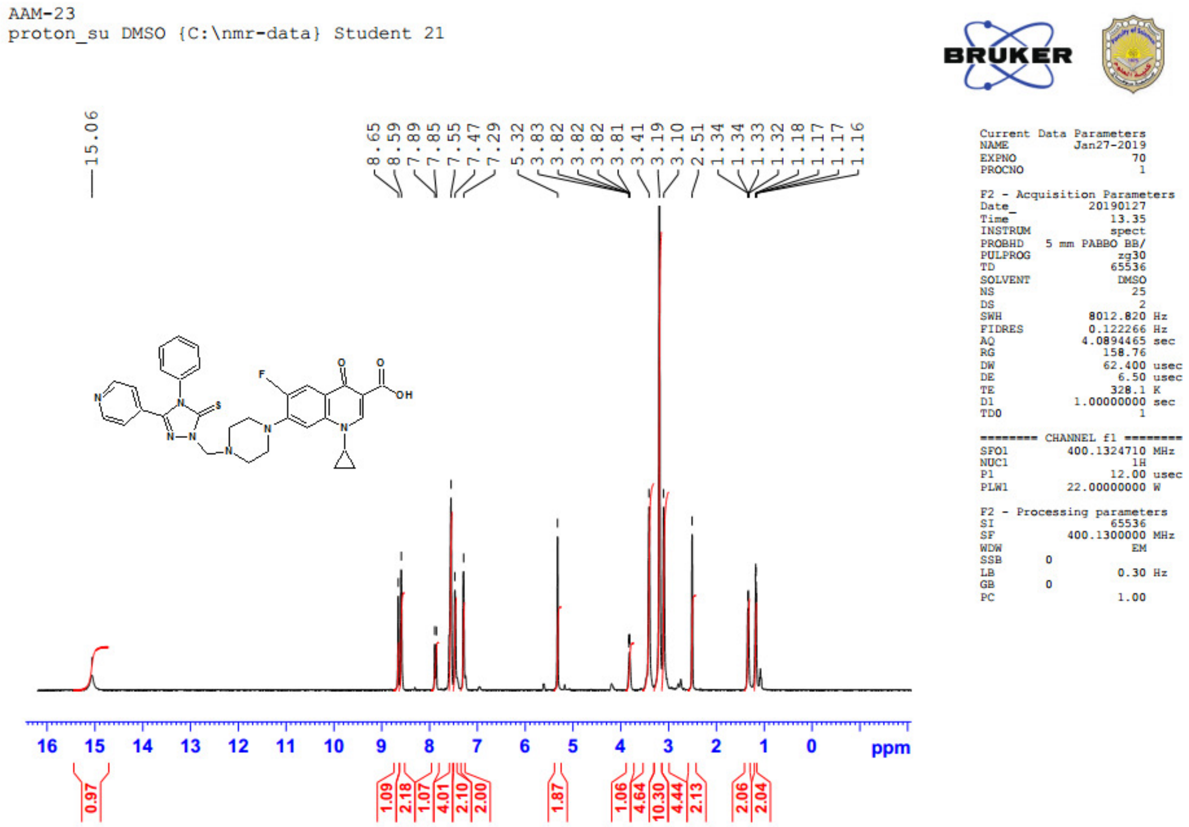

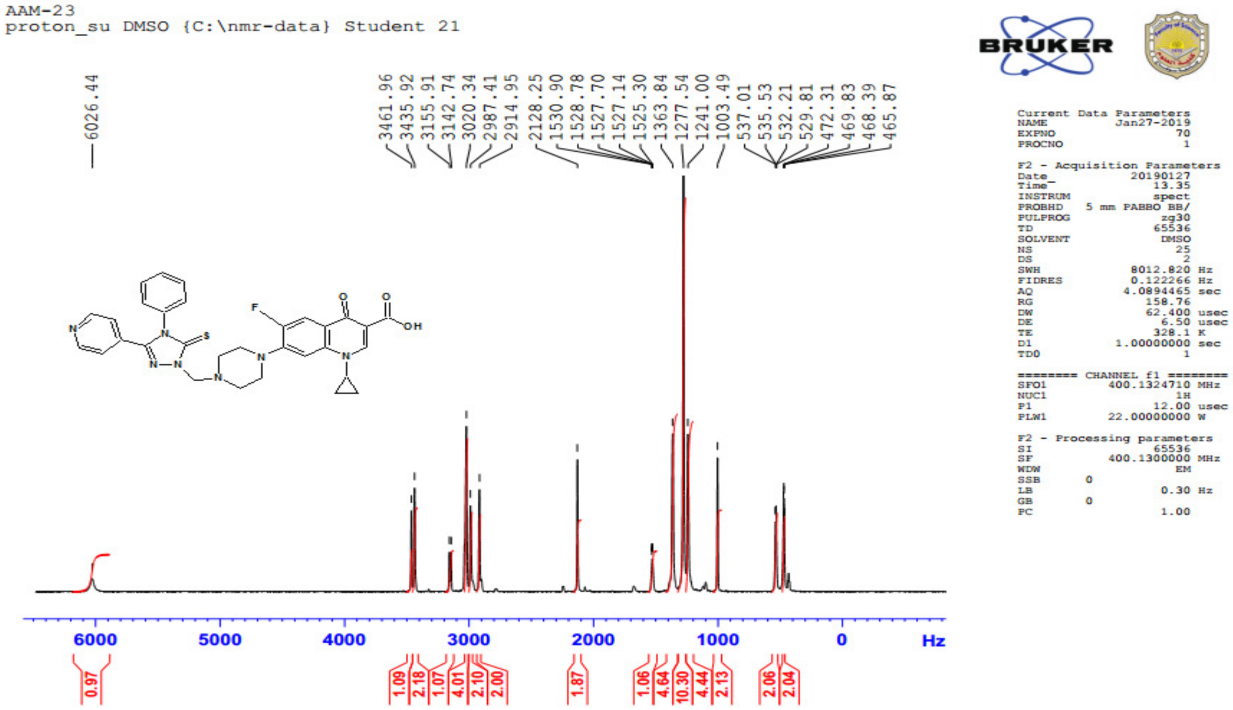

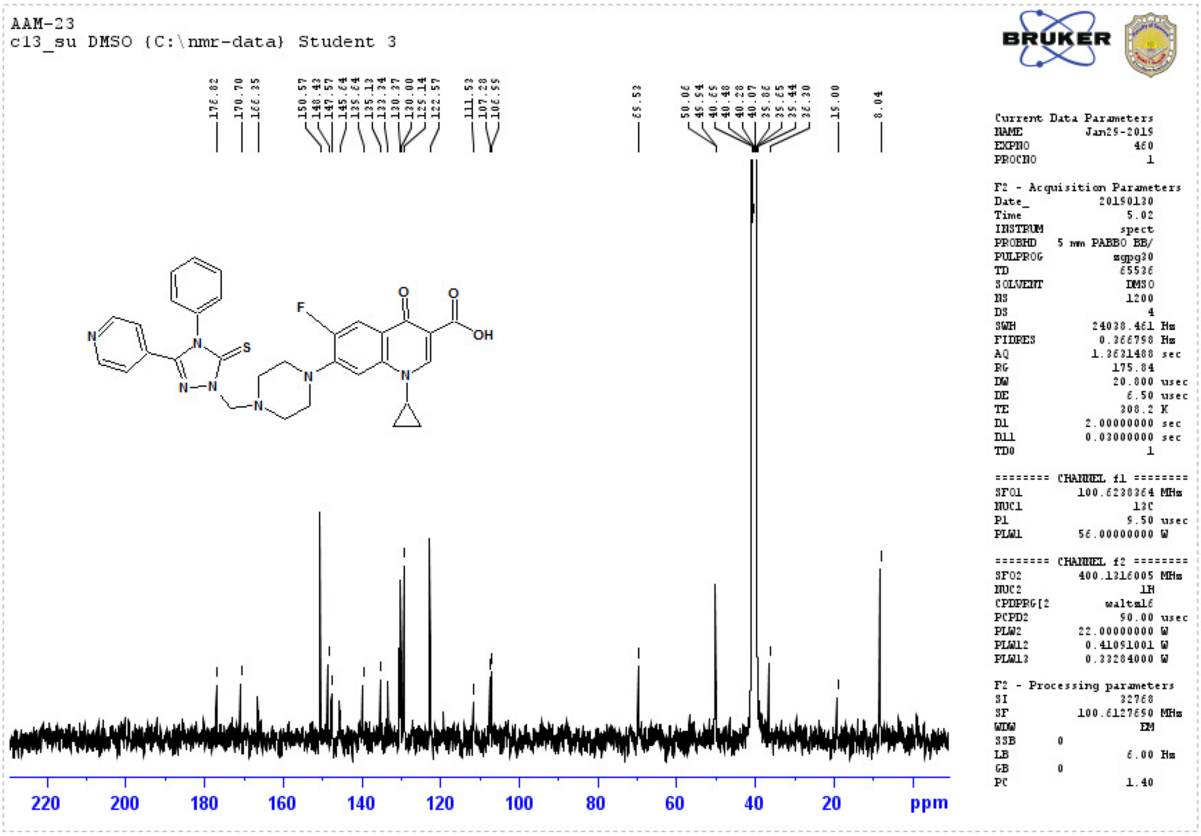

S13- ^{1}H, ^{13}C NMR and dept-135 Spectra of 13

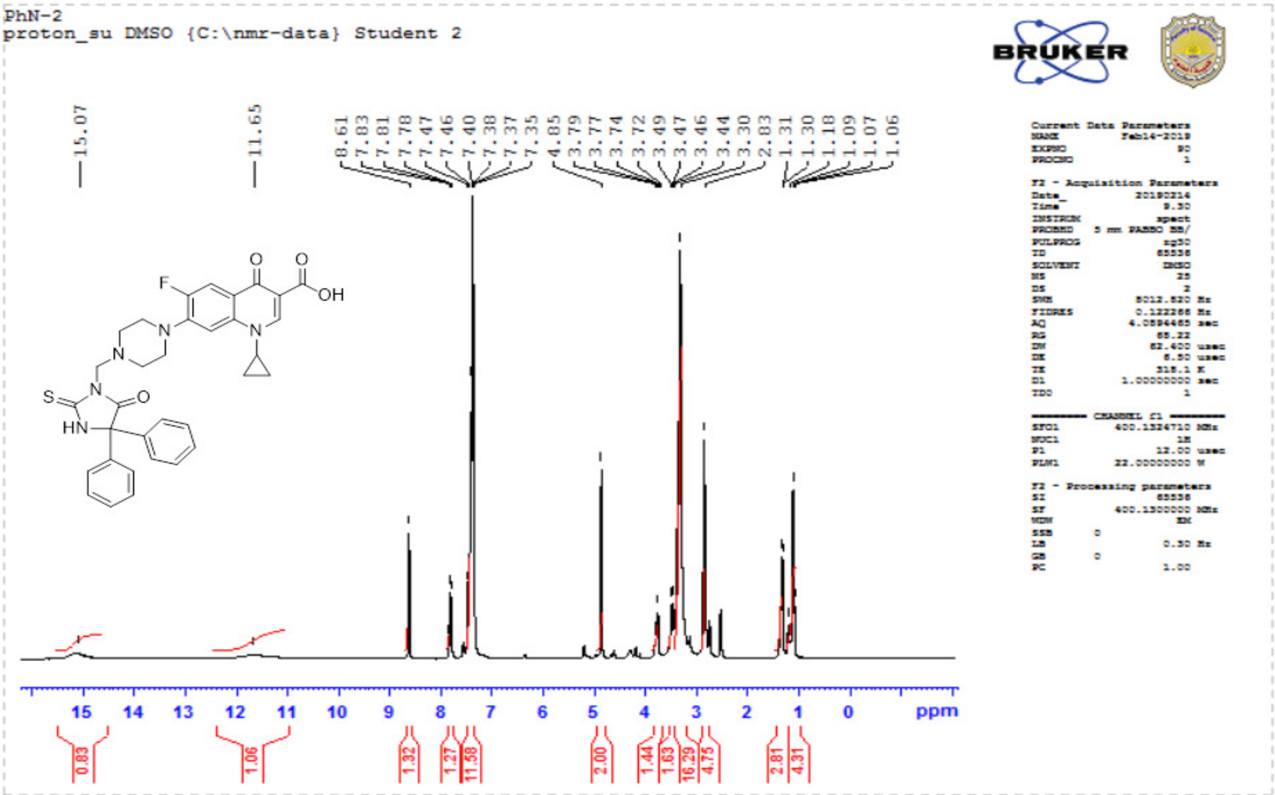

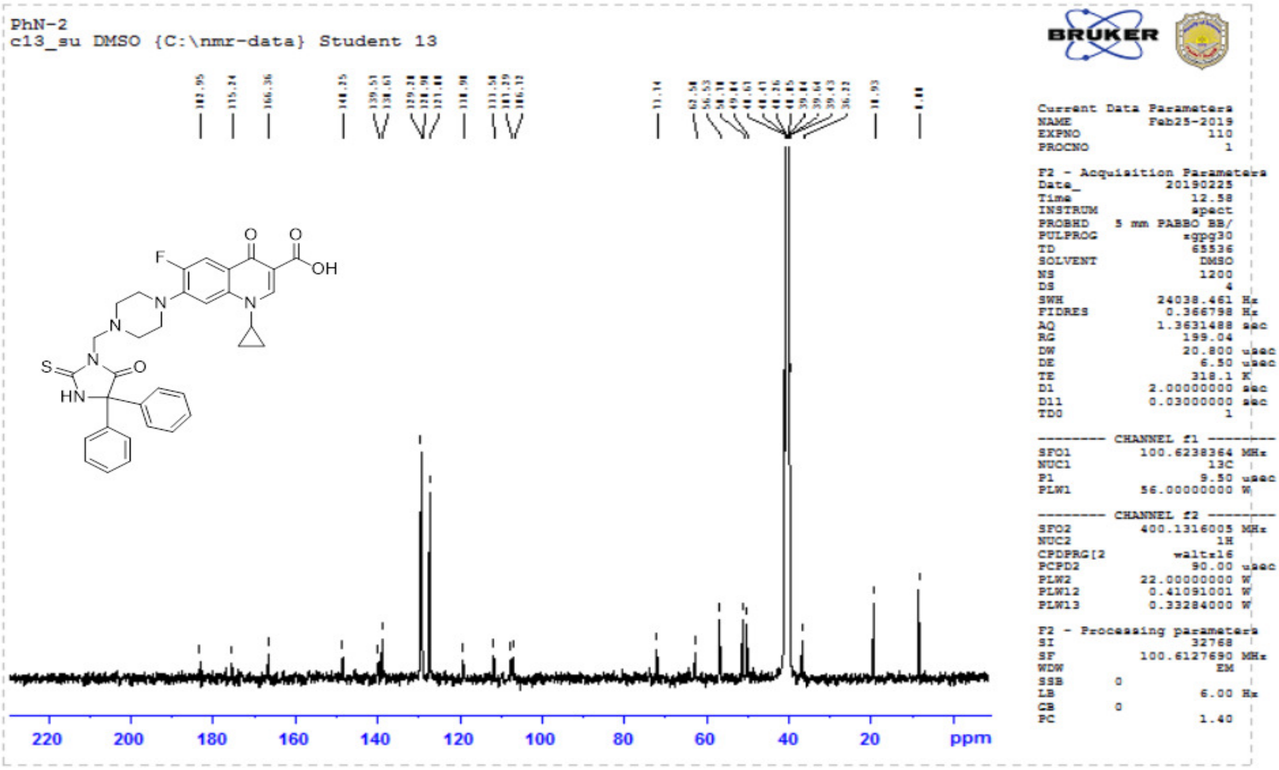

S14- ^{1}H, ^{13}C NMR and dept-135 Spectra of 14

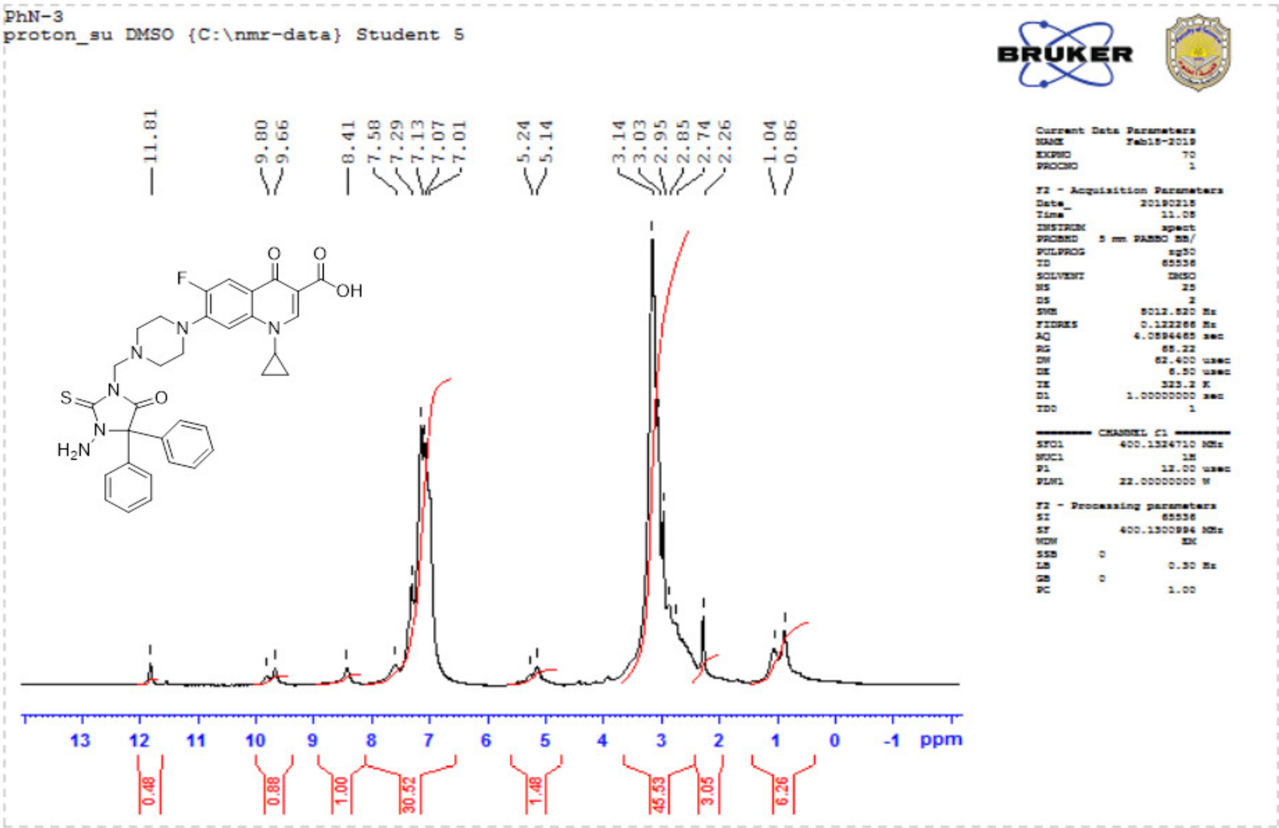

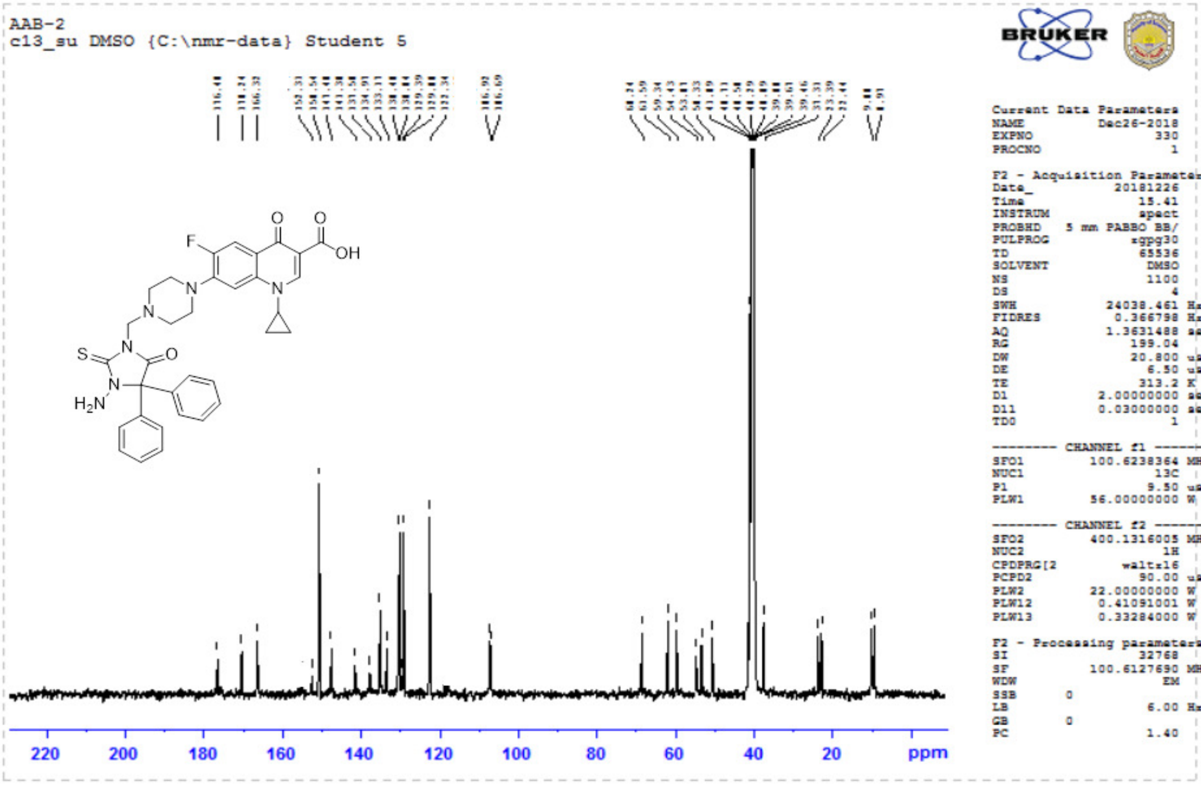

S15- ^{1}H, ^{13}C NMR and dept-135 Spectra of 15

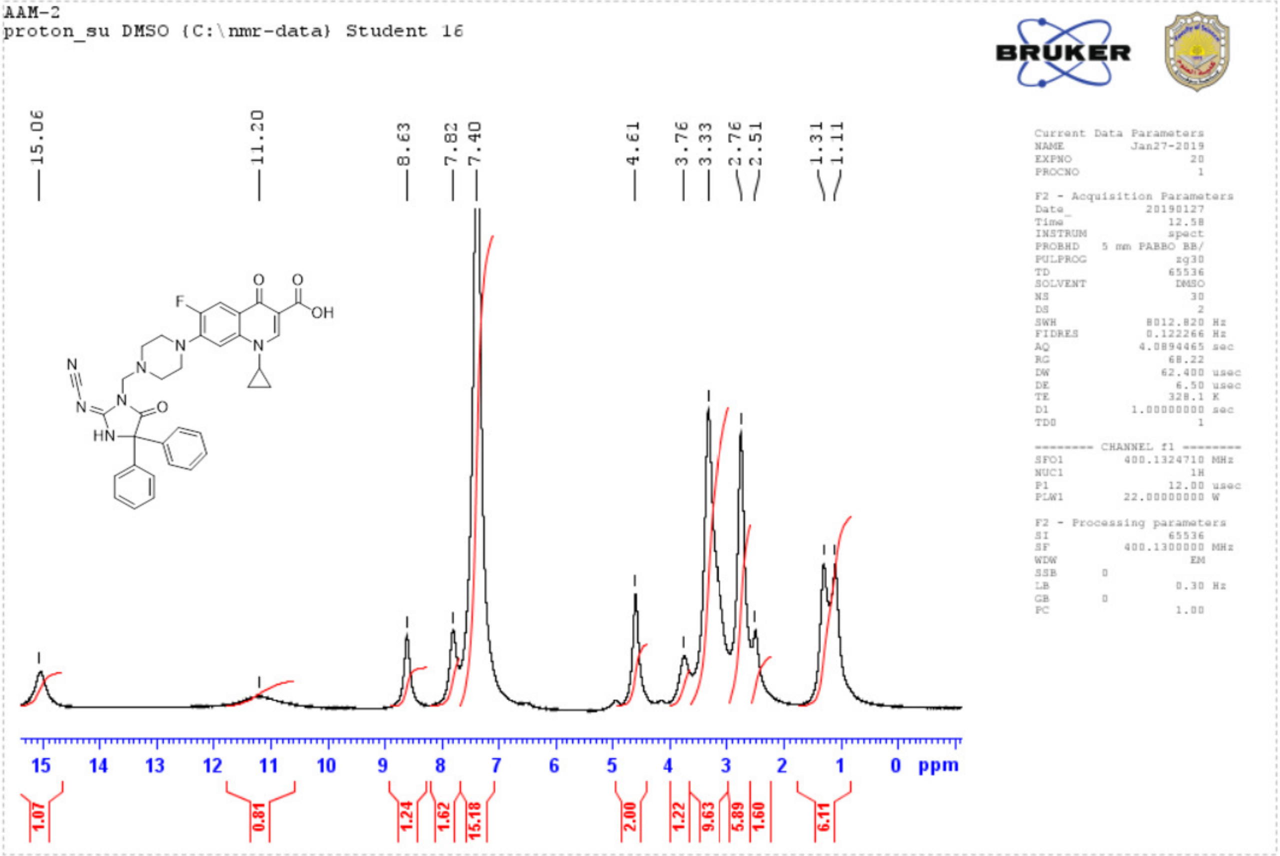

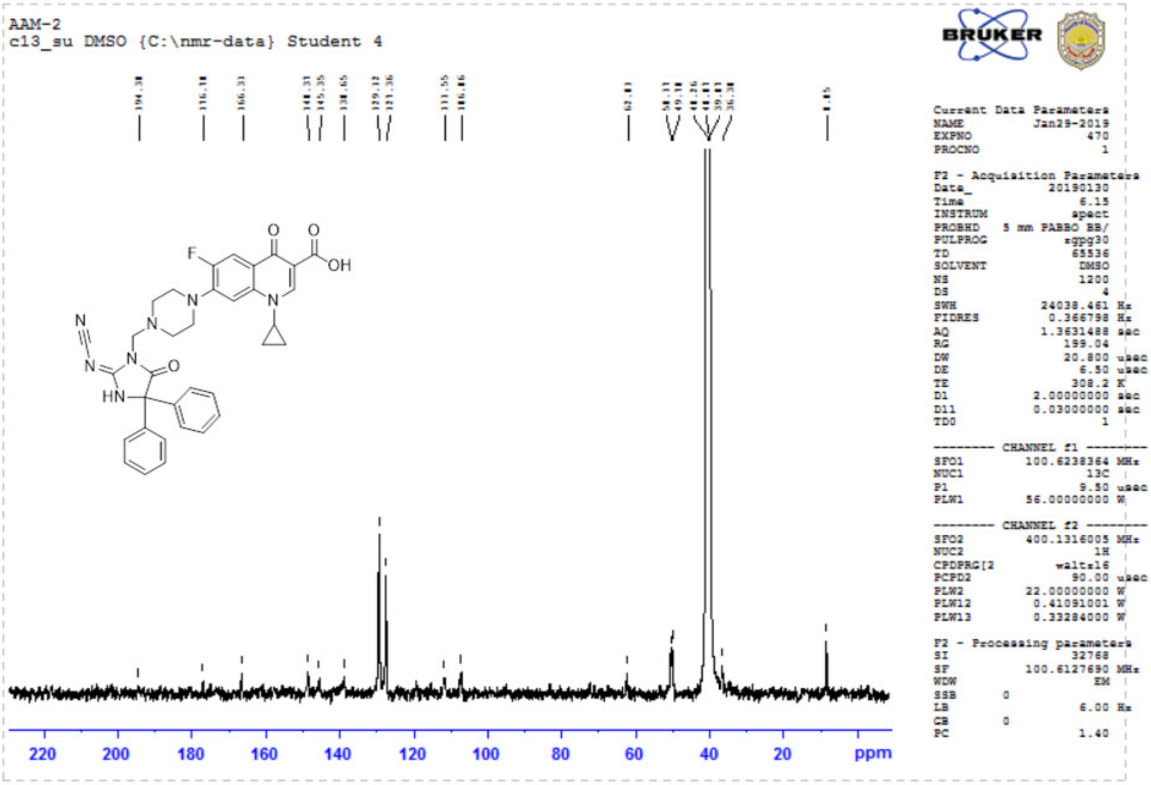

S16- ^{1}H, ^{13}C NMR and dept-135 Spectra of 16

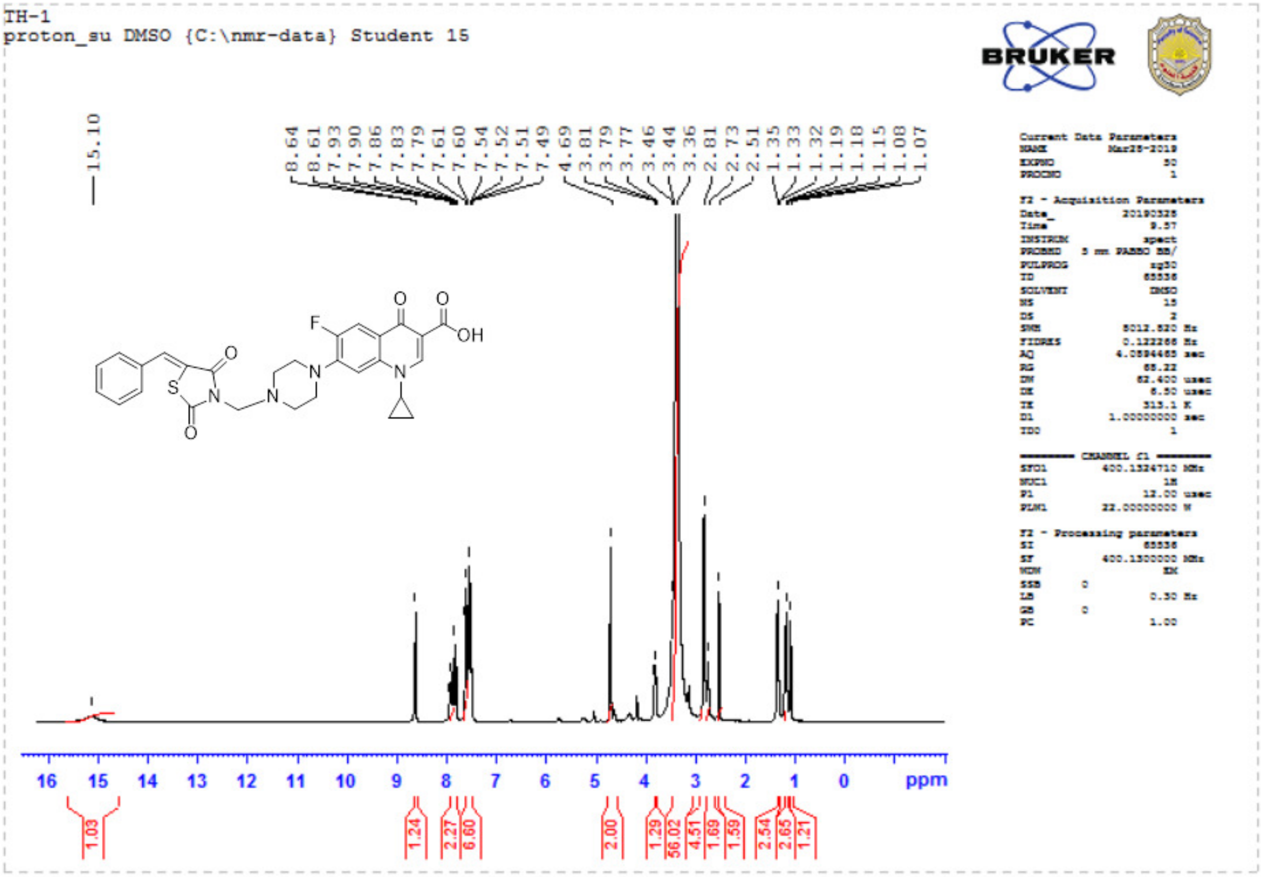

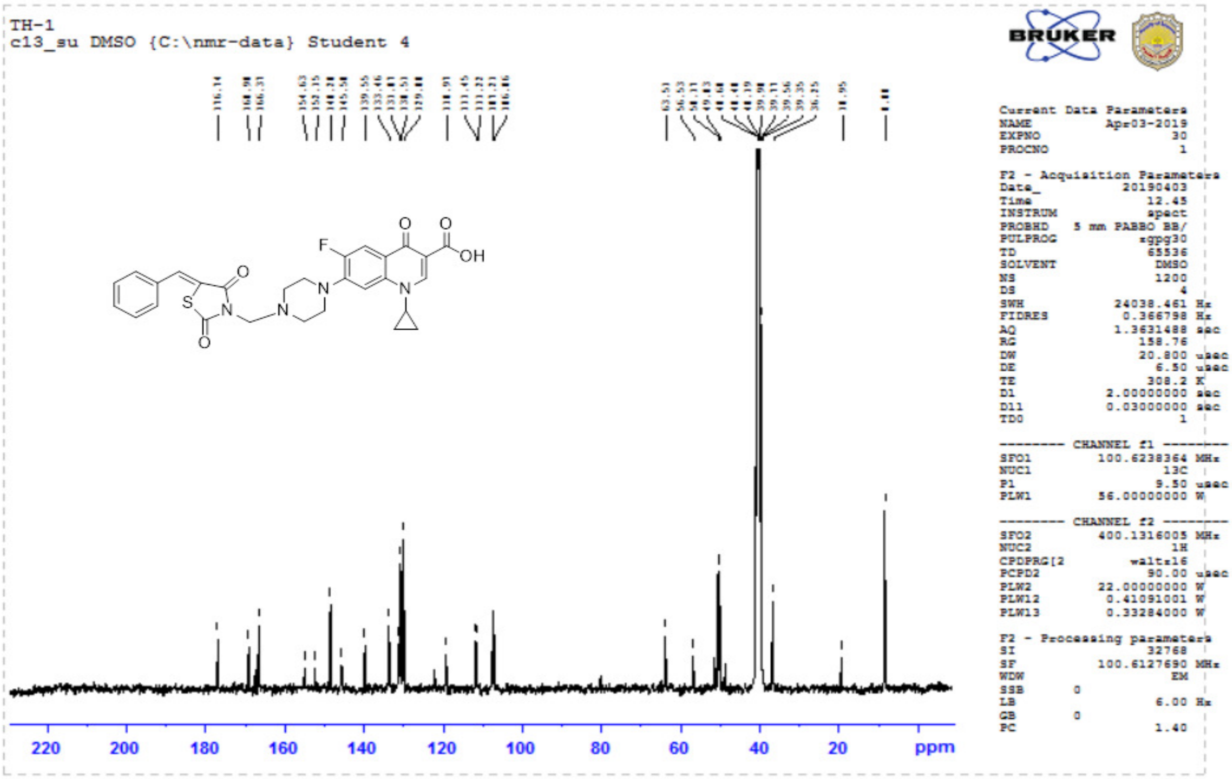

S17- ^{1}H, ^{13}C NMR and dept-135 Spectra of 17

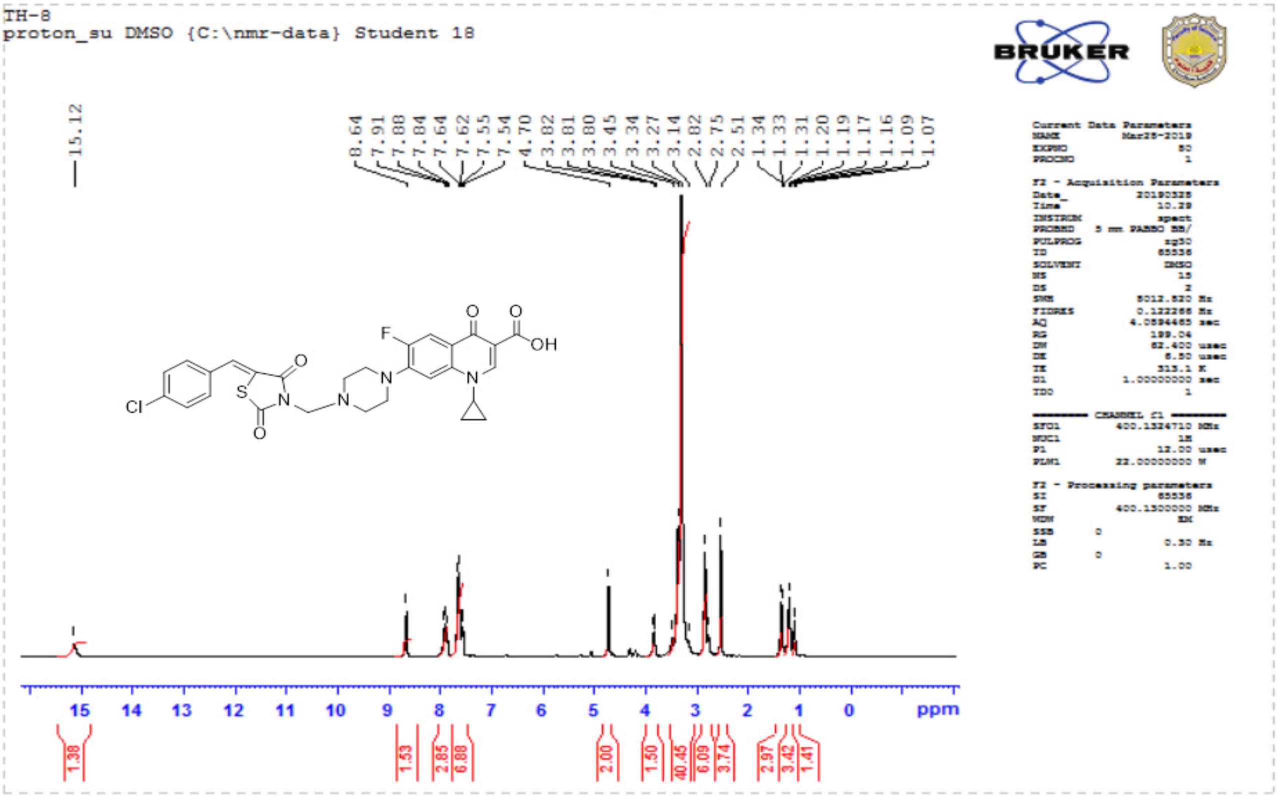

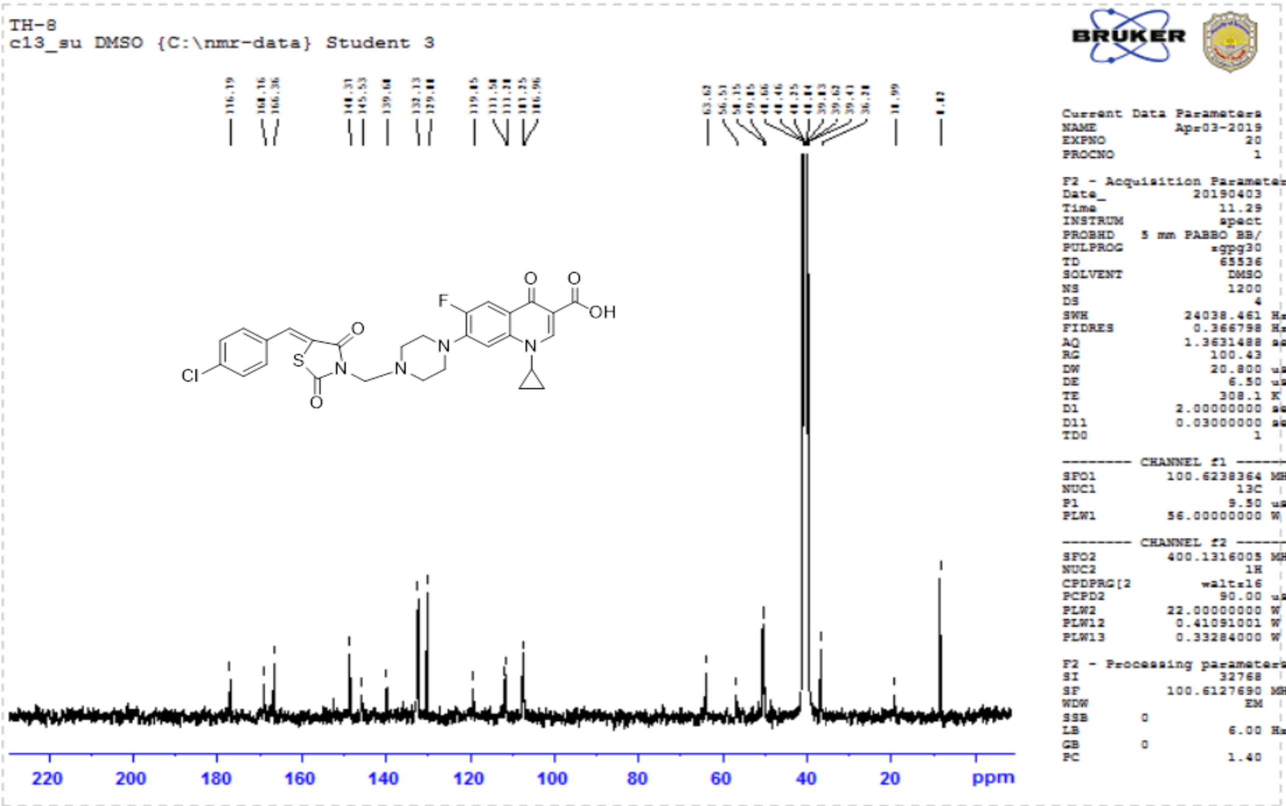

S18- ^{1}H, ^{13}C NMR and dept-135 Spectra of

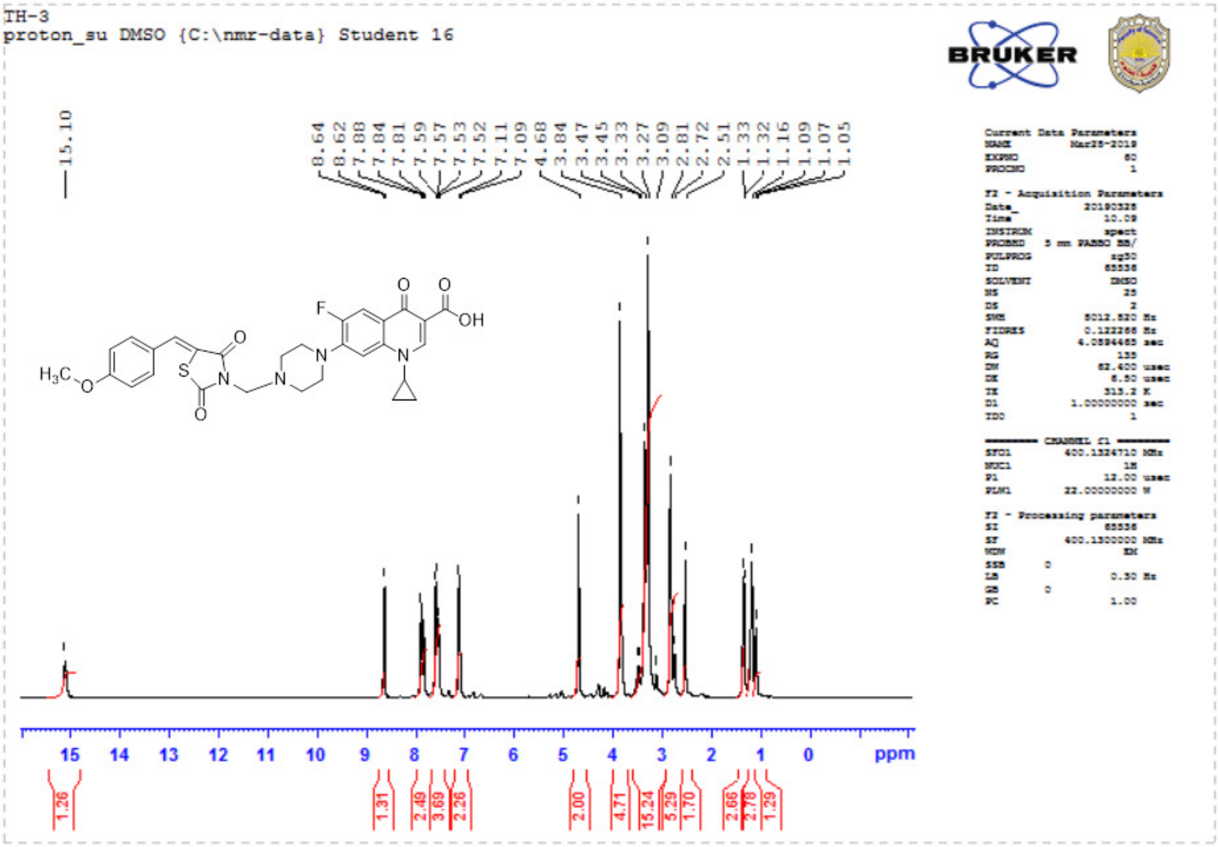

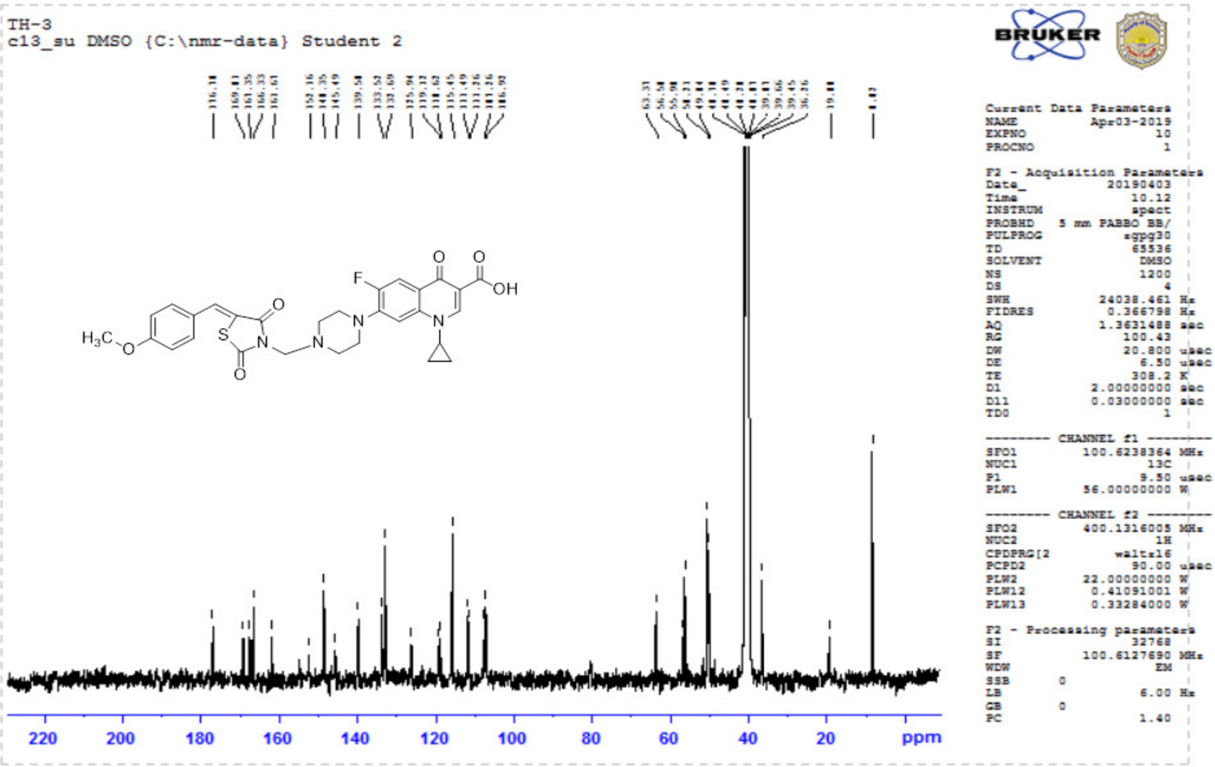

S19- ^{1}H, ^{13}C NMR and dept-135 Spectra of 19

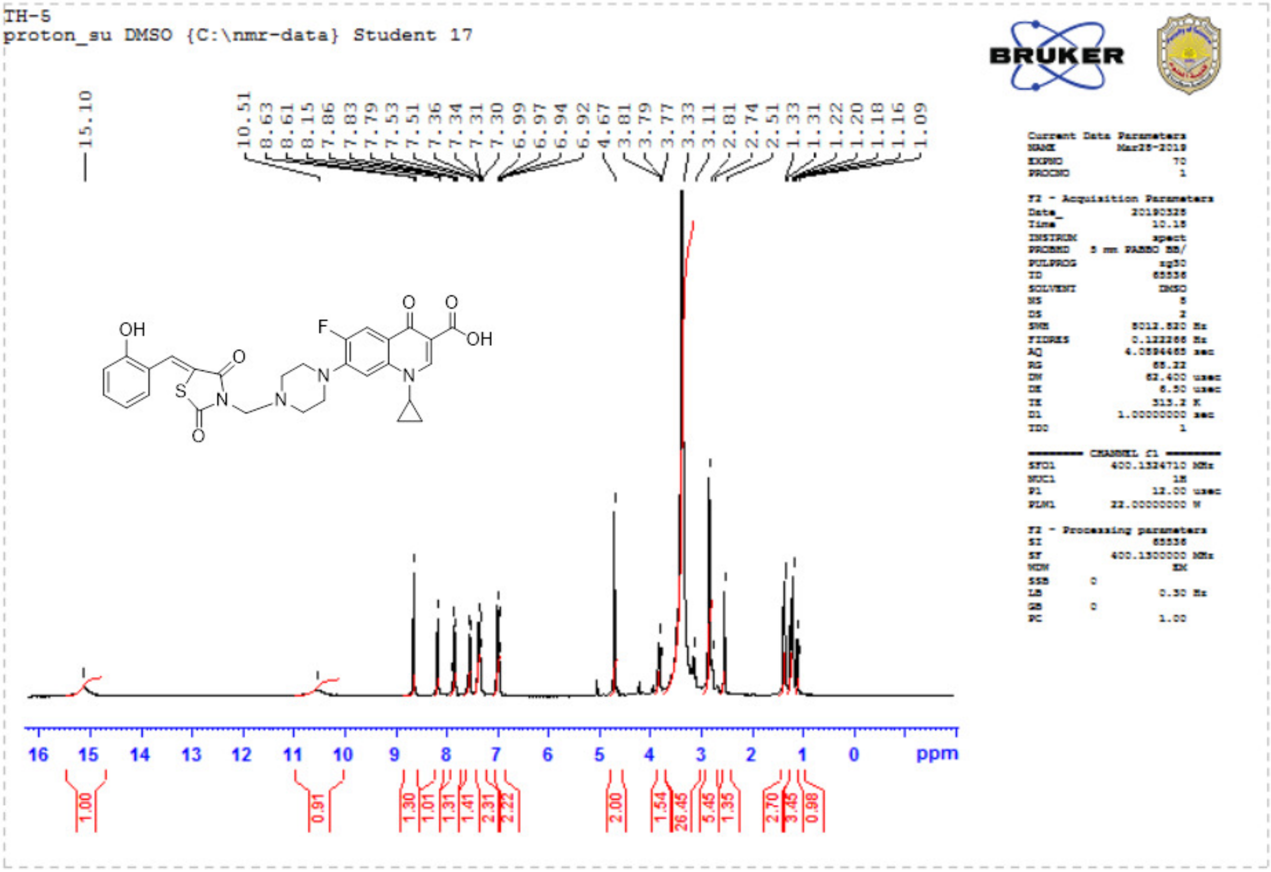

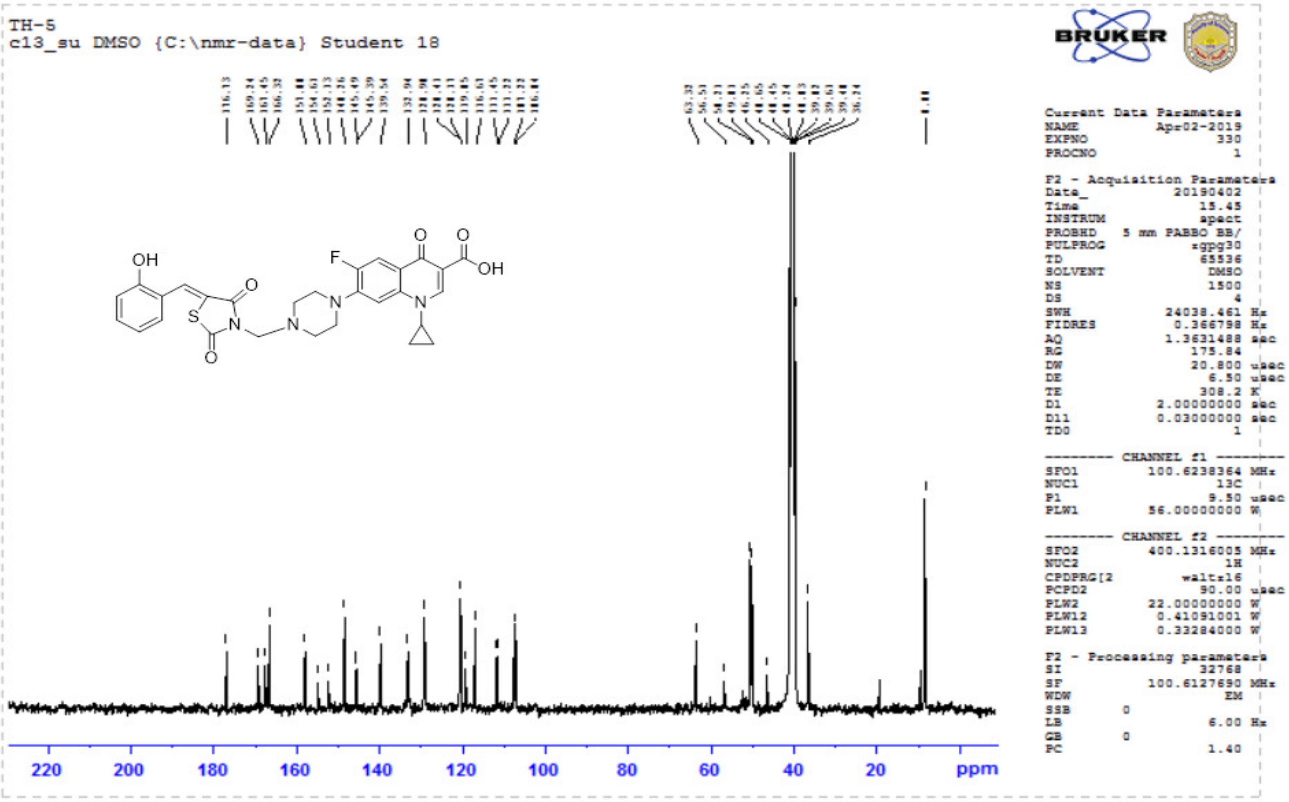

S20- ^{1}H, ^{13}C NMR and dept-135 Spectra of 20

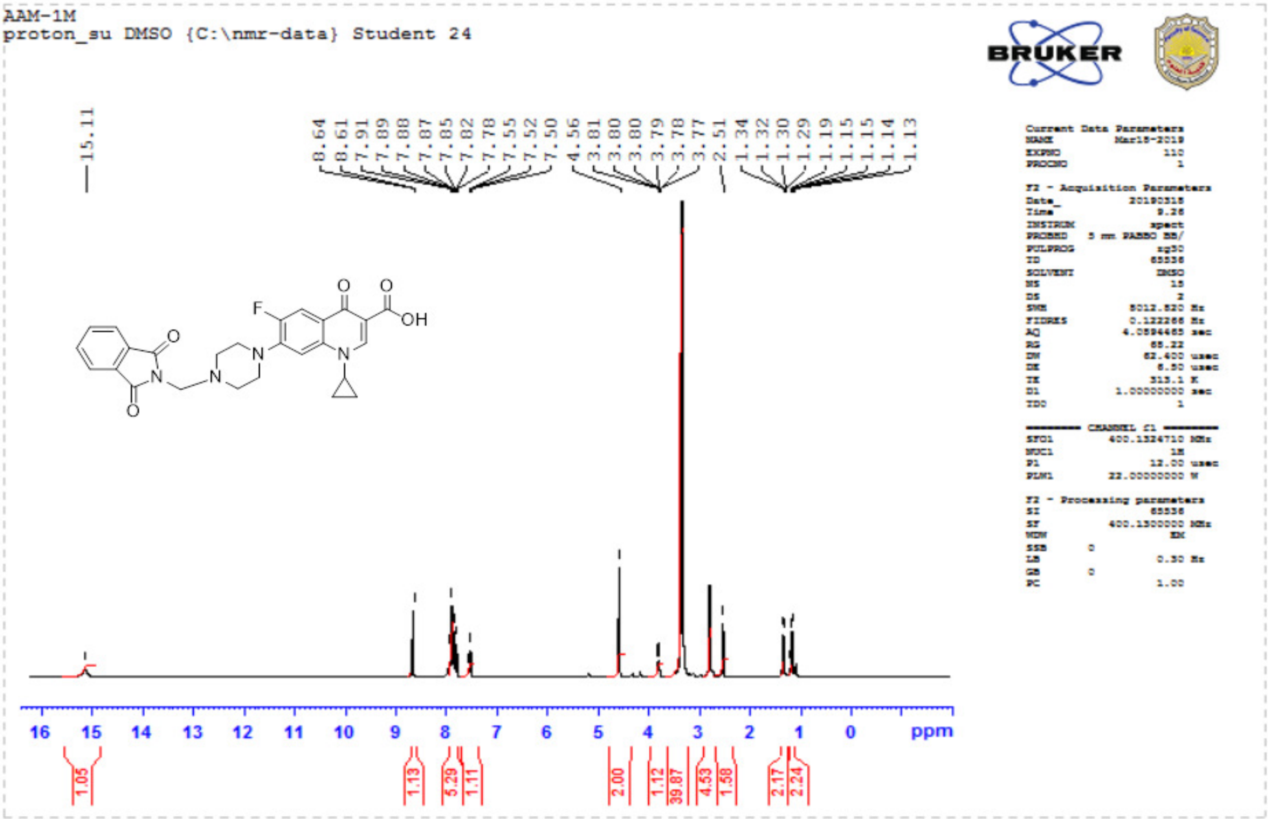

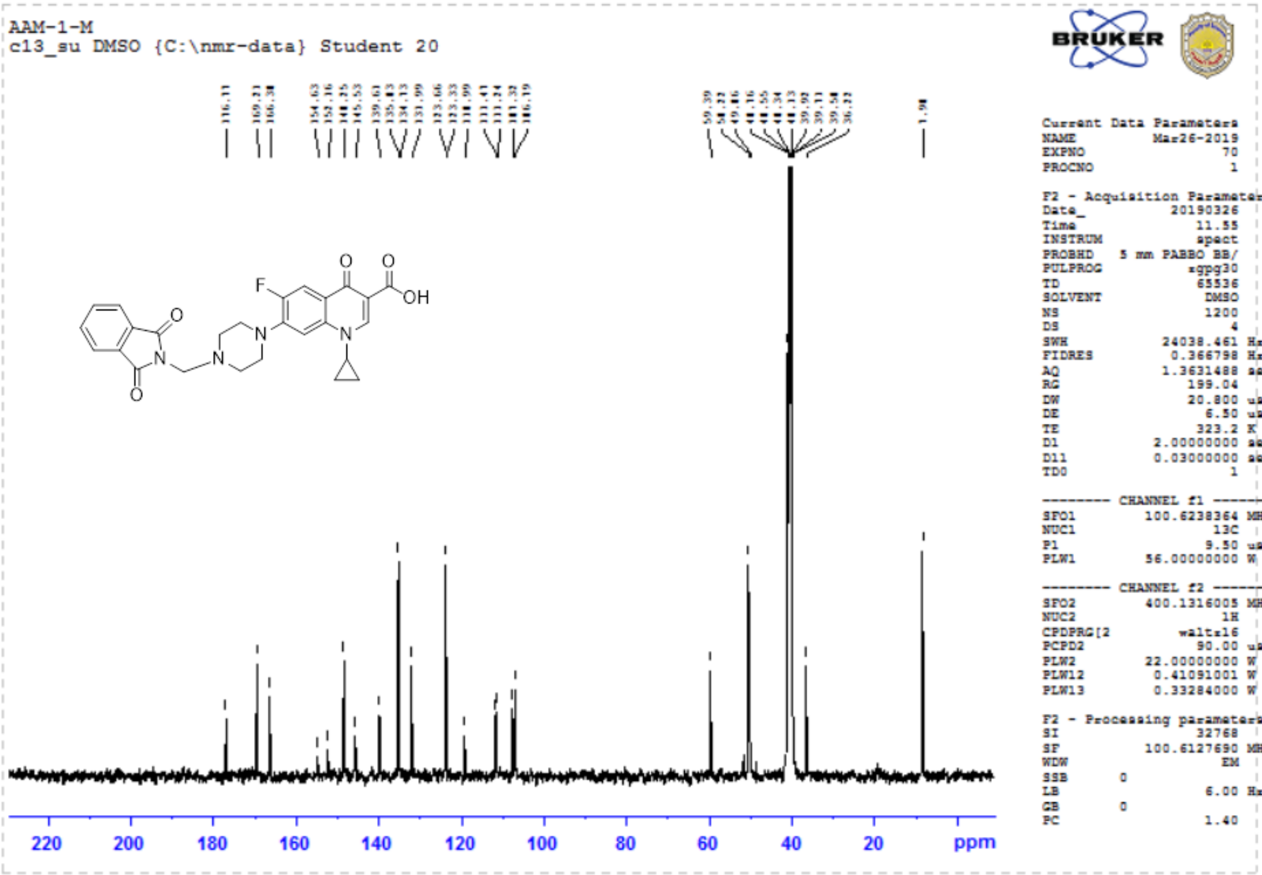

S21- ^{1}H, ^{13}C NMR and dept-135 Spectra of 21

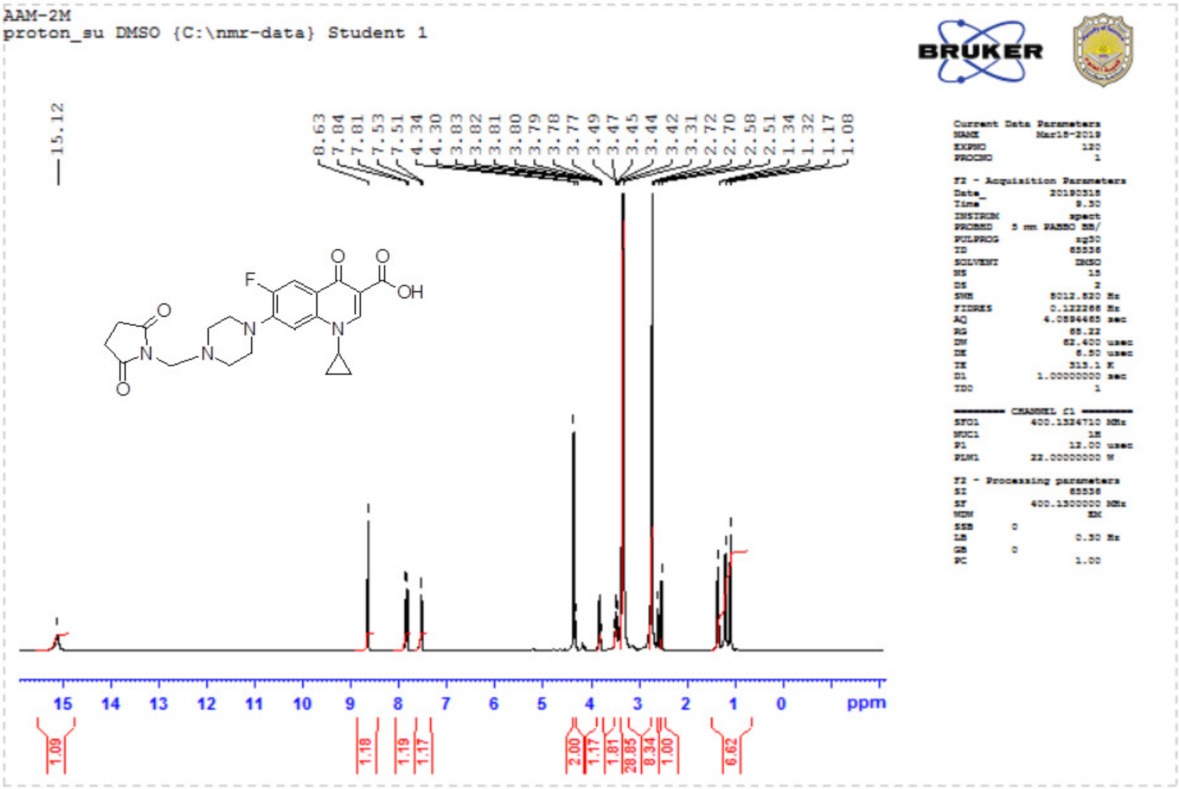

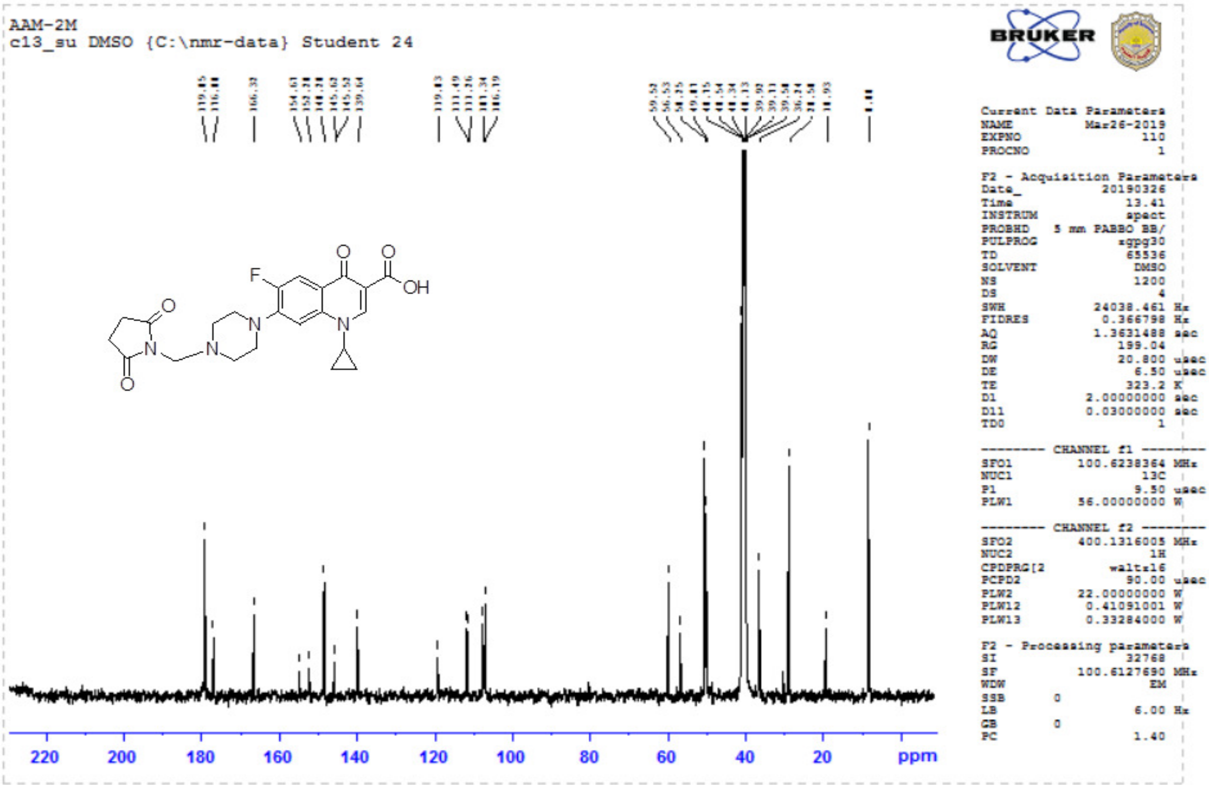

AAM-2M
c13_su DMSO {C:\nmr-data} Student 24
BRUKER
Current Data Parameters
NAME Mar26-2019
EXPNO 110
PROCNO 1
F2 - Acquisition Parameters
Date_ 20190326
Time 13.41
INSTRUM spect
PROBHD 5 mm PABBO BB/
PULPROG zgpg30
TD 65536
SOLVENT DMSO
NS 1200
DS 4
SWH 24038.461 Hz
FIDRES 0.366798 Hz
AQ 1.3631488 sec
RG 199.04
DW 20.800 usec
DE 6.50 usec
TE 323.2 K
D1 2.00000000 sec
D11 0.03000000 sec
TD0 1
CHANNEL f1
SFO1 100.6238364 MHz
NUC1 13C
P1 9.50 usec
PLW1 56.00000000 W
CHANNEL f2
SFO2 400.1316005 MHz
NUC2 1H
CPDPRG[2 waltz16
PCPD2 90.00 usec
PLW2 22.00000000 W
PLW12 0.41091001 W
PLW13 0.33284000 W
F2 - Processing parameters
SI 32768
SF 100.6127690 MHz
WDW EM
SSB 0
LB 6.00 Hz
GB 0
PC 1.40
220 200 180 160 140 120 100 80 60 40 20 ppm

Table S1

Molecular properties of compounds 1-21 predicted using Swiss ADME website.

Molecule	Comp 1	Comp 2	Comp 3	Comp 4	Comp 5	Comp 6	Comp 7
MW	521.56	556.01	571.62	522.55	522.55	522.55	596.67
Heavy atoms	37	38	41	37	37	37	43
Aromatic heavy atoms	21	21	25	21	21	21	27
Fraction Csp3	0.31	0.31	0.27	0.32	0.32	0.32	0.25
Rotatable bonds	6	6	6	6	6	6	7
H-bond acceptors	7	7	7	8	8	8	6
H-bond donors	1	1	1	1	1	1	1
MR	145.25	150.26	162.76	143.05	143.05	143.05	172.31
TPSA	128.83	128.83	128.83	141.72	141.72	141.72	120.62
iLOGP	3.26	3.37	4.21	2.81	2.81	2.81	4.02
XLOGP3	1.47	2.1	2.72	0.73	0.73	0.73	2.63
WLOGP	3.58	4.24	4.74	2.98	2.98	2.98	4.78
MLOGP	2.43	2.9	3.05	1.46	1.46	1.46	3.66
Silicos-IT Log P	3.58	4.23	4.58	3.02	3.02	3.02	4.19
Consensus Log P	2.87	3.37	3.86	2.2	2.2	2.2	3.86
ESOL Log S	-4.02	-4.62	-5.15	-3.56	-3.56	-3.56	-5.2
ESOL Solubility (mg/ml)	4.94E-02	1.32E-02	4.02E-03	1.43E-01	1.43E-01	1.43E-01	3.77E-03
ESOL Solubility (mol/l)	9.47E-05	2.38E-05	7.03E-06	2.73E-04	2.73E-04	2.73E-04	6.33E-06
ESOL Class	Moderately soluble	Moderately soluble	Moderately soluble	Soluble	Soluble	Soluble	Moderately soluble
Ali Log S	-3.78	-4.44	-5.08	-3.28	-3.28	-3.28	-4.81
Ali Solubility (mg/ml)	8.62E-02	2.04E-02	4.76E-03	2.71E-01	2.71E-01	2.71E-01	9.17E-03
Ali Solubility (mol/l)	1.65E-04	3.67E-05	8.33E-06	5.19E-04	5.19E-04	5.19E-04	1.54E-05
Ali Class	Soluble	Moderately soluble	Moderately soluble	Soluble	Soluble	Soluble	Moderately soluble
Silicos-IT LogSw	-5.81	-6.38	-7.42	-5.43	-5.43	-5.43	-7.4
Silicos-IT Solubility (mg/ml)	8.13E-04	2.31E-04	2.17E-05	1.93E-03	1.93E-03	1.93E-03	2.38E-05
Silicos-IT Solubility (mol/l)	1.56E-06	4.16E-07	3.80E-08	3.69E-06	3.69E-06	3.69E-06	3.98E-08
Silicos-IT class	Moderately soluble	Poorly soluble	Poorly soluble	Moderately soluble	Moderately soluble	Moderately soluble	Poorly soluble
GI absorption	High	Low	Low	Low	Low	Low	Low
BBB permeant	No	No	No	No	No	No	No
Pgp substrate	Yes	Yes	Yes	Yes	Yes	Yes	Yes
CYP1A2 inhibitor	No	No	No	No	No	No	No
CYP2C19 inhibitor	Yes	Yes	Yes	No	No	No	Yes
CYP2C9 inhibitor	Yes	Yes	Yes	Yes	Yes	Yes	Yes
CYP2D6 inhibitor	No	No	Yes	No	No	No	Yes
CYP3A4 inhibitor	No	Yes	No	Yes	Yes	Yes	No
log Kp (cm/s)	-8.44	-8.2	-7.86	-8.97	-8.97	-8.97	-8.07
Lipinski #violations	1	1	1	1	1	1	1

Ghose #violations	2	2	2	2	2	2	3
Veber #violations	0	0	0	1	1	1	0
Egan #violations	0	0	0	1	1	1	0
Muegge #violations	0	0	0	0	0	0	0
Bioavailability Score	0.55	0.55	0.55	0.55	0.55	0.55	0.55
PAINS #alerts	0	0	0	0	0	0	0
Brenk #alerts	1	1	1		1	1	1
Leadlikeness #violations	1	1	1		1	1	1
Synthetic Accessibility	3.88	3.93	4.12		3.92	3.92	4.28

Table S2

Molecule	Comp 8	Comp 9	Comp 10	Comp 11	Comp 12	Comp 13	Comp 14
MW	631.12	646.73	646.73	597.66	597.66	611.69	626.7
Heavy atoms	44	47	47	43	43	44	45
Aromatic heavy atoms	27	31	31	27	27	22	22
Fraction Csp3	0.25	0.22	0.22	0.26	0.26	0.27	0.27
Rotatable bonds	7	7	7	7	7	7	7
H-bond acceptors	6	6	6	7	7	6	7
H-bond donors	1	1	1	1	1	2	2
MR	177.32	189.82	189.82	170.11	170.11	182.83	185.63
TPSA	120.62	120.62	120.62	133.51	133.51	130.21	147.44
iLOGP	3.88	4.01	4.01	3.66	3.45	4.17	4.05
XLOGP3	4.17	4.79	4.79	2.47	1.56	2.17	1.61
WLOGP	5.43	5.93	5.93	4.18	4.18	2.64	2.23
MLOGP	4.11	4.22	4.22	2.7	2.7	2.46	2.5
Silicos-IT Log P	4.84	5.19	5.19	3.62	3.62	4.51	3.22
Consensus Log P	4.49	4.83	4.83	3.33	3.1	3.19	2.72
ESOL Log S	-6.37	-6.89	-6.89	-5.1	-4.53	-4.91	-4.64
ESOL Solubility (mg/ml)	2.68E-04	8.26E-05	8.26E-05	4.70E-03	1.76E-02	7.57E-03	1.44E-02
ESOL Solubility (mol/l)	4.24E-07	1.28E-07	1.28E-07	7.87E-06	2.94E-05	1.24E-05	2.29E-05
ESOL Class	Poorly soluble	Poorly soluble	Poorly soluble	Moderately soluble	Moderately soluble	Moderately soluble	Moderately soluble
Ali Log S	-6.41	-7.05	-7.05	-4.92	-3.97	-4.54	-4.32
Ali Solubility (mg/ml)	2.45E-04	5.70E-05	5.70E-05	7.22E-03	6.35E-02	1.77E-02	3.01E-02
Ali Solubility (mol/l)	3.88E-07	8.81E-08	8.81E-08	1.21E-05	1.06E-04	2.90E-05	4.81E-05
Ali Class	Poorly soluble	Poorly soluble	Poorly soluble	Moderately soluble	Soluble	Moderately soluble	Moderately soluble
Silicos-IT LogSw	-7.97	-9.01	-9.01	-7.03	-7.03	-8.12	-7.03
Silicos-IT Solubility (mg/ml)	6.76E-06	6.36E-07	6.36E-07	5.62E-05	5.62E-05	4.59E-06	5.84E-05
Silicos-IT Solubility (mol/l)	1.07E-08	9.84E-10	9.84E-10	9.40E-08	9.40E-08	7.50E-09	9.31E-08
Silicos-IT class	Poorly soluble	Poorly soluble	Poorly soluble	Poorly soluble	Poorly soluble	Poorly soluble	Poorly soluble
GI absorption	Low	Low	Low	Low	Low	High	Low
BBB permeant	No	No	No	No	No	No	No
Pgp substrate	Yes	No	No	Yes	Yes	Yes	Yes
CYP1A2 inhibitor	No	No	No	No	No	No	No
CYP2C19 inhibitor	Yes	Yes	Yes	Yes	Yes	Yes	No
CYP2C9 inhibitor	Yes	Yes	Yes	Yes	Yes	Yes	Yes
CYP2D6 inhibitor	No	No	No	Yes	Yes	Yes	Yes

CYP3A4 inhibitor	Yes	No	No	Yes	Yes	Yes	Yes
log Kp (cm/s)	-7.19	-6.84	-6.84	-8.19	-8.84	-8.49	-8.98
Lipinski #violations	1	2	2	1	1	1	1
Ghose #violations	3	4	4	3	3	3	3
Veber #violations	0	0	0	0	0	0	1
Egan #violations	0	1	1	1	1	0	1
Muegge #violations	1	2	2	0	0	1	1
Bioavailability Score	0.55	0.17	0.17	0.55	0.55	0.55	0.55
PAINS #alerts	0	0	0	0	0	0	0
Brenk #alerts	1	1	1	1	1	1	2
Leadlikeness #violations	2	2	2	1	1	1	1
Synthetic Accessibility	4.32	4.52	4.52	4.29	4.23	4.55	4.73

Table S3

Molecule	Comp 15	Comp 16	Comp 17	Comp 18	Comp 19	Comp 20	Comp 21
MW	626.7	548.59	583.03	578.61	564.58	490.48	442.44
Heavy atoms	45	39	40	41	40	36	32
Aromatic heavy atoms	22	16	16	16	16	16	10
Fraction Csp3	0.27	0.29	0.29	0.31	0.29	0.31	0.45
Rotatable bonds	7	6	6	7	6	5	5
H-bond acceptors	7	7	7	8	8	7	7
H-bond donors	2	1	1	1	2	1	1
MR	185.63	157.26	162.27	163.75	159.28	139.95	124.47
TPSA	147.44	128.46	128.46	137.69	148.69	103.16	103.16
iLOGP	4.05	3.74	4.07	3.94	3.31	2.81	2.36
XLOGP3	1.61	1.64	2.26	1.61	1.28	0.16	-1.6
WLOGP	2.23	3.1	3.75	3.1	2.8	1.76	0.62
MLOGP	2.5	2.05	2.51	1.48	1.55	1.72	1.11
Silicos-IT Log P	3.22	2.92	3.57	3	2.45	2.22	1.39
Consensus Log P	2.72	2.69	3.23	2.63	2.28	1.74	0.78
ESOL Log S	-4.64	-4.18	-4.78	-4.27	-4.05	-2.98	-1.48
ESOL Solubility (mg/ml)	1.44E-02	3.61E-02	9.71E-03	3.12E-02	5.07E-02	5.13E-01	1.48E+01
ESOL Solubility (mol/l)	2.29E-05	6.58E-05	1.66E-05	5.39E-05	8.98E-05	1.05E-03	3.34E-02
ESOL Class	Moderately soluble	Moderately soluble	Moderately soluble	Moderately soluble	Moderately soluble	Soluble	Very soluble
Ali Log S	-4.32	-3.95	-4.59	-4.11	-4	-1.88	-0.06
Ali Solubility (mg/ml)	3.01E-02	6.15E-02	1.48E-02	4.46E-02	5.62E-02	6.41E+00	3.88E+02
Ali Solubility (mol/l)	4.81E-05	1.12E-04	2.55E-05	7.70E-05	9.96E-05	1.31E-02	8.77E-01
Ali Class	Moderately soluble	Soluble	Moderately soluble	Moderately soluble	Moderately soluble	Very soluble	Very soluble
Silicos-IT LogSw	-7.03	-5.59	-6.16	-5.68	-4.99	-5.24	-3.33
Silicos-IT Solubility (mg/ml)	5.84E-05	1.42E-03	4.03E-04	1.22E-03	5.74E-03	2.85E-03	2.08E-01
Silicos-IT Solubility (mol/l)	9.31E-08	2.59E-06	6.92E-07	2.10E-06	1.02E-05	5.81E-06	4.70E-04
Silicos-IT class	Poorly soluble	Moderately soluble	Poorly soluble	Moderately soluble	Moderately soluble	Moderately soluble	Soluble
GI absorption	Low	High	High	Low	Low	High	High
BBB permeant	No	No	No	No	No	No	No
Pgp substrate	Yes	Yes	Yes	Yes	Yes	Yes	Yes
CYP1A2 inhibitor	No	No	No	No	No	No	No
CYP2C19 inhibitor	No	Yes	Yes	No	No	No	No
CYP2C9 inhibitor	Yes	Yes	Yes	Yes	Yes	Yes	No
CYP2D6 inhibitor	Yes	No	No	No	No	No	No
CYP3A4 inhibitor	Yes	Yes	Yes	Yes	Yes	No	No
log Kp (cm/s)	-8.98	-8.48	-8.25	-8.69	-8.84	-9.18	-10.13

Lipinski #violations	1	1	1	1	1	0	0
Ghose #violations	3	2	2	2	2	2	0
Veber #violations	1	0	0	0	1	0	0
Egan #violations	1	0	0	1	1	0	0
Muegge #violations	1	0	0	0	0	0	0
Bioavailability Score	0.55	0.55	0.55	0.55	0.55	0.55	0.55
PAINS #alerts	0	0	0	0	0	0	0
Brenk #alerts	2	2	2	2	2	1	1
Leadlikeness #violations	1	1	1	1	1	1	1
Synthetic Accessibility	4.73	4.13	4.12	4.24	4.2	3.3	3.1
